# Distinct Stromal Cell Factor Combinations Can Separately Control Hematopoietic Stem Cell Survival, Proliferation, and Self-Renewal

**DOI:** 10.1016/j.celrep.2014.05.014

**Published:** 2014-06-06

**Authors:** Stefan Wohrer, David J.H.F. Knapp, Michael R. Copley, Claudia Benz, David G. Kent, Keegan Rowe, Sonja Babovic, Heidi Mader, Robert A.J. Oostendorp, Connie J. Eaves

**Affiliations:** 1 Terry Fox Laboratory, British Columbia Cancer Agency, Vancouver, BC V5Z 1L3, Canada; 2 Landesklinikum Wr. Neustadt, Internal Medicine 1, Wr. Neustadt 2700, Austria; 3 3^rd^ Department of Internal Medicine, Klinikum Rechts der Isar, Technische Universität München, Munich 81675, Germany

## Abstract

Hematopoietic stem cells (HSCs) are identified by their ability to sustain prolonged blood cell production in vivo, although recent evidence suggests that durable self-renewal (DSR) is shared by HSC subtypes with distinct self-perpetuating differentiation programs. Net expansions of DSR-HSCs occur in vivo, but molecularly defined conditions that support similar responses in vitro are lacking. We hypothesized that this might require a combination of factors that differentially promote HSC viability, proliferation, and self-renewal. We now demonstrate that HSC survival and maintenance of DSR potential are variably supported by different Steel factor (SF)-containing cocktails with similar HSC-mitogenic activities. In addition, stromal cells produce other factors, including nerve growth factor and collagen 1, that can antagonize the apoptosis of initially quiescent adult HSCs and, in combination with SF and interleukin-11, produce >15-fold net expansions of DSR-HSCs ex vivo within 7 days. These findings point to the molecular basis of HSC control and expansion.

## Highlights

• HSC viability, mitogenesis, and self-renewal are differentially controlled
• Stromal cells produce nonmitogenic factors that directly sustain HSC viability
• More adult bone marrow cells can produce HSCs than display HSC activity directly
• Nerve growth factor and collagen 1 promote serially transplantable HSCs

## Graphical Abstract

**Figure:**
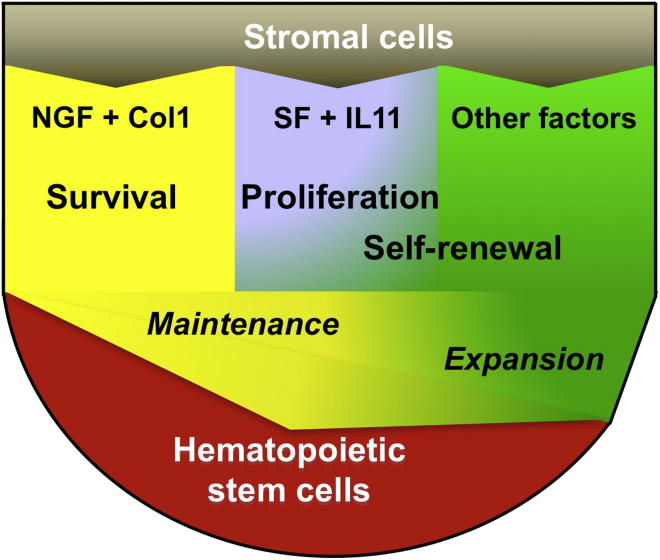

## Teaser

Wohrer et al. now show that different factors secreted by stromal cells separately control the survival, proliferation, and self-renewal of hematopoietic stem cells. These factors are thus required in combination to stimulate net expansions of these cells with full retention of their original stem cell properties.

## Introduction

Hematopoietic stem cells (HSCs) represent a rare subset of undifferentiated precursors of blood cells, historically recognized by their ability to regenerate large, self-sustaining clones of mature progeny in transplanted irradiated hosts. This property has been successfully exploited to interrogate molecular mechanisms that regulate the acquisition and maintenance of the HSC state. It is also the basis of widely used hematopoietic cell transplants in patients. Not surprising, therefore, is the intense interest in defining conditions that would stimulate significant HSC expansion in vitro. Although many genes important to HSC proliferation and self-renewal have now been characterized (Xie et al., 2014), a molecular signature that specifically defines the functional state of HSCs has not been identified. Likewise, culture conditions that support significant net expansions of normal HSCs with lifelong cell output activity remain lacking.

One limitation lies in the recently appreciated heterogeneity that characterizes populations historically classified as HSCs based on their ability to produce mature blood cells for at least 4 months in transplanted hosts (Benveniste et al., 2010, Benz et al., 2012, Dykstra et al., 2007, Kent et al., 2009, Morita et al., 2010, Sanjuan-Pla et al., 2013, Yamamoto et al., 2013). Serial transplants of clonally tracked HSCs have shown that only about half of HSCs thus defined will produce sufficient daughter HSCs in transplanted primary hosts to regenerate long-term hematopoiesis in secondary mice. HSCs possessing this durability of self-renewal activity (hereafter referred to as DSR-HSCs) are selectively enriched in the lineage marker-negative (Lin^−^) CD45^+^EPCR^+^Sca1^+^CD34^−^CD49b^low^CD48^−^CD150^2+^ fraction of adult mouse bone marrow (BM) cells. Biologically, DSR-HSCs are distinguished by a continuing robust ability to produce mature myeloid cells independent of their lymphopoietic activity. They include most HSCs we have previously subclassified as α- or β-HSCs, and a few as γ-HSCs (Benveniste et al., 2010, Benz et al., 2012, Dykstra et al., 2007, Kent et al., 2009, Morita et al., 2010). Conversely, more limited self-renewal (LSR) activity (identified by its failure to produce sufficient HSCs to repopulate secondary mice) is a property of all HSCs subclassified as δ-HSCs and many as γ-HSCs. LSR-HSCs are selectively enriched in the CD45^+^EPCR^+^Sca1^+^CD34^−^CD49b^hi^CD48^−^CD150^+/−^ fraction of adult mouse BM cells.

Survival, proliferation, and maintenance of stem cell properties are all actively regulated states of HSCs and hence likely to be important determinants of their expansion. These states are subject to regulation by external cues, some of which are provided in vivo by BM stromal cells (Mercier et al., 2012). HSC survival and, to a limited extent, self-renewal can be supported by BM stromal cells (Dexter et al., 1977, Fraser et al., 1992) or factors they secrete, including Steel factor (SF), interleukin-11 (IL-11), Flt3 ligand, Wnt3a, angiopoietin-like proteins (Angptls), thrombopoietin (TPO), fibroblast growth factor 1 (FGF1), and insulin growth factor-binding protein 2 (IGFBP2) (Audet et al., 2002, Huynh et al., 2008, Kent et al., 2008, Miller and Eaves, 1997, Reya et al., 2003, Zhang et al., 2006). However, to date, large net expansions of DSR-HSCs ex vivo have not been achieved using defined factors, and the relative roles of different factors in promoting DSR-HSC viability, proliferation, and self-renewal are not understood.

To elucidate mechanisms by which stromal cells regulate key functions of HSCs, we chose the urogenital ridge-derived UG26-1B6 (UG26) cell line as a source of additional external cues because it had been found to be exceptionally potent in supporting HSCs in a contact-independent fashion (Oostendorp et al., 2002, Oostendorp et al., 2005). As targets, we used CD45^+^EPCR^+^CD48^−^CD150^+^ (ESLAM) adult mouse cells (∼40% pure HSCs; Kent et al., 2009). Our results identify nerve growth factor (NGF) and collagen 1 (Col 1) as additives that can optimize DSR-HSC survival in a defined serum-free medium (SFM) and also synergize with the mitogenic and self-renewal-promoting activity of SF and IL-11 to achieve an unprecedented expansion of total HSCs while maintaining input DSR-HSC numbers.

## Results

### Stromal Cell-Derived Factors Enhance the SF Plus IL-11-Stimulated Expansion of DSR-HSCs

**Figure 1:**
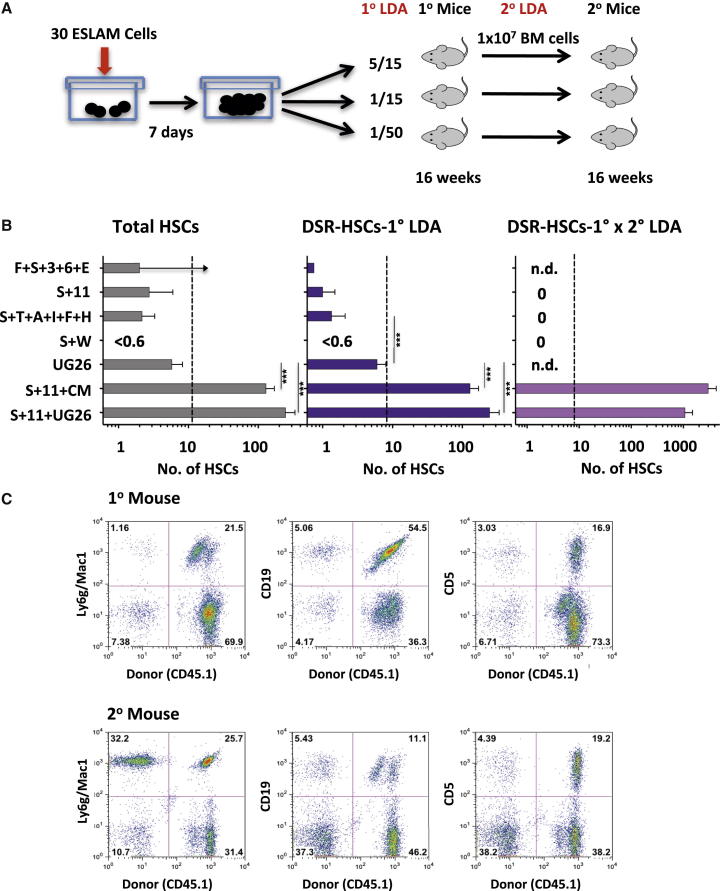
HSC Numbers Produced in 7-Day Cultures of ESLAM Cells Containing Different Supplements (A) Experimental design. (B) Results of 16-week limiting dilution transplant assays used to determine the outputs of HSCs and DSR-HSCs (left 2 panels) and the cumulative DSR-HSC expansion obtained first in vitro and then in primary (1°) recipients (right panel) (12 mice/condition/experiment, 3–5 experiments/condition, mean and SEM for each condition). Dotted lines show the total and DSR-HSCs estimated to be present in the 30 input ESLAM cells. Holm-corrected pairwise significance values are shown (^∗^p = 0.05; ^∗∗^p = 0.01; ^∗∗∗^p < 0.001). Where a limiting dilution was not reached, the bar indicates the minimal HSC value detectable with an upward arrow. Supplements were as follows: F+S+3+6+E (15% FBS plus 50 ng/ml SF plus 10 ng/ml IL-3 plus 10 ng/ml IL-6 plus 3 U/ml Epo); S+11 (100 ng/ml SF plus 20 ng/ml plus IL-11); S+T+A+I+F+H (10 ng/ml SF plus 20 ng/ml TPO plus 100 ng/ml Angptl3 plus 500 ng/ml IGFBP2 plus 10 ng/ml FGF1 plus 10 μg/ml heparin [H]); S+W (30 ng/ml SF plus 100 ng/ml Wnt3a); UG26 cells; CM (50% UG26 CM); and S+11+CM (SF plus IL-11 plus CM). (C) Representative FACS profiles of PB cells obtained 16 weeks after transplanting primary and secondary mice with cells harvested from cultures containing UG26 cells plus SF plus IL-11, described in (B). The following markers were used to investigate donor chimerism: Ly6g/Mac1 (GM cells), CD19 (B cells), and CD5 (T cells).

**Figure 2:**
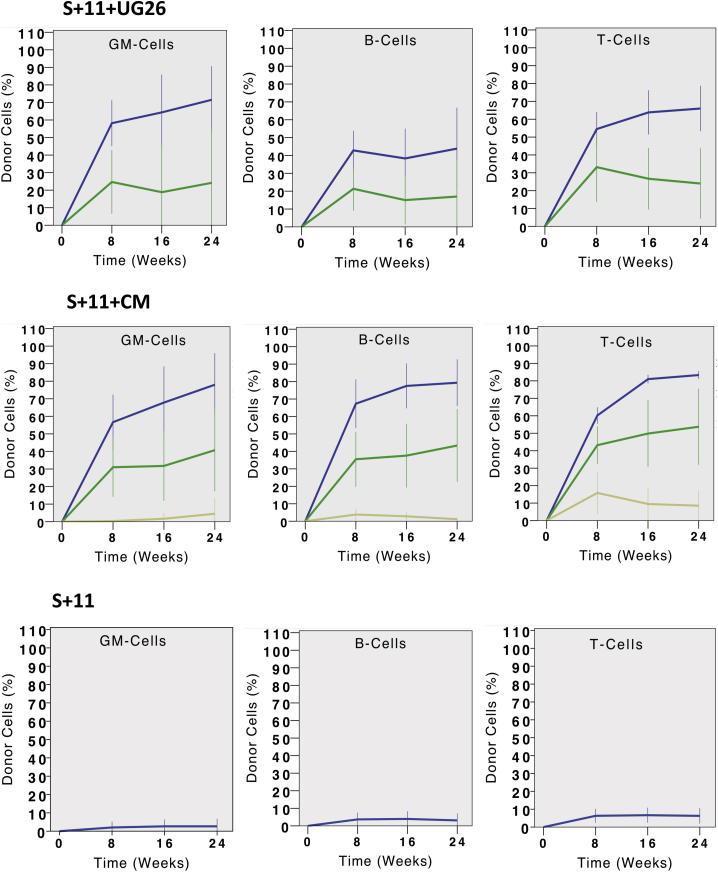
Different Reconstitution Kinetics in Mice Transplanted with Matched Progeny Outputs of ESLAM Cells Cultured for 7 Days with Different Stimuli Geometric mean percentage donor contributions to the total circulating GM, B, and T cells measured at different times after transplanting groups of mice with the progeny of 30 ESLAM cells cultured as described in Figure 1. Error bars indicate the range defined by ±2 SEM. Blue, green, and brown lines indicate mice that received 1/15^th^, 1/50^th^, and 1/90^th^ of a culture each, respectively.

To first compare the DSR-HSC-stimulating activity of various additives reported to support adult mouse BM HSC expansion in vitro, we set up test cultures with 30 ESLAM cells each and then 7 days later, performed limiting dilution transplant assays to determine the numbers of DSR-HSCs, as well as the total HSCs present (Figure 1A). HSCs were defined as cells whose progeny constituted >1% of all the nucleated peripheral blood (PB) cells present 16–24 weeks posttransplant. DSR-HSCs were defined as the subset of HSCs that generated >1% of all the circulating granulocyte and monocyte (GM) cells present 16–24 weeks posttransplant (and LSR-HSCs as all the other HSCs). Single-cell transplants indicated that the 30 input ESLAM cells contained, on average, 12 total HSCs of which 8 were DSR-HSCs. The results for the cultured cells showed that DSR-HSC numbers increased significantly above input (29-fold; p < 0,001) when UG26 cells were present together with SF plus IL-11 and to a slightly, but not significantly (p = 0.19), lesser extent (15-fold) when the UG26 cell conditioned medium (CM) was added instead of the UG26 cells. Total HSC numbers were similarly and also significantly increased in both of these cultures: 20- and 11-fold, respectively (p < 0.001; Figure 1B, left and middle panels). Representative fluorescence-activated cell sorting (FACS) profiles of cells regenerated in recipients of cells cultured in SF plus IL-11 plus UG26 cells are shown in Figure 1C, and lineage-specific reconstitution kinetics of donor cells cultured in SF plus IL-11 plus UG26 cells, or SF plus IL-11 plus CM, or SF plus IL-11 only, are shown in Figure 2. Interestingly, ESLAM cells cultured in UG26 cells alone showed maintenance but not expansion of DSR-HSC numbers (and of total HSCs, Figure 1B). In contrast, DSR-HSC numbers showed a net decrease when cultured in any of the four other factor combinations tested, although in some, total HSC numbers were maintained at, or close to, input levels (Figure 1B).

To evaluate more stringently the DSR property of the in-vitro-expanded HSCs, we determined the number of additional daughter HSCs produced in the primary recipients of the cultured cells. This involved performing another set of limiting dilution HSC transplant assays on BM cells harvested from each group of primary recipients 24 weeks after they had been initially transplanted with cells harvested from the 7-day cultures. The results showed that cultures to which either UG26 cells or UG26 CM had been added to the SF plus IL-11 cocktail produced HSCs in vitro that were capable of extensive further expansion in the primary hosts (Figure 1B, right panel) and, hence, gave a high level of repopulation of secondary recipients (Figure 1C, lower panels). Thus, the overall DSR-HSC expansion achieved (first in vitro, and then in the primary recipients) when UG26 cells were present together with SF plus IL-11 was 130-fold, and 360-fold when UG26 CM was added (assuming one femur represents 5% of the total BM of a mouse; Colvin et al., 2004). These sustained HSC expansions obtained with UG26 cells or CM were again not significantly different from each other (p = 0.13). In contrast, no secondary repopulating activity was detected in comparable assays of BM cells from primary recipients of cells cultured with any of the defined growth factor cocktails.

These findings document the ability of factors produced by UG26 cells in combination with SF plus IL-11 to produce a rapid and significant net expansion in vitro of serially transplantable HSCs. They also demonstrate that this effect can be mediated by a mechanism that is cell contact independent.

### UG26 Cells Produce Factors that, in Combination with SF Plus IL-11, Enable All HSC Differentiation Programs to Be Sustained

**Figure 3:**
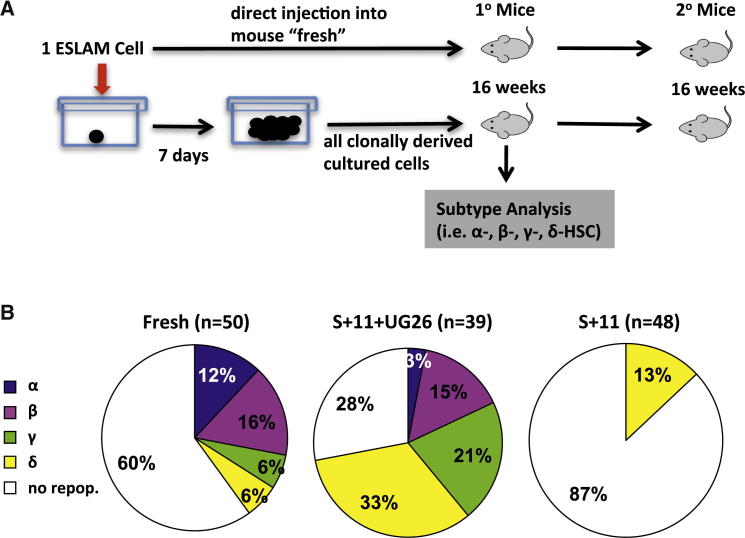
Effect of Different Supplements on the Frequency of Single ESLAM Cells that Generate HSCs in 7-Day Cultures (A) Experimental design. (B) Frequency of HSCs in each sample and the differentiation patterns obtained from them. Left pie chart shows input ESLAM population (50 cells tested). Middle pie chart shows 7-day cultures initiated with single ESLAM cells stimulated with UG26 cells plus SF plus IL-11 (39 clones tested). Right pie chart shows 7-day cultures initiated with single ESLAM cells stimulated with SF plus IL-11 only (48 clones). The definitions used to distinguish α, β, γ, and δ patterns of differentiation are given in the Supplemental Experimental Procedures. Results are pooled from three to six experiments. See also Figure S1 for secondary transplant results. repop., repopulation.

To determine the frequency of ESLAM cells that can generate transplantable HSC progeny in the presence of SF plus IL-11 with or without UG26 cells, we set up a second series of cultures with a single ESLAM cell each and then transplanted the entire contents of each (regardless of how many cells it contained) into separate irradiated recipients (Figure 3A). Analysis of the PB of these mice 16 weeks later showed that both α and β patterns of differentiation were obtained from the cultures to which UG26 cells had been added and at a frequency not significantly different from the frequency of α- and β-HSCs in the input ESLAM cells (18% versus 28%; p = 0.12; Figure 3B). Interestingly, the proportion of cultures that contained any type of HSC was significantly higher than the frequency of total HSCs in the original ESLAM cells (72% versus 40%; p < 0.001; Figure 3B). Thus, some ESLAM cells that are not directly detectable as HSCs can, nevertheless, generate progeny that have the functional properties of HSCs in vivo. In contrast to the cultures that contained SF plus IL-11 plus UG26 cells, only 13% of the cultures that contained only SF plus IL-11 contained any HSCs (a value significantly <40%, which was the input HSC frequency; p < 0.001), and all of these latter HSCs produced a δ pattern of reconstitution.

**Figure 4:**
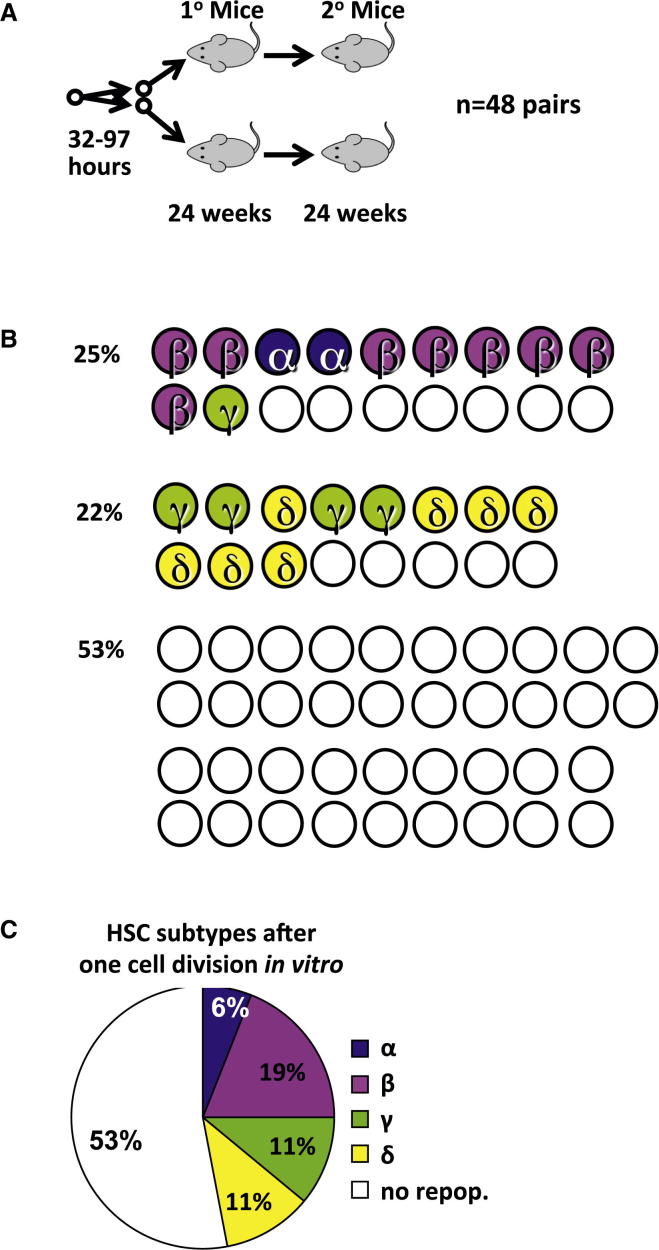
UG26 CM Enhances the Retention of DSR-HSC Functionality in the First Division Progeny of Single ESLAM Cells (A) Experimental design. A total of 36 ESLAM cells were cultured as single cells in UG26 CM plus SF plus IL-11 and 12 in SF plus IL-11 only. Both cells in doublets produced 32–97 hr later were then assayed individually for HSC activity in separate mice. Twenty-four weeks later, BM cells from DSR-HSC-repopulated primary mice were harvested and secondary transplantations performed. (B) Distribution of α, β, γ, or δ HSC subtypes in each first-generation pair of ESLAM daughter cells in which at least one daughter cell was an HSC of any type. Because none of the 24 mice that received a cell cultured in SF plus IL-11 showed engraftment, only results for cells cultured in UG26CM plus SF plus IL-11 are shown. (C) Distribution of the types of inferred input ESLAM cells classified according to the α, β, γ, or δ HSC subtypes that they produced in their first-generation progeny, as shown in (B). When both progeny were HSCs, the initial ESLAM cell was classified as the more primitive subtype (α, β, γ, δ—in that order).

As a more direct test of the frequency of ESLAM cells that are responsive to the factors produced by UG26 cells in concert with SF plus IL-11, we set up another series of single-cell cultures, in this case, with SF plus IL-11 with or without UG26 CM (36 with and 12 without UG26 CM; Figure 4A). These were visually monitored every few hours for the next 4½ days until a first division occurred. Pairs of daughter cells were thus identified and then separately transplanted pairwise into each of two irradiated recipients. In the 36 pairs of cells produced in the cultures containing SF plus IL-11 plus UG26 CM, 17 (47%) were found to contain at least 1 HSC, and 9 of these pairs (25%) produced at least 1 α- or β-HSC (Figure 4B). Overall, the frequencies and distributions of HSC subtypes in the first division progeny were similar to the HSCs both in the starting ESLAM cells and in the 7-day clones generated under similar conditions (Figure 4C). Secondary limiting dilution transplantation assays of the cells regenerated from the first division progeny pairs confirmed their DSR and LSR attributes assigned on the basis of their clonal GM contributions in the primary mice (Table 1; Figure S1). In contrast, none of the 24 mice injected with the 12 pairs of first division progeny of cells cultured in SF plus IL-11 was repopulated. From the paired daughter cell tracking, we also found more HSCs present in the progeny of cells that completed a first division after >48 hr in culture (67% versus 16% for those that completed a first division in <48 hr; p = 0.007) and reached 100% for cells that did not divide until after 96 hr.

**Figure 5:**
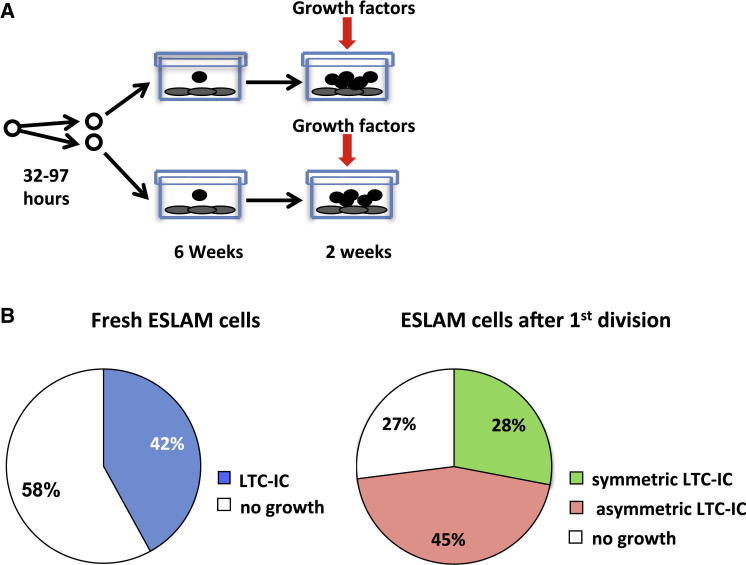
UG26 CM and SF plus IL-11 Support the Frequent Production of LTC-ICs in Both Progeny of Single ESLAM Cells Stimulated to Divide In Vitro (A) Experimental design. A total of 96 ESLAM cells were cultured as single cells in UG26 CM and SF plus IL-11 until they divided a first time as in Figure 4. Doublets were then transferred into 192 separate wells containing UG26 cells and assayed for LTC-IC activity. (B) Pie charts showing the frequencies of LTC-ICs (light-blue fraction) as determined from assays of single freshly isolated ESLAM cells (left), and the frequencies of ESLAM cells that produced two, one, or no LTC-ICs in their first division progeny (right).

We also determined the effect of adding UG26 CM to SF plus IL-11 on the proportion of first division progeny of ESLAM cells that would display long-term culture-initiating cell (LTC-IC) activity in a 6-week assay (Figure 5A). The frequency of LTC-ICs in the starting ESLAM cells (89 out of 213 [42%], Figure 5B) was similar to the frequency of total HSCs (40%; p = 0.87; Figure 3B), and the frequency of ESLAM cells that produced at least 1 daughter LTC-IC was again higher than the input LTC-IC frequency (70 out of 96 [73%]; p < 0.001) and also higher than the frequency of ESLAM cells that generated at least 1 daughter HSC (47%; p < 0.001; Figure 3D). The frequency of pairs in which both daughter cells were LTC-ICs (28%) was also higher than the frequency of pairs containing two HSCs (14%; p = 0.023). These disparities in the apparent effects of UG26 CM plus SF plus IL-11 on LTC-ICs and HSCs could be due to the selective inability of HSCs to engraft when they are in S/G_2_/M phases of the cell cycle (Bowie et al., 2006) but may also reflect a broader range of cells detected as LTC-ICs.

### Different Factors Separately Regulate HSC Survival and Mitogenesis

**Figure 6:**
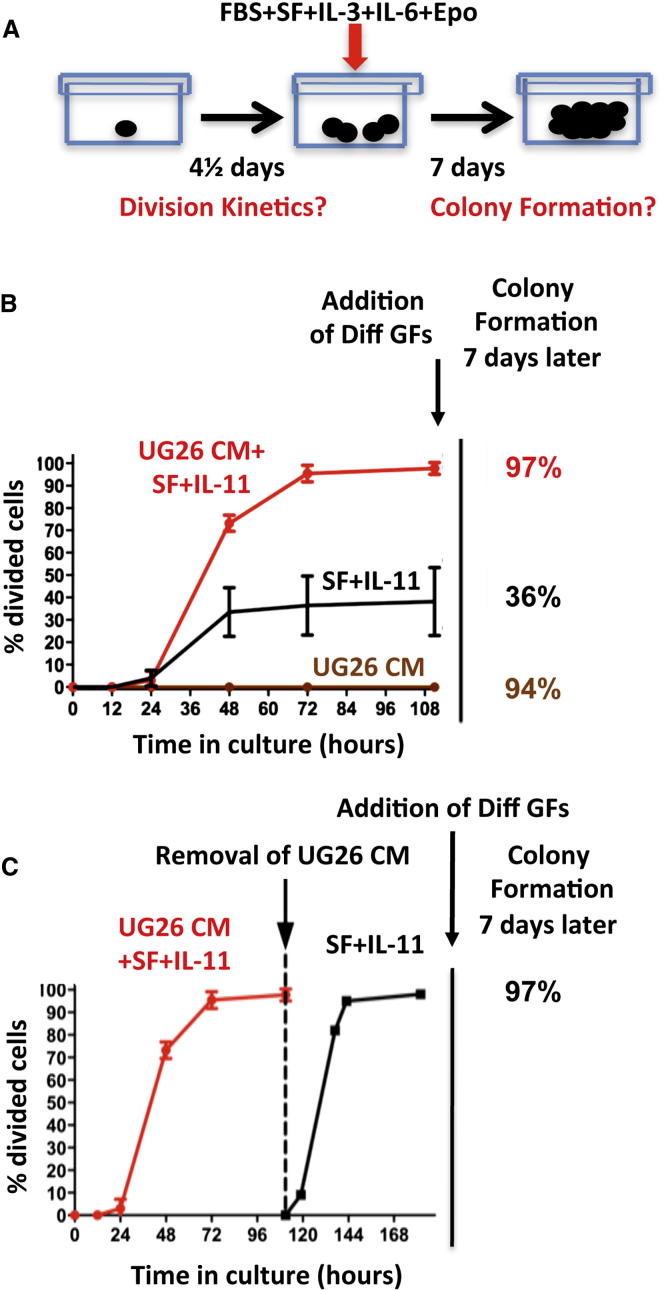
Comparison of the Different Mitogenic and Prosurvival Activities of UG26 CM, SF plus IL-11, and the Combination on ESLAM Cells in Single-Cell Cultures (A). Experimental design. (B) Cumulative plots of the kinetics with which single ESLAM cells completed a first division when cultured either in UG26 CM+SF+IL-11, or SF+IL-11 only, or UG26 CM (CM) only (5 experiments, 96 cells in each). Error bars show 95% confidence intervals (CI). Addition of Diff GFs, addition of different growth factors. (C) Plot showing the kinetics with which single ESLAM cells completed a next division after they had been cultured for an initial 108 hr in UG26 CM+SF+IL-11 (i.e., until each cell had already divided at least once), when SFM and SF+IL-11 were added to replace the initially added UG26 CM+SF+IL-11 (after washing the cells twice with SFM). Results are from 3 experiments (96 cells in each).

To interrogate the biological mechanism(s) by which the factors produced by UG26 cells might enable an in vitro expansion of DSR-HSC numbers, we initiated another series of cultures with single ESLAM cells and then monitored them visually at intervals over the next 4½ days (108 hr) to track the persistence of viable (refractile) cells and their rate of entry into a first division during that period (Figure 6A). At the end of 4½ days, we added medium containing fetal bovine serum (FBS) plus SF plus IL-3 plus IL-6 plus erythropoietin (Epo) as a further stimulus to promote the formation of readily detectable differentiating clones from persisting viable cells.

In the presence of SF plus IL-11 plus UG26 CM, 97% of the input cells (279 out of 288 cells in 3 experiments) survived and executed a first division between 24 and 108 hr after being placed in vitro (Figure 6B). Results were indistinguishable for 168 single ESLAM cells cultured in FBS plus SF plus IL-3 plus IL-6 plus Epo (96% survival with clonogenic activity), despite the inability of these conditions to support the retention of DSR-HSC activity (Figure 1A). In contrast, 64% (184 out of 288) of the single ESLAM cells cultured in SF plus IL-11 alone could no longer be visualized at the end of the first 12 hr in vitro. However, the remaining 36% of these cells remained refractile and appeared viable for the first 18 hr and then began to divide with the same kinetics as the cells maintained in SF plus IL-11 plus UG26 CM. These (and only these) went on to produce large colonies when the FBS plus SF plus IL-3 plus IL-6 plus Epo cocktail was added at the end of the first 4½ days. Interestingly, 90% (259 out of 288) of the cells initially cultured in UG26 CM alone (without SF plus IL-11) did not divide, and most also became smaller over time. Nevertheless, at the end of the first 4½ days, all of these cells plus another 12 thought to be dead (i.e., a total of 271 of the original 288) could still be stimulated to produce readily detectable colonies upon the addition of the FBS plus SF plus IL-3 plus IL-6 plus Epo cocktail.

To determine whether the survival advantage afforded by factors present in UG26 CM might be restricted to cells that are quiescent, we set up new single ESLAM cell cultures in SF plus IL-11 plus UG26 CM for 4½ days (by which time every cell had completed at least one division) and then switched the medium to SFM with SF plus IL-11 alone for a second 4½ days. Continued monitoring of each of these wells over the second 4½ days showed that 97% (279 out of 288) of the wells contained cells that continued to divide. Moreover, the range of times taken to complete the next division was the same as for the first division initiated in SF plus IL-11 plus UG26 CM, discounting the initial lag, and further addition of FBS plus SF plus IL-3 plus IL-6 plus Epo medium at the end of the second period of monitoring showed that 97% of the cultures again produced a very large clone over the following 7 days (Figure 6C).

These results indicate that SFM containing SF plus IL-11 only is unable to support the survival of a large proportion of quiescent adult HSCs, although once activated, both their viability and their proliferation can be efficiently sustained by continued exposure to SF plus IL-11. However, this initial loss of quiescent adult BM ESLAM cells can be circumvented by exposure to UG26 CM or FBS plus IL-3 plus IL-6 plus Epo, although these two sources of prosurvival factors clearly differ in their mitogenic activities and in their abilities to sustain HSC self-renewal activity. Interestingly, the overall rate at which initially quiescent ESLAM BM cells enter the cell cycle appeared independent of any of these conditions of stimulation.

### NGF and Col 1 Can Partially Replace UG26 CM in Maintaining DSR Activity during HSC Expansion In Vitro

**Figure 7:**
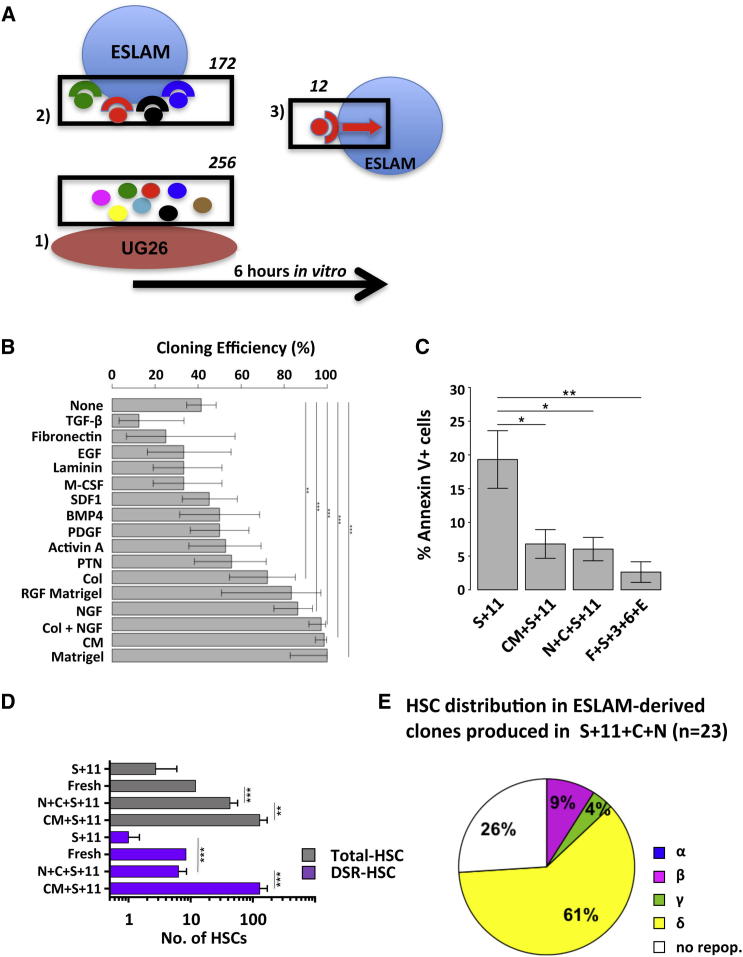
Col 1 and NGF Can Substitute for UG26 CM to Support DSR-HSC Self-Renewal In Vitro (A) Graphical display of the algorithm used to identify significantly changed pathways from Affymetrix gene chip data obtained on stromal cells and input and responding ESLAM cells. Numbers above the boxes indicate the number of sequentially identified genes. Additional details are provided in the text and Tables S1–S3. (B) Comparison of the ability of different factors to enhance the survival and mitogenesis of isolated ESLAM cells cultured in SFM with SF plus IL-11 for 7 days; 12–144 cells analyzed per test condition. A 95% CI was generated using the R function “prop.test.” (C) Proportion of Annexin V^+^ cells in 36 hr cultures of ESLAM cells incubated in SFM plus the additives shown. Mean ± SEM values from four experiments are shown. (D) Outputs of HSCs from 7-day cultures initiated with 30 freshly isolated ESLAM cells compared to input values using the same experimental design as in Figure 1A (12 mice/condition/experiment and 3–5 experiments/condition). White bars indicate total HSCs, and purple bars show DSR-HSCs. Values shown are the mean ± SEM. Additions were SF+IL-11 (S+11), NGF+Col 1+SF+IL-11 (N+C+S+11), or UG26 CM+SF+IL-11 (CM+S+11) at the same concentrations as in Figure 1. A 95% CI was generated using ELDA. (E) Distribution of HSC activity in clones derived from 23 single ESLAM cells cultured in SF plus IL-11 plus Col 1 plus NGF for 7 days determined from 16-week transplantation assays of single clones transplanted into individual mice. In (B) and (D), Holm-corrected pairwise significance values are shown (^∗^p = 0.05; ^∗∗^p = 0.01; ^∗∗∗^p < 0.001).

As a first step toward identifying the prosurvival factors produced by UG26 cells, we looked for pathways that they differentially activate as well as related candidate effectors secreted by UG26 cells and their possible receptors in purified adult BM ESLAM cells. To this end, we generated gene expression profiles for adult BM ESLAM cells before and 6 hr after being placed in culture with or without UG26 CM with or without SF plus IL-11. The 6 hr time point was chosen to obtain cells before evidence of their death is obvious when they are cultured in SF plus IL-11 alone (Lecault et al., 2011). We then compared these profiles with each other, as well as with a published gene expression profile for UG26 cells (Ledran et al., 2008) (Figure 7A).

Analysis of the profiles obtained for ESLAM cells stimulated with UG26 CM (with or without SF plus IL-11) compared to fresh ESLAM cells and ESLAM cells stimulated with SF plus IL-11 alone identified a total of 250 (of 430 tested) REACTOME pathways for which some members showed significantly altered transcript expression (p < 0.05) in cells maintained in UG26 CM with or without SF plus IL-11 (Table S1). These pathways included cell-cycle progression and metabolic pathways, signaling pathway activation, apoptosis-related pathways, and RNA processing/splicing pathways (Table S2). To identify specific candidate factors, we used Gene Ontology annotations to select transcripts present in UG26 cells that were categorized as encoding proteins in the “extracellular region” and that had anticipated interactions with expressed genes annotated as having “receptor activity” in the ESLAM cells. From this analysis, we identified 172 candidate factors (Table S3). From a survey of the current stem cell literature, a comparison of candidate factors to the differential expression of downstream pathways, and factor availability, we then selected a subset of 12 of the 172 candidate proteins for further testing of their ability to block the death of HSCs as previously shown for UG26 CM (Figure 6A). Among the 12 candidates tested, we found NGF and Col 1 to be the most effective substitute for UG26 CM (Figure 7B), and when present together, 97% of the input ESLAM cells remained viable. Both NGF and Col 1 also mimicked the complete lack of mitogenic activity of the UG26 CM (data not shown).

Assessment of ESLAM cells in 36 hr cultures containing SF plus IL-11 with or without UG26 CM, NGF, and Col 1, or FBS plus SF plus IL-3 plus IL-6 plus Epo showed that many cells (20%) were Annexin V^+^ when only SF plus IL-11 was present, i.e., only slightly fewer than the proportion of dead cells determined both by immediate visual inspection and by their subsequent failure to form colonies in the presence of added growth factors (Figure 6B). In contrast, the proportion of Annexin V^+^ cells was significantly decreased (p ≤ 0.03) in all cultures to which the prosurvival factors were added, including NGF plus Col 1 (Figure 7C).

To determine whether NGF and Col 1 can replace the ability of UG26 CM to promote HSC self-renewal divisions in vitro in the presence of SF plus IL-11, we set up a final series of 7-day cultures with 1 or 30 ESLAM cells each and assayed the HSC output using the same protocol as in Figure 1A. Primary recipients of these cultured cells showed that the addition of NGF plus Col 1 to SF plus IL-11 maintained input numbers of DSR-HSCs in the 7-day cultures and produced a 4-fold net expansion of total HSCs (p = 0.48 and < 0.001, respectively; Figure 7D). Although these values are not as high as those achieved with UG26 CM (p < 0.001 and = 0.01, respectively; Figure 1B), secondary transplant assays confirmed the continuing DSR activity of the HSCs maintained using NGF plus Col 1 (data not shown). Analyses of another 23 mice transplanted with clones generated from single ESLAM cells cultured for 7 days in SF plus IL-11 plus NGF plus Col 1 showed that 17 of the clones (74%) contained HSCs, and 3 clones (13%) contained DSR-HSCs that included the DSR-HSC-associated β pattern of differentiation (Figure 7E).

## Discussion

### Stromal Cells Produce Factors that Synergize with SF and IL-11 to Promote DSR-HSC Expansion In Vitro

Evidence that HSCs are regulated by nonhematopoietic stromal cells in vivo dates back many decades to transplantation experiments performed with *Sl/Sl*^*d*^ mice (McCulloch et al., 1965, Sutherland et al., 1970). Many products of stromal cells and related cell types have now been implicated in the regulation of the HSC compartment in vivo (Mercier et al., 2012). Likewise, the most successful strategies for maintaining HSCs long term in vitro have involved their coculture with primary stromal cells or stromal cell lines from various sources (Dexter et al., 1977, Fraser et al., 1990, Fraser et al., 1992, Moore et al., 1997, Ploemacher et al., 1989, Wineman et al., 1996). The recent identification of distinct subsets of stromal cells with variable roles in regulating different HSC functions in adult BM now raises the interesting possibility that they regulate HSCs via non (or incompletely)-overlapping mechanisms (Ding and Morrison, 2013, Guezguez et al., 2013, Kunisaki et al., 2013).

Here, we identify different combinations of factors secreted from stromal cells that differentially support biologically distinct HSC functions. These functions are controlled intrinsically by separate, albeit likely interconnecting, pathways, although all are important to the speed and extent to which HSCs can expand their numbers. Specifically, they are responsible for the maintenance of HSC viability, the response of HSCs to factors that control their cycling state, and the maintenance in HSCs of a continuing poised, but undifferentiated, state. Clonal analysis and secondary transplant assays of the cells produced in vitro under conditions that support all three of these functions (i.e., SF plus IL-11 plus either UG26 cells or UG26 CM or NGF plus Col 1) demonstrated a significant net expansion of adult mouse HSCs with either maintenance or expansion of HSCs with DSR properties. In contrast, in the absence of UG26 cells or UG26 CM or NGF plus Col 1, even full maintenance of ESLAM (and hence HSC) survival and mitogenesis (as could be achieved by exposure to FBS plus SF plus IL-3 plus IL-6 plus Epo) was not sufficient to prevent a rapid and significant loss of DSR activity. In addition, we found that the kinetics of mitogenesis was not altered even when conditions failed to support the survival of >50% of the cells in the first 24 hr in vitro (i.e., in SF plus IL-11). We also used analysis of split doublets to formally document the execution of first divisions that produce two DSR-HSCs under conditions where survival, proliferation, and self-renewal are all well supported. These findings thus represent a major advance over previously reported results with “optimal” cytokine cocktails (SF plus IL-11, SF plus TPO plus Angptl3 plus IGFBP2 plus FGF plus H, SF plus Wnt3A) that we have now shown do not sustain DSR-HSC activity.

Until recently, the speed with which many adult mouse BM HSCs die (in the first 12 hr) when they are incubated under conditions generally used to stimulate their rapid entry into the cell cycle had not been widely appreciated. In retrospect, this finding may account for historic difficulties in obtaining pure mouse HSC populations and the low yields accompanying more promising approaches. Surprisingly little is known about the specific regulation of HSC viability beyond the level of expression of particular genes with identified roles in general cell survival control and evidence of their activation in leukemia (Jordan and Guzman, 2004). A notable exception was an early study suggesting an ability of Bcl-2 to delay HSC apoptosis and synergize with SF to maintain HSC survival (Domen et al., 2000). We did not find evidence of upregulated Bcl-2 in the HSCs treated with UG26 CM, but this is not surprising because Bcl-2 has not been implicated in the physiological control of HSCs. It is thus inviting to speculate that NGF plus Col 1 and UG26 CM may modulate similar downstream pathways to block apoptosis, as suggested by our finding of a differential expression of genes in the “Apoptosis” REACTOME pathway following UG26 CM exposure, and the similar relative decrease in Annexin V staining of ESLAM cells incubated with either of these additives.

### The Effects of Stromal Factors on HSC Self-Renewal In Vitro Are Manifest within the First Cell Cycle and Act to Preserve the HSC Lineage Program as well as Their DSR State

The early death of HSCs appeared complete, even before any of these cells began to divide—consistent with a significant dissociation in the signaling pathways that promote survival and mitogenesis. This inference is further supported by the finding that both UG26 cells and NGF plus Col 1 have potent prosurvival HSC activity in the absence of any mitogenic effect on the HSCs thus “protected.” The maintenance of DSR potential also appears to be regulated independent of the control of this early prosurvival effect on initially isolated quiescent adult HSCs because the FBS plus SF plus IL-3 plus IL-6 plus Epo cocktail was similarly able to prevent early death of HSCs but induced a rapid loss of their self-renewal property. Analysis of paired daughter cells of individual input ESLAM cells further showed that they required early exposure to UG26 CM (in their first cell cycle) to retain a DSR-HSC state. This is an important extension of our previous observation that abrogation of all HSC activity can be obtained even before the cells complete a first cell cycle if they are exposed to suboptimal concentrations of SF (Kent et al., 2008). Together, these results suggest that survival and maintenance of DSR competence in quiescent HSCs depend on their continuous exposure to different external factors that act via pathways (or pathway elements) that may not even require, nor involve, entry into the cell cycle.

Of additional interest is the observation that the time taken for mitogenically stimulated ESLAM cells to complete a first mitosis is positively associated with the likelihood that at least one of their two daughter cells will retain HSC functionality. This is consistent with previous evidence that longer cell-cycle transit times correlate with the most primitive HSCs (Dykstra et al., 2006, Lutolf et al., 2009, Yamazaki et al., 2009). Such associations suggest the possibility that cell-cycle control, like retention of GM differentiation potential, may be mechanistically linked to DSR competence in adult mouse BM HSCs.

Our findings are also potentially relevant to understanding the role of transplantation assays in detecting cells with the molecular machinery required for HSC activity. Single-cell transplantation experiments, confirmed here, have consistently shown that approximately half of these cells are detectable as HSCs, whereas the other half is not. However, as now revealed, nearly all FACS-purified ESLAM cells can display extensive proliferative potential in vitro, even though only half is detectable as LTC-ICs in a 6- to 7-week assay (Kent et al., 2009). Moreover, the frequency of ESLAM cells that can respond to SF plus IL-11 in the presence of UG26 CM in vitro by generating progeny HSCs that are functional in vivo is significantly higher than the 40% of freshly isolated ESLAM cells that are directly detectable in vivo as HSCs. Taken together, this raises the possibility that most adult BM ESLAM cells have not irreversibly lost the molecular status of HSCs.

Overall, our results suggest that several core HSC behavioral programs can be functionally uncoupled, allowing their differential and combinatorial activation by an array of external factors. This differential program activation could result from activation of multiple independent signaling pathways (a combinatorial switch mechanism), by different levels of activation in a few common pathways (a cellular rheostat-like mechanism), or by some combination of these two. In either case, additional downstream molecular interactions are likely. The various genes and pathways previously implicated in HSC maintenance/expansion are consistent with a combinatorial mechanism operating to control HSCs (Reya et al., 2003). Similarly, the fact that low concentrations of SF provide HSC survival benefits, whereas maintenance of repopulation potential requires high levels of SF, supports the existence of mechanisms that depend on different signaling thresholds in these cells (Kent et al., 2008).

### Implications for Future Improvement of HSC Expansion Protocols

Our findings reemphasize the deficiency of 4- to 6-month PB repopulation endpoints that do not specifically measure the output of donor-derived GM cells in order to distinguish between HSCs that have retained or lost DSR activity, as recently highlighted by others (Yamamoto et al., 2013). They also demonstrate that mouse DSR-HSC self-renewal divisions can be achieved under defined conditions in vitro in the absence of any other cells, but to achieve this response, multiple extrinsic factors are required. Interestingly, at least some of these factors, exemplified by Col 1 (Hu et al., 2011) and NGF (García et al., 2004), may be ubiquitously prevalent extracellular matrix components of the interstitial space within hematopoietic tissue. Recent studies have highlighted a differential expression on the surface of HSCs and their closely related downstream derivatives of several integrins, which are receptors for such proteins (Benveniste et al., 2010, Notta et al., 2011, Wagers and Weissman, 2006). Thus, factors that activate these receptors may constitute an additional strategy for enhancing HSC expansion, as suggested by others (Celebi et al., 2011, Kurth et al., 2011, Umemoto et al., 2012). The ability of a combination of defined soluble proteins to promote HSC expansion in vitro refutes the hypothesis that cell contact is required to mediate such responses and should facilitate future interrogation of the mechanisms involved in the maintenance of the DSR state when HSCs are stimulated to proliferate.

## Experimental Procedures

### Mice

C57Bl/6J (B6)-*Ly5.1* or C57Bl/6J (B6)-*Ly5.2* mice and congenic B6-*W41/W41*-*Ly5.1* and B6-*W41/W41*-*Ly5.2* (*W41-5.1* and *W41-5.2*, respectively) mice were bred and maintained in our animal resource center in microisolator cages and provided with continuous sterile food, water, and bedding. Procedures for isolating ESLAM cells from adult mouse BM and performing and assessing transplants were as previously described (Benz et al., 2012, Kent et al., 2009) and carried out with approval from the University of British Columbia Animal Care Committee. For further details, see Supplemental Experimental Procedures.

### UG26 Cells and CM

UG26 stromal cells were cultured as previously described (Oostendorp et al., 2002) and CM obtained from confluent UG26 cells X-irradiated with 30 Gy and then incubated for 3 days with SFM after removal of the UG26 culture medium and rinsing the cultures several times with PBS. The CM was then filtered through a 40 μm cell strainer (Becton Dickinson) and stored frozen at −20°C.

### ESLAM Cell Cultures

ESLAM cells were deposited into the round-bottomed wells of 96-well plates using the single-cell deposition unit of the sorter, each well having been preloaded with 100 μl of SFM (Iscove’s medium with 10 mg/ml BSA, 10 μg/ml insulin, and 200 μg/ml transferrin, 40 μg/ml low-density lipoproteins, 100 U/ml penicillin, 100 μg/ml streptomycin [STEMCELL Technologies]) and 10^−4^ M β-mercaptoethanol (Sigma-Aldrich). The presence of single cells was then confirmed by visual inspection. A second 100 μl of medium was added for cultures initiated with 30 ESLAM cells. The following additives were used as indicated: mouse SF and IL-3, and human Epo and Col 1 purchased from STEMCELL Technologies; human IL-11 and macrophage colony stimulating factor (M-CSF) obtained as gifts from Genetics Institute; mouse Wnt3a, Angptl3, and IGFBP2, and human NGF, pleiotrophin, bone morphogenetic protein 4 (BMP4), Activin A, transforming growth factor β (TGF-β), and platelet-derived growth factor (PDGF) BB purchased from R&D Systems; mouse TPO obtained as a gift from Genentech; human FGF-1 purchased from Invitrogen; heparin, fibronectin, human epidermal growth factor, and mouse laminin purchased from Sigma-Aldrich; human IL-6 obtained as a gift from Cangene; reduced growth factor (RGF) Matrigel purchased from BD; and SDF-1 obtained as a gift from Dr. I. Clark Lewis (University of British Columbia).

For in vivo and LTC-IC assessment of the first division progeny of single ESLAM cells, cultures were examined microscopically every 4 hr starting 32 hr after initiation of the culture. Thereafter, the entire volume in each well found to have produced two cells in the previous 4 hr was distributed into three or more wells to obtain both daughters in different wells so they could then be transplanted separately into two different mice or used to initiate two separate LTCs. If only one cell was recovered, the remaining cell was discarded. To track the kinetics of cell division and viability, cultures were monitored starting 12 hr after initiation and thereafter as indicated. The first appearance of two refractile cells was used to indicate completion of a first division. Following the addition of 15% FBS, 50 ng/ml SF, 10 ng/ml IL-3, 10 ng/ml IL-6, and 3 U/ml Epo to each well after 4½ days of incubation, evidence of viability was inferred from the detection of a clone of seven or more refractile cells. To measure apoptotic cells, cells were harvested after 36 hr, washed, resuspended in Binding Buffer, stained with Annexin V eFluor 450 and FITC-conjugated anti-CD45 (all from eBioscience), and analyzed on a BD LSR Fortessa.

### LTC-IC Assays

Visually confirmed single cells were added onto irradiated UG26 feeder cells in flat-bottomed wells containing 200 μl of MyeloCult (STEMCELL Technologies) supplemented with 10^−6^ M hydrocortisone (Sigma-Aldrich) in 96-well plates and cultures maintained with weekly half-medium changes (Woehrer et al., 2013). After 6 weeks, fresh SFM containing FBS plus SF plus IL-3 plus IL-6 plus Epo was added to the wells, and those containing clusters of >25 nonadherent cells 12 days later were counted as positive.

### Transcriptome Analyses

Between 2,000 (cultured cells) and 6,000 (freshly isolated adult BM) ESLAM cells were collected, and mRNA was extracted with RNeasy (QIAGEN). mRNA from three independent experiments was pooled, reverse transcribed, and amplified with Nanokit following the manufacturer’s instructions (Agilent Technologies). cRNA was hybridized onto two gene chips (GeneChip Mouse Gene 1.0 ST Array; Affymetrix) per condition (GSE57220, http://www.ncbi.nlm.nih.gov/geo/query/acc.cgi?acc=GSE57220). Data analysis was performed as detailed in Supplemental Experimental Procedures.

### Statistical Analysis

GraphPad Prism version 5 or R (http://www.R-project.org/) was used to perform basic statistical analyses, including calculation of mean ± SEM values and to perform Student’s t tests. ELDA: Extreme Limiting Dilution Analysis (http://bioinf.wehi.edu.au/software/elda/) or the “elda” function in the R package, “statmod,” was used to perform the limiting dilution analyses and to evaluate the significance of differences obtained using different culture conditions.

## Author Contributions

S.W., D.J.H.F.K., and C.J.E. conceived and oversaw the design and execution of the experiments. S.W., D.J.H.F.K., M.R.C., C.B., D.G.K., S.B., K.R., and H.M. collected the data. S.W., D.J.H.F.K., M.R.C., C.B., D.G.K., and S.B. contributed to the interpretation of the data. S.W., D.J.H.F.K., and C.J.E. wrote the manuscript, and all authors approved it.

## Figures and Tables

**Table 1:** Serial Tracking of the HSC Subtypes Produced by ESLAM Cells Stimulated to Execute a First HSC Self-Renewal Division In Vitro

First Division HSC Subtypes Detected in Primary Mice	Progeny HSC Subtypes Detected in Secondary Mice
DSR-HSCs (n = 15)	73% (11 out of 15) DSR-HSCs
27% (4 out of 15) LSR-HSCs
LSR-HSCs (n = 7)	0% (0 out of 7) no repopulation

The left column refers to the DSR- and LSR-HSC subtypes identified among the first-division progeny of ESLAM cells generated in vitro as determined from their reconstituting properties in primary recipients (as shown in Figure 4B). The right column refers to the DSR activity seen in secondary recipients of cells transplanted with BM harvested from the primary mice.

## Accession Numbers

The GEO accession number for the transcriptome data reported in this paper is GSE57220.

## Supplemental Information

Document S1. Supplemental Experimental Procedures, Figure S1, and Tables S1–S3

Document S2. Article plus Supplemental Information
