# Supplementary material for: Distinct Stromal Cell Factor Combinations Can Separately Control Hematopoietic Stem Cell Survival, Proliferation, and Self-Renewal
Source: Cell Rep. 2014 Jun 6;7(6):1956–67. doi: 10.1016/j.celrep.2014.05.014 (PMC4074342; doi:10.1016/j.celrep.2014.05.014)
Supplement: Document S1. Supplemental Experimental Procedures, Figure S1, and Tables S1–S3 [file mmc1.pdf]

SUPPLEMENTAL MATERIAL

Supplemental Methods - HSC transplantation assays

Cells were transplanted by tail vein injection into sublethally irradiated Ly5-congenic *W41* mice and PB follow-up analyses of donor contributions to the B, T and GM lineages were then performed 8, 16, and 24 weeks later as previously described (Kent et al., 2007) using both anti–CD45.1-APC (eBiosciences) and anti–CD45.2-FITC (BD) to enable donor and recipient cells to be distinguished and to ensure that any double positive CD45.1 and CD45.2 events were excluded. Staining with anti–Ly6g-PE and anti–Mac1-PE was used to detect GM cells, anti–CD19-PE for B-cells, and anti–CD5-PE for T-cells (all antibodies from BD). Secondary transplantations were performed 24 weeks after the primary transplantations. The subtype of the original HSC(s) transplanted was first determined 16 weeks post transplantation and reconfirmed either within one week before or on the day cells were harvested to perform secondary transplantations. For the latter, BM cells were harvested from both femurs, tibiae and the pelvis of primary mice, red blood cells lysed, and 10^7 cells then injected into each secondary recipient. HSC frequency values were calculated from limiting dilution transplant results using an on-line program (http://bioinf.wehi.edu.au/software/elda/) and a positive read-out of >1% contribution to the total PB white blood cell pool at 16 weeks. HSCs were subclassified as DSR-HSCs when they produced >1% of the PB GM cells and as LSR-HSCs when they contributed <1% of the GM cells 16 weeks after transplantation. Differentiation patterns of HSCs were subclassified as α, β, γ, and δ based on the following ratios of their contributions to the total PB GM vs (T+B)-cell pools in recipient mice assessed 16-24 weeks post-transplant (i.e., >2.0; 0.25-2.0, <0.25 with >1% contribution to the myeloid lineage, and <0.25 with <1% contribution to the myeloid lineage, respectively), as described previously (Dykstra et al., 2007).

Microarray Analysis

All arrays were robust multi-array averaged (RMA) normalized using the Bioconductor package 'xps' in R (http://www.R-project.org/) including the metacore probesets grouped by exon (Gentleman et al., 2004). Gene annotation data was added using a combination of the NetAffx (Release 31) annotation files as well as the Bioconductor packages 'mogene10sttranscriptcluster.db' and 'Org.Mm.eg.db'. All probe sets that were not mapped to an Entrez Gene identifier were discarded. Data Above Background (DAB) scores were calculated for each probe set and those probe sets with a DAB p-value $\leq$0.05 in both technical replicates of at least one condition were retained. In the case of multiple probe sets mapping to the same Entrez Gene identifier, the probe set with the highest maximum absolute deviation across all chips was retained. This was done in order to maximize power in later statistical tests as it reduces multiple testing while retaining the probes with the greatest signal. Expression values were then normalized between the arrays by quantile normalization (Smyth et al., 2005) using the R package 'limma'.

Publicly available microarray data for the UG26-1B6 cell line (Gene Expression Omnibus, GSE11589) was used together with the data for the freshly isolated ESLAM cells (from above) in order to generate a list of potential secreted factors. This data (from the mouse 4302 chip) was RMA-normalized and transcripts called as present if at least one probe set had a MAS5 p-value of 0.05 or less using the R package ‘affy’. Transcripts that were defined as present and that also had the gene ontology (GO) term “extracellular region” (GO:0005576) were compared to transcripts with the gene ontology term “receptor activity” (GO:0004872) present in transcripts of fresh ESLAM cells (as defined above). “Extracellular region” transcripts from UG26 which had transcripts with “receptor activity” in fresh ESLAM cells predicted or

shown to interact in either the National Institute of Aging (NIA) mouse protein-protein interaction database (Yellaboina et al., 2008), the BioGRID database (Stark et al., 2006) or the Cytokine-cytokine receptor interaction (mmu04060) or ECM-receptor interaction (mmu04512) entries from the Kyoto Encyclopedia of Genes and Genomes (KEGG) Pathway database (www.genome.jp/kegg/pathway.html) were retained as potential secreted factors, and their median expression and putative receptors (interacting "receptor activity" transcripts) recorded.

For pathway analysis, arrays from extracts of cells exposed to CM were grouped and compared against extracts from fresh ESLAM cells and from cells exposed to SF+IL-11 only in order to provide biological replication and minimize differences due only to differential cell cycle status. The 'romer' algorithm (a rotation based modification of the GSEA algorithm (Subramanian et al., 2005)) was used to test for differential expression of genes in pathways from the REACTOME database (Croft et al.) (taken from the R version of the molecular signatures database, http://bioinf.wehi.edu.au/software/MSigDB/) using a mixed alternative hypothesis (i.e., the differential expression can be in both directions) with the "floor mean" test statistic and 100,000 rotations, correcting for correlation between technical replicates (Smyth et al., 2005).

Supplemental Figure

Figure S1. Comparison of the serial GM-, B-, and T-cell reconstituting activities of the 2 β-HSCs and their progeny produced from the first *in vitro* division of a single ESLAM cell. Donor-derived contributions to the PB GM cells are shown in red, to the PB B-cells in blue, and to the PB T-cells in black.

Supplementary Figure 1

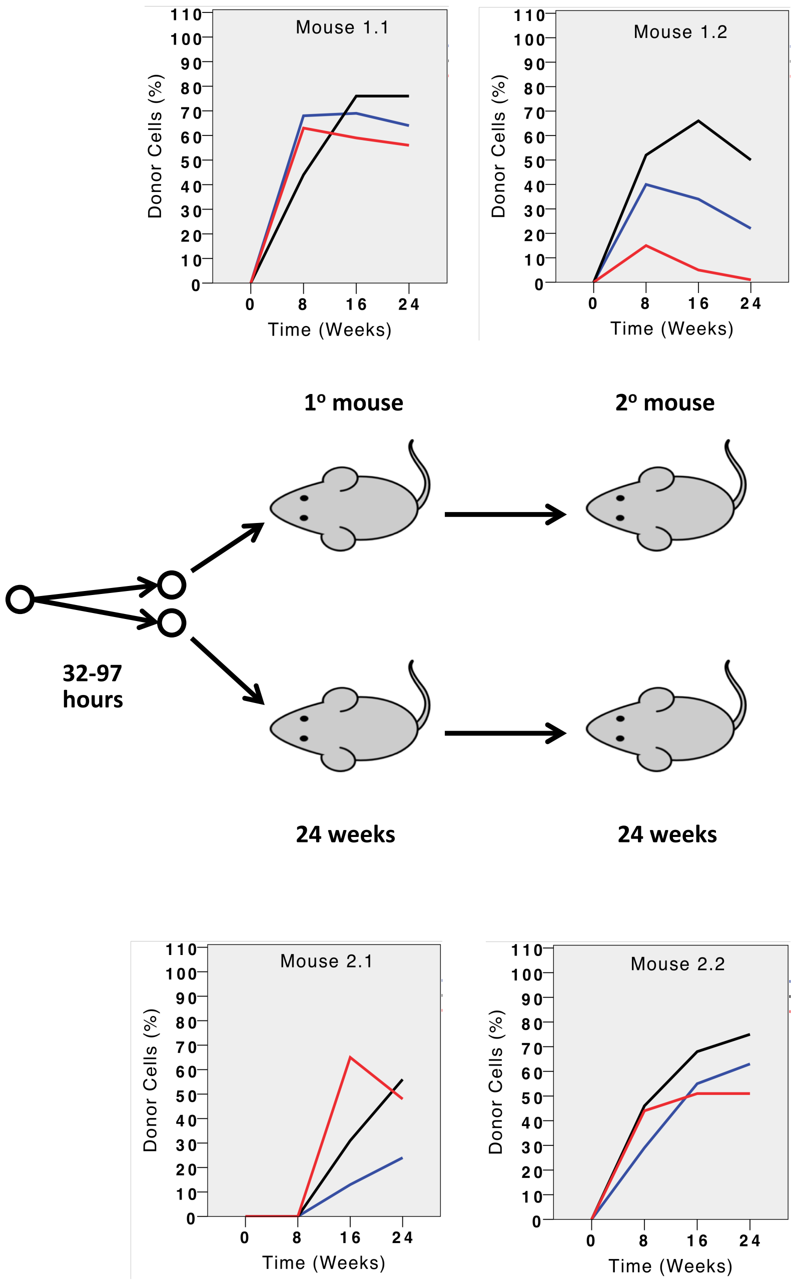

Supplemental Tables

Table S1: REACTOME pathways (n=250) with significantly altered transcript expression in ESLAM cells maintained in S+11+UG26 CM.

**Pathway**	**Gene No.**	**P value**
ADP SIGNALLING THROUGH P2Y PURINOCEPTOR 1	25	9.9999E-06
APOPTOSIS	122	9.9999E-06
AUTODEGRADATION OF CDH1 BY CDH1 APC	53	9.9999E-06
AXON GUIDANCE	156	9.9999E-06
BRANCHED CHAIN AMINO ACID CATABOLISM	17	9.9999E-06
CD28 DEPENDENT VAV1 PATHWAY	11	9.9999E-06
CDC20 PHOSPHO APC MEDIATED DEGRADATION OF CYCLIN A	59	9.9999E-06
CDT1 ASSOCIATION WITH THE CDC6 ORC ORIGIN COMPLEX	48	9.9999E-06
CELL CYCLE CHECKPOINTS	104	9.9999E-06
CELL CYCLE MITOTIC	292	9.9999E-06
CELL SURFACE INTERACTIONS AT THE VASCULAR WALL	89	9.9999E-06
CENTROSOME MATURATION	67	9.9999E-06
CITRIC ACID CYCLE	18	9.9999E-06
CLATHRIN DERIVED VESICLE BUDDING	56	9.9999E-06
COSTIMULATION BY THE CD28 FAMILY	60	9.9999E-06
CYCLIN E ASSOCIATED EVENTS DURING G1 S TRANSITION	54	9.9999E-06
DARPP32 EVENTS	25	9.9999E-06
DIABETES PATHWAYS	292	9.9999E-06
DNA REPAIR	98	9.9999E-06
DNA REPLICATION PRE INITIATION	71	9.9999E-06
DOUBLE STRAND BREAK REPAIR	21	9.9999E-06
DOWN STREAM SIGNAL TRANSDUCTION	35	9.9999E-06
DOWNSTREAM TCR SIGNALING	36	9.9999E-06
ELECTRON TRANSPORT CHAIN	56	9.9999E-06
ELONGATION AND PROCESSING OF CAPPED TRANSCRIPTS	109	9.9999E-06
FORMATION AND MATURATION OF MRNA TRANSCRIPT	127	9.9999E-06
FORMATION OF A POOL OF FREE 40S SUBUNITS	40	9.9999E-06
FORMATION OF ATP BY CHEMIOSMOTIC COUPLING	10	9.9999E-06
FORMATION OF PLATELET PLUG	180	9.9999E-06
FORMATION OF THE TERNARY COMPLEX AND SUBSEQUENTLY THE 43S COMPLEX	27	9.9999E-06
FURTHER PLATELET RELEASATE	22	9.9999E-06
G1 S TRANSITION	95	9.9999E-06
G2 M TRANSITION	79	9.9999E-06
GENE EXPRESSION	341	9.9999E-06
GENERIC TRANSCRIPTION PATHWAY	35	9.9999E-06
GLOBAL GENOMIC NER	32	9.9999E-06
GLUCONEOGENESIS	27	9.9999E-06
GLUCOSE METABOLISM	51	9.9999E-06
GLUCOSE REGULATION OF INSULIN SECRETION	130	9.9999E-06

GLYCOGEN BREAKDOWN GLYCOGENOLYSIS	15	9.9999E-06
GLYCOLYSIS	19	9.9999E-06
GOLGI ASSOCIATED VESICLE BIOGENESIS	50	9.9999E-06
GRB2 SOS PROVIDES LINKAGE TO MAPK SIGNALING FOR INTERGRINS	15	9.9999E-06
GTP HYDROLYSIS AND JOINING OF THE 60S RIBOSOMAL SUBUNIT	50	9.9999E-06
HEMOSTASIS	264	9.9999E-06
HIV INFECTION	169	9.9999E-06
HIV LIFE CYCLE	98	9.9999E-06
HIV1 TRANSCRIPTION INITIATION	38	9.9999E-06
HOST INTERACTIONS OF HIV FACTORS	109	9.9999E-06
INFLUENZA LIFE CYCLE	80	9.9999E-06
INFLUENZA VIRAL RNA TRANSCRIPTION AND REPLICATION	44	9.9999E-06
INSULIN SYNTHESIS AND SECRETION	72	9.9999E-06
INTEGRATION OF ENERGY METABOLISM	198	9.9999E-06
INTEGRIN CELL SURFACE INTERACTIONS	80	9.9999E-06
LATE PHASE OF HIV LIFE CYCLE	87	9.9999E-06
LOSS OF NLP FROM MITOTIC CENTROSOMES	60	9.9999E-06
M G1 TRANSITION	57	9.9999E-06
MEMBRANE TRAFFICKING	73	9.9999E-06
METABLISM OF NUCLEOTIDES	70	9.9999E-06
METABOLISM OF CARBOHYDRATES	109	9.9999E-06
METABOLISM OF PROTEINS	153	9.9999E-06
MITOTIC PROMETAPHASE	89	9.9999E-06
MRNA 3 END PROCESSING	22	9.9999E-06
MRNA PROCESSING	31	9.9999E-06
MRNA SPLICING	83	9.9999E-06
MRNA SPLICING MINOR PATHWAY	32	9.9999E-06
NCAM SIGNALING FOR NEURITE OUT GROWTH	67	9.9999E-06
NCAM1 INTERACTIONS	43	9.9999E-06
NUCLEAR IMPORT OF REV PROTEIN	29	9.9999E-06
NUCLEOTIDE EXCISION REPAIR	47	9.9999E-06
OPIOID SIGNALLING	82	9.9999E-06
ORC1 REMOVAL FROM CHROMATIN	59	9.9999E-06
OTHER SEMAPHORIN INTERACTIONS	15	9.9999E-06
P38MAPK EVENTS	13	9.9999E-06
P53 INDEPENDENT DNA DAMAGE RESPONSE	39	9.9999E-06
PEPTIDE CHAIN ELONGATION	31	9.9999E-06
PLATELET ACTIVATION	161	9.9999E-06
PLATELET ACTIVATION TRIGGERS	58	9.9999E-06
PLATELET DEGRANULATION	82	9.9999E-06
PREFOLDIN MEDIATED TRANSFER OF SUBSTRATE TO CCT TRIC	24	9.9999E-06
PROCESSING OF CAPPED INTRON CONTAINING PRE MRNA	113	9.9999E-06
PYRUVATE METABOLISM AND TCA CYCLE	35	9.9999E-06
REGULATION OF APC ACTIVATORS BETWEEN G1 S AND EARLY ANAPHASE	66	9.9999E-06
REGULATION OF BETA CELL DEVELOPMENT	62	9.9999E-06
REGULATION OF GENE EXPRESSION IN BETA CELLS	48	9.9999E-06
REGULATION OF INSULIN SECRETION	180	9.9999E-06
REGULATION OF INSULIN SECRETION BY GLUCAGON LIKE PEPTIDE 1	59	9.9999E-06
REGULATION OF ORNITHINE DECARBOXYLASE	43	9.9999E-06
REV MEDIATED NUCLEAR EXPORT OF HIV1 RNA	30	9.9999E-06

RHO GTPASE CYCLE	120	9.9999E-06
RNA POL II CTD PHOSPHORYLATION AND INTERACTION WITH CE	25	9.9999E-06
RNA POLYMERASE I CHAIN ELONGATION	20	9.9999E-06
RNA POLYMERASE I PROMOTER ESCAPE	20	9.9999E-06
RNA POLYMERASE I TRANSCRIPTION INITIATION	24	9.9999E-06
RNA POLYMERASE I TRANSCRIPTION TERMINATION	21	9.9999E-06
RNA POLYMERASE II TRANSCRIPTION	78	9.9999E-06
S PHASE	98	9.9999E-06
SCF BETA TRCP MEDIATED DEGRADATION OF EMI1	44	9.9999E-06
SCF SKP2 MEDIATED DEGRADATION OF P27 P21	48	9.9999E-06
SEMA3A PAK DEPENDENT AXON REPULSION	13	9.9999E-06
SEMA4D IN SEMAPHORIN SIGNALING	28	9.9999E-06
SEMA4D INDUCED CELL MIGRATION AND GROWTH CONE COLLAPSE	23	9.9999E-06
SEMAPHORIN INTERACTIONS	63	9.9999E-06
SHC MEDIATED SIGNALLING	12	9.9999E-06
SIGNALING BY PDGF	63	9.9999E-06
SIGNALING BY TGF BETA	15	9.9999E-06
SIGNALING BY WNT	54	9.9999E-06
SIGNALING IN IMMUNE SYSTEM	294	9.9999E-06
SIGNALLING BY NGF	208	9.9999E-06
SIGNALLING TO ERKS	34	9.9999E-06
SIGNALLING TO RAS	26	9.9999E-06
SNRNP ASSEMBLY	45	9.9999E-06
SOS MEDIATED SIGNALLING	13	9.9999E-06
SYNTHESIS OF DNA	84	9.9999E-06
TCR SIGNALING	53	9.9999E-06
TIE2 SIGNALING	18	9.9999E-06
TRAF6 MEDIATED INDUCTION OF THE ANTIVIRAL CYTOKINE IFN ALPHA BETA CASCADE	52	9.9999E-06
TRANSCRIPTION	146	9.9999E-06
TRANSCRIPTION OF THE HIV GENOME	57	9.9999E-06
TRANSLATION	64	9.9999E-06
TRANSLATION INITIATION COMPLEX FORMATION	33	9.9999E-06
TRANSMISSION ACROSS CHEMICAL SYNAPSES	127	9.9999E-06
TRANSPORT OF MATURE MRNA DERIVED FROM AN INTRON CONTAINING TRANSCRIPT	39	9.9999E-06
TRKA SIGNALLING FROM THE PLASMA MEMBRANE	102	9.9999E-06
VIF MEDIATED DEGRADATION OF APOBEC3G	42	9.9999E-06
VIRAL MRNA TRANSLATION	30	9.9999E-06
VPR MEDIATED NUCLEAR IMPORT OF PICS	30	9.9999E-06
PP2A MEDIATED DEPHOSPHORYLATION OF KEY METABOLIC FACTORS	10	9.9999E-06
SYNTHESIS AND INTERCONVERSION OF NUCLEOTIDE DI AND TRIPHOSPHATES	18	9.9999E-06
TRANSPORT OF RIBONUCLEOPROTEINS INTO THE HOST NUCLEUS	28	9.9999E-06
GLUCOSE TRANSPORT	37	9.9999E-06
MRNA DECAY BY 5 TO 3 EXORIBONUCLEASE	12	9.9999E-06
REGULATION OF GLUCOKINASE BY GLUCOKINASE REGULATORY PROTEIN	28	9.9999E-06
STABILIZATION OF P53	41	9.9999E-06
DEPOLARIZATION OF THE PRESYNAPTIC TERMINAL TRIGGERS THE	12	9.9999E-06

OPENING OF CALCIUM CHANNELS		
INHIBITION OF INSULIN SECRETION BY ADRENALINE NORADRENALINE	30	9.9999E-06
MRNA DECAY BY 3 TO 5 EXORIBONUCLEASE	10	9.9999E-06
NEP NS2 INTERACTS WITH THE CELLULAR EXPORT MACHINERY	28	9.9999E-06
RNA POLYMERASE I III AND MITOCHONDRIAL TRANSCRIPTION	82	9.9999E-06
TRANSPORT OF THE SLBP INDEPENDENT MATURE MRNA	31	9.9999E-06
SMOOTH MUSCLE CONTRACTION	23	9.9999E-06
METABOLISM OF MRNA	42	9.9999E-06
METABOLISM OF RNA	87	9.9999E-06
MITOTIC M M G1 PHASES	150	9.9999E-06
APCDC20 MEDIATED DEGRADATION OF CYCLIN B	16	1.99998E-05
CHAPERONIN MEDIATED PROTEIN FOLDING	45	1.99998E-05
FORMATION OF TUBULIN FOLDING INTERMEDIATES BY CCT TRIC	17	1.99998E-05
INACTIVATION OF APC VIA DIRECT INHIBITION OF THE APCOMPLEX	18	1.99998E-05
GRB2 EVENTS IN EGFR SIGNALING	13	2.99997E-05
HIV1 TRANSCRIPTION ELONGATION	39	2.99997E-05
P75 NTR RECEPTOR MEDIATED SIGNALLING	78	2.99997E-05
RNA POLYMERASE I PROMOTER CLEARANCE	49	2.99997E-05
SEMA3A PLEXIN REPULSION SIGNALING BY INHIBITING INTEGRIN ADHESION	13	2.99997E-05
DUAL INCISION REACTION IN GG NER	20	3.99996E-05
SIGNAL AMPLIFICATION	31	5.99994E-05
LYSOSOME VESICLE BIOGENESIS	22	6.99993E-05
MTOR SIGNALLING	26	6.99993E-05
MUSCLE CONTRACTION	50	8.99991E-05
MAP KINASES ACTIVATION IN TLR CASCADE	44	0.000109999
METABOLISM OF AMINO ACIDS	152	0.000119999
TRANSCRIPTION COUPLED NER	42	0.000129999
PHOSPHORYLATION OF THE APC	16	0.000149999
DUAL INCISION REACTION IN TC NER	27	0.000159998
RNA POLYMERASE III TRANSCRIPTION TERMINATION	16	0.000159998
TOLL RECEPTOR CASCADES	83	0.000209998
G ALPHA 12 13 SIGNALLING EVENTS	54	0.000259997
HORMONE BIOSYNTHESIS	48	0.000289997
TOLL LIKE RECEPTOR 3 CASCADE	58	0.000339997
EXTENSION OF TELOMERES	26	0.000369996
CONVERSION FROM APC CDC20 TO APC CDH1 IN LATE ANAPHASE	17	0.000419996
CYCLIN A1 ASSOCIATED EVENTS DURING G2 M TRANSITION	14	0.000419996
MYOGENESSIS	28	0.000419996
CRMPS IN SEMA3A SIGNALING	15	0.000689993
ENERGY DEPENDENT REGULATION OF MTOR BY LKB1 AMPK	17	0.000689993
PURINE RIBONUCLEOSIDE MONOPHOSPHATE BIOSYNTHESIS	11	0.000729993
REGULATION OF AMPK ACTIVITY VIA LKB1	14	0.000949991
SIGNALLING TO P38 VIA RIT AND RIN	14	0.00099999
SHC RELATED EVENTS	14	0.00104999
ACTIVATION OF ATR IN RESPONSE TO REPLICATION STRESS	37	0.001199988
NEURORANSMITTER RECEPTOR BINDING AND DOWNSTREAM TRANSMISSION IN THE POSTSYNAPTIC CELL	81	0.001329987
P130CAS LINKAGE TO MAPK SIGNALING FOR INTEGRINS	15	0.001429986
IRS RELATED EVENTS	78	0.001439986

COLLAGEN MEDIATED ACTIVATION CASCADE	22	0.001879981
PD1 SIGNALING	19	0.002419976
DNA STRAND ELONGATION	30	0.002529975
ACTIVATION OF NMDA RECEPTOR UPON GLUTAMATE BINDING AND POSTSYNAPTIC EVENTS	35	0.002779972
POST NMDA RECEPTOR ACTIVATION EVENTS	31	0.00297997
ACTIVATION OF KAINATE RECEPTORS UPON GLUTAMATE BINDING	32	0.003079969
UNFOLDED PROTEIN RESPONSE	18	0.003359966
COPI MEDIATED TRANSPORT	10	0.003579964
IONOTROPIC ACTIVITY OF KAINATE RECEPTORS	12	0.003679963
G BETA GAMMA SIGNALLING THROUGH PI3KGAMMA	25	0.004239958
ASSOCIATION OF TRIC CCT WITH TARGET PROTEINS DURING BIOSYNTHESIS	29	0.004539955
FRS2 MEDIATED ACTIVATION	16	0.004679953
SIGNALING BY EGFR	46	0.004809952
MAPK TARGETS NUCLEAR EVENTS MEDIATED BY MAP KINASES	30	0.005239948
FANCONI ANEMIA PATHWAY	14	0.005269947
NOREPINEPHRINE NEUROTRANSMITTER RELEASE CYCLE	12	0.005859941
UNWINDING OF DNA	11	0.006569934
G PROTEIN ACTIVATION	28	0.007599924
HOMOLOGOUS RECOMBINATION REPAIR	15	0.007729923
POST CHAPERONIN TUBULIN FOLDING PATHWAY	14	0.007929921
INNATE IMMUNITY SIGNALING	102	0.008489915
FORMATION OF THE EARLY ELONGATION COMPLEX	30	0.008569914
ERKS ARE INACTIVATED	12	0.008609914
G PROTEIN BETA GAMMA SIGNALLING	28	0.00895991
THROMBOXANE SIGNALLING THROUGH TP RECEPTOR	23	0.009259907
CYTOSOLIC TRNA AMINOACYLATION	23	0.009319907
THROMBIN SIGNALLING THROUGH PROTEINASE ACTIVATED RECEPTORS	27	0.009469905
ACTIVATION OF THE PRE REPLICATIVE COMPLEX	29	0.009619904
SIGNALING BY VEGF	11	0.009619904
INTEGRIN ALPHAIIBBETA3 SIGNALING	23	0.009699903
CAM PATHWAY	25	0.009899901
POST TRANSLATIONAL PROTEIN MODIFICATION	39	0.009939901
CELL DEATH SIGNALLING VIA NRAGE NRIF AND NADE	58	0.0100299
NOTCH HLH TRANSCRIPTION PATHWAY	13	0.010729893
PLC BETA MEDIATED EVENTS	37	0.011629884
G1 PHASE	16	0.012059879
ACTIVATION OF THE AP1 FAMILY OF TRANSCRIPTION FACTORS	10	0.012179878
NUCLEAR EVENTS KINASE AND TRANSCRIPTION FACTOR ACTIVATION	24	0.014819852
CD28 CO STIMULATION	29	0.01495985
RECRUITMENT OF NUMA TO MITOTIC CENTROSOMES	9	0.015909841
INTRINSIC PATHWAY FOR APOPTOSIS	28	0.016219838
EARLY PHASE OF HIV LIFE CYCLE	11	0.016759832
REGULATION OF LIPID METABOLISM BY PEROXISOME PROLIFERATOR ACTIVATED RECEPTOR ALPHA	59	0.017239828
METABOLISM OF NITRIC OXIDE	12	0.017549825
ACTIVATED AMPK STIMULATES FATTY ACID OXIDATION IN MUSCLE	17	0.018879811
STEROID HORMONE BIOSYNTHESIS	12	0.020779792

GLUCAGON TYPE LIGAND RECEPTORS	33	0.020829792
G2 M CHECKPOINTS	42	0.021099789
PURINE METABOLISM	30	0.023589764
RNA POLYMERASE III TRANSCRIPTION	32	0.024209758
PLC GAMMA1 SIGNALLING	34	0.024369756
RNA POLYMERASE III TRANSCRIPTION INITIATION FROM TYPE 3 PROMOTER	21	0.025379746
EGFR DOWNREGULATION	22	0.026479735
NRAGE SIGNALS DEATH THROUGH JNK	47	0.026749733
ERK MAPK TARGETS	21	0.02903971
TAT MEDIATED HIV1 ELONGATION ARREST AND RECOVERY	28	0.030679693
ADP SIGNALLING THROUGH P2Y PURINOCEPTOR 12	21	0.030729693
STEROID HORMONES	19	0.030919691
PI3K AKT SIGNALLING	37	0.031249688
G BETA GAMMA SIGNALLING THROUGH PLC BETA	20	0.031289687
CTLA4 INHIBITORY SIGNALING	21	0.035129649
ACTIVATION OF RAC	14	0.035659643
RAS ACTIVATION UOPN CA2+ INFUX THROUGH NMDA RECEPTOR	17	0.036129639
ACTIVATION OF CHAPERONES BY IRE1 ALPHA	9	0.042199578
POLYMERASE SWITCHING	13	0.042259577
MTORC1 MEDIATED SIGNALLING	10	0.044689553
CD28 DEPENDENT PI3K AKT SIGNALING	19	0.046469535
RNA POLYMERASE III CHAIN ELONGATION	11	0.047679523

Table S2. Significantly affected pathways of ESLAM cells upon stimulation with UG26 CM and SF+IL-11.

Signaling pathways	Number of involved genes	Regulation of HSC	References
NGF	208	AGM HSC activity	(Durand et al., 2007)
PDGF	63	HSC expansion	(Su et al., 2002)
WNT	54	HSC proliferation	(Reya et al., 2003; Willert et al., 2003)
EGFR	46	HSC migration	(Ryan et al.)
ROBO Receptor	32	HSC-niche interaction	(Shibata et al., 2009; Smith-Berdan et al.)
BMP	23	AGM HSC activity	(Durand et al., 2007)
Integrin Signaling	23	HSC adhesion	(Benveniste et al., 2010; Notta et al.)
TGF-ß	15	HSC subtype regulation	(Challen et al.)
Notch	14	HSC *de novo* generation	(Kumano et al., 2003)
VEGF	11	HSC survival	(Gerber et al., 2002)

NGF: nerve growth factor, AGM: aorta-gonad-metanephron, PDGF: platelet derived growth factor, EGFR: epidermal growth factor receptor, ROBO: roundabout family, BMP: bone morphogenic protein, TGF-ß: transforming growth factor-beta, VEGF: vascular endothelial growth factor.

Table S3: Secreted factor mRNAs (n=172) produced by UG26 cells with predicted interactions with the products of genes expressed by activated ESLAM cells.

Entrez Gene ID	Gene Symbol	Mean Intensity (Log_2)	Receptor(s)
16785	Rpsa	13.23	Atp5b, Csf2ra, Itga6, Kdelr1
16852	Lgals1	13.20	Cd7, Lgals3bp, Susd2
16952	Anxa1	12.77	Fpr1, Trpm7
15481	Hspa8	12.59	Grb2, Irs1, Cxcr4, Ripk2,Ncor1,Tnfrsf1a, Tnfrsf1b, Cd40, Traf1, Traf2, Atp9b, Ripk3, Kdelr1, Myd88
16854	Lgals3	12.42	Lgals3bp, Cubn, Ncoa3
14683	Gnas	12.32	Grin2b
23980	Pebp1	12.29	Adrbk1, Ppard, Nr2c2
17319	Mif	12.22	Cd74, Tnfrsf14
14456	Gas6	12.18	Mertk, Tyro3
12261	C1qbp	12.14	Gab1, Gabrb1, Tnfrsf1a, Tnfrsf1b, Traf1, Ripk3
22166	Txn1	12.12	Nr3c1
12631	Cfl1	12.11	Grb2
15519	Hsp90aa1	12.03	Alk, Ahr, Aip, Ar, Asgr1, Arntl, Esr1, Nr3c1, Nr3c2, Kdr, Ppara, Tnfrsf1a, Nr2c2, Traf1, Traf2, Ripk3, Ripk2
227753	Gsn	11.86	Ar, Grb2, Grin2b
12306	Anxa2	11.85	Grb2
14219	Ctgf	11.85	Erbb4, Lrp1, Itga5
14115	Fbln2	11.77	Itgb3, Nsd1
18787	Serpine1	11.74	Lrp2, Lrp1, Thbd, Vtn, Lrp1b
21858	Timp2	11.70	Itga3, Pgrmc1
18073	Nid1	11.55	Itgav, Itgb3, Lgals3bp, Ptprf, Notch1
12010	B2m	11.45	Fcgrt, Tfrc
19156	Psap	11.37	Celsr1
21825	Thbs1	11.01	Cd36, Scarb2, Lrp1, Lrp5, Itgb3, Tnfrsf11b
12317	Calr	10.96	Ar, Nr3c1, Itga2b, Itga3, Itgav, Lrp1
12847	Copa	10.88	Pdgfrb, Mtnr1b
20315	Cxcl12	10.78	Cxcr4, Cxcr7, Dpp4
12827	Col4a2	10.78	Cd44, Cd93, Antxr2
21859	Timp3	10.69	Kdr
12826	Col4a1	10.68	Cd44, Cd93
14828	Hspa5	10.65	Atp5b, Scarb2, Htr3a, Ldlr, Grb2, Gria1, Tnfrsf1a, Tnfrsf1b, Traf2, Mtnr1b, Ripk3, Myd88
12833	Col6a1	10.61	Cd44, Lgals3bp
20296	Ccl2	10.52	Ccr2
12842	Col1a1	10.47	Cd44, Cd36, Itga2, Itga5, Cd93, Ddr2
231887	Pdap1	10.39	Pdgfrb
13024	Ctla2a	10.29	Tinagl1
16423	Cd47	10.29	Itgav, P2ry2
14751	Gpi1	10.25	Amfr
12331	Cap1	10.18	Traf3
13722	Aimp1	10.17	Slc20a1

17387	Mmp14	10.13	Lrp1, Itgav
12832	Col5a2	10.06	Cd44
14205	Figf	9.97	Kdr, Flt4, Itga9
13003	Vcan	9.97	Itga4
21814	Tgfbr3	9.96	Acvr2a, Tgfbr2, Tgfbr1
56348	Hsd17b12	9.93	Slc7a1
14268	Fn1	9.91	Cd44
14313	Fst	9.85	Spsb1
16007	Cyr61	9.74	Itgav
12977	Csf1	9.67	Csf1r, Celsr3, Slc7a1, Myd88
20306	Ccl7	9.63	Ccr2, Ccr1, Ccr1l1
226519	Lamc1	9.47	Cd44, Sv2a, Sv2b, Sv2c
14825	Cxcl1	9.31	Cxcr2
21923	Tnc	9.28	Itga9, Itga5, Itgb6, Ptprb, Itga8
12843	Col1a2	9.26	Cd44, Cd36, Itga2, Itga2b, Itgb3, Cd93
57914	Crlf2	9.23	Il7r
19242	Ptn	9.23	Alk, Gnb2l1, Ptprb, Ptprz1, Ryr1
22341	Vegfc	9.20	Kdr, Flt4
14423	Galnt1	9.18	Ptprf
21826	Thbs2	9.13	Cd36
15530	Hspg2	9.08	Itga2
16779	Lamb2	9.00	Cd44, Sv2a, Sv2b, Sv2c
20377	Sfrp1	8.92	Fzd6
12834	Col6a2	8.87	Cd44
16323	Inhba	8.76	Acvr2a, Acvr2b, Acvr1, Tgfbr3
12830	Col4a5	8.75	Cd44, Cd93
53381	Prdx4	8.71	Atp5b
19039	Lgals3bp	8.65	Phb2
54635	Pdgfc	8.63	Pdgfra
12159	Bmp4	8.59	Bmpr1a, Bmpr1b, Bmpr2
16412	Itgb1	8.56	Ptch2
76737	Creld2	8.54	Chrna4, Chrnb2
22340	Vegfb	8.50	Flt1, Nrp1
110611	Hdlbp	8.49	Gnb2l1, Ptch2
12831	Col5a1	8.42	Cd44, Lgals3bp
20348	Sema3c	8.41	Nrp1
19128	Pros1	8.41	Tyro3
18590	Pdgfa	8.40	Pdgfra
16777	Lamb1	8.39	Cd44, Sv2a, Sv2b, Sv2c
14178	Fgf7	8.38	Fgfr3, Fgfr4, Nrp1
13138	Dag1	8.23	Grb2, Rapsn, Musk
11883	Arsa	8.23	Bmpr2
12931	Crlf1	8.22	Cntfr
13874	Ereg	8.19	Erbb4
14600	Ghr	8.14	Grb2, Irs1, Ncoa6
18208	Ntn1	8.09	Adora2b, Dcc, Neo1, Unc5c
56213	Htra1	7.98	Grb2
17311	Kitl	7.89	Kit
21803	Tgfb1	7.85	Acvrl1, Itgav, Itgb6, Tgfbr1, Tgfbr2, Tgfbr3, Vtn
16956	Lpl	7.85	Lrp2, Lrp1
13614	Edn1	7.85	Ednra, Ednrb

17295	Met	7.81	Grb2, Gab1, Ptprb, Ptprj, Spsb1, Itgb4, Plxnb1
11535	Adm	7.76	Calcrl, Gpr182
12837	Col8a1	7.71	Itga1, Itga2, Efemp2
22418	Wnt5a	7.70	Fzd1, Fzd5, Ror2, Lrp6, Ryk
13038	Ctsk	7.70	Fgfr3
15200	Hbegf	7.63	Erbb4
17388	Mmp15	7.53	Lrp1
12064	Bdnf	7.52	Esr1, Sort1
114249	Npnt	7.49	Itga8
16835	Ldlr	7.40	Ldlrap1, Lrpap1, Flt1
12825	Col3a1	7.36	Cd44, Ddr2
433375	Creg1	7.33	Igf2r
11486	Ada	7.27	Adora1, Adora2a, Adora2b, Dpp4, Drd1a, Grb2, Nr3c1
20563	Slit2	7.26	Robo2
16880	Lifr	7.23	Cntfr, Il31ra
12822	Col18a1	7.23	Kdr, Itga5
20564	Slit3	7.17	Robo2
30878	Apln	7.14	Aplnr
15925	Ide	7.13	Ar, Nr3c1
100952	Emilin1	7.11	Tgfbr2
16194	Il6ra	7.00	Erap1
16403	Itga6	6.99	Cd36, Grb2, Itgb4
19206	Ptch1	6.97	Smo
11826	Aqp1	6.94	Trip6, Efemp2
67573	Loxl4	6.89	Trip13
21827	Thbs3	6.88	Cd36
12475	Cd14	6.87	Tlr3, Itgb2, Lgals3bp, Tlr4, Tlr2, Itgam
20210	Saa3	6.71	Fpr1
20350	Sema3f	6.68	Nrp1
11491	Adam17	6.66	Erbb4, Notch1, Ptpn3
94216	Col4a6	6.65	Cd44, Cd93
16190	Il4ra	6.64	Gnb2l1, Il13ra1, Irs1, Irs2, Cd40
18049	Ngf	6.62	Sort1, Ngfr, Ntrk1
20300	Ccl25	6.59	Ccr9, Ccr10, Ccbp2
22417	Wnt4	6.57	Fzd6
56708	Clcf1	6.54	Cntfr, Crlf1
21808	Tgfb2	6.48	Tgfbr1, Tgfbr2, Tgfbr3, Vtn
21802	Tgfa	6.37	Erbb4, Rhbdf1
12835	Col6a3	6.30	Cd44
20312	Cx3cl1	6.28	Cx3cr1
14172	Fgf18	6.23	Fgfr3, Fgfr4
20349	Sema3e	6.21	Plxnd1
13848	Ephb6	6.19	Grb2, Ephb1
22339	Vegfa	6.14	Flt1, Kdr, Grin2b
16173	Il18	6.14	Il1rl2, Il18rap, Il18r1
16819	Lcn2	6.11	Lrp2
53623	Gria3	6.10	Gria2
22403	Wisp2	6.07	Igf1r, Igf2r
22413	Wnt2	6.04	Fzd1, Fzd9
17087	Ly96	6.02	Tlr4, Tlr2
20310	Cxcl2	5.95	Cxcr2

20440	St6gal1	5.94	Cd22
21809	Tgfb3	5.89	Acvrl1, Tgfbr1, Tgfbr2, Tgfbr3
18133	Nov	5.84	Notch1
22042	Tfrc	5.79	Gabarap
16975	Lrp8	5.75	Grin1
20660	Sorl1	5.67	Lrpap1
11815	Apod	5.63	Lepr, Atp5b, Scarb2
14566	Gdf9	5.40	Acvr2a, Bmpr1a, Bmpr1b, Bmpr2
21950	Tnfsf9	5.24	Tnfrsf9, Traf1, Traf2
13214	Defb1	5.23	Ccr6
19143	St14	5.19	F2rl1
16193	Il6	5.14	Il6ra, Hrh1
17082	Il1rl1	5.13	Myd88
13636	Efna1	5.10	Epha1, Epha2, Epha3, Epha4, Epha6, Epha7, Epha8, Ephb1, Tgfbr1
53867	Col5a3	4.91	Cd44
16180	Il1rap	4.88	Irak1, Il1r1
16878	Lif	4.85	Lifr
20750	Spp1	4.68	Itga9, Itga5, Itgav
16196	Il7	4.60	Il7r
18591	Pdgfb	4.31	Pdgfra, Pdgfrb
16169	Il15ra	4.30	Traf2, Il2rb
16000	Igf1	4.26	Igf1r, Igsf1
53603	Tslp	4.05	Il7r, Crlf2
11600	Angpt1	3.90	Itga5, Tek
12223	Btc	3.90	Erbb4, Egfr
11602	Angpt4	3.76	Tek
71785	Pdgfd	3.38	Pdgfrb
53604	Zpbp	3.34	Zp2
110312	Pmch	3.24	Mchr1
12156	Bmp2	3.22	Acvr1, Bmpr1b, Bmpr2, Bmpr1a
13645	Egf	3.19	Egfr, Erbb3, Grb2, Vtn

References:

Benveniste, P., Frelin, C., Janmohamed, S., Barbara, M., Herrington, R., Hyam, D., and Iscove, N.N. (2010). Intermediate-term hematopoietic stem cells with extended but time-limited reconstitution potential. Cell Stem Cell *6*, 48-58.

Challen, G.A., Boles, N.C., Chambers, S.M., and Goodell, M.A. Distinct hematopoietic stem cell subtypes are differentially regulated by TGF-beta1. Cell Stem Cell *6*, 265-278.

Croft, D., O'Kelly, G., Wu, G., Haw, R., Gillespie, M., Matthews, L., Caudy, M., Garapati, P., Gopinath, G., Jassal, B., *et al.* Reactome: a database of reactions, pathways and biological processes. Nucleic Acids Res *39*, D691-697.

Durand, C., Robin, C., Bollerot, K., Baron, M.H., Ottersbach, K., and Dzierzak, E. (2007). Embryonic stromal clones reveal developmental regulators of definitive hematopoietic stem cells. Proc Natl Acad Sci U S A *104*, 20838-20843.

Dykstra, B., Kent, D., Bowie, M., McCaffrey, L., Hamilton, M., Lyons, K., Lee, S.J., Brinkman, R., and Eaves, C. (2007). Long-term propagation of distinct hematopoietic differentiation programs in vivo. Cell Stem Cell *1*, 218-229.

Gentleman, R.C., Carey, V.J., Bates, D.M., Bolstad, B., Dettling, M., Dudoit, S., Ellis, B., Gautier, L., Ge, Y., Gentry, J.*, et al.* (2004). Bioconductor: open software development for computational biology and bioinformatics. Genome Biol *5*, R80.

Gerber, H.P., Malik, A.K., Solar, G.P., Sherman, D., Liang, X.H., Meng, G., Hong, K., Marsters, J.C., and Ferrara, N. (2002). VEGF regulates haematopoietic stem cell survival by an internal autocrine loop mechanism. Nature *417*, 954-958.

Kent, D., Dykstra, B., and Eaves, C. (2007). Isolation and assessment of long-term reconstituting hematopoietic stem cells from adult mouse bone marrow. Curr Protoc Stem Cell Biol *Chapter 2*, Unit 2A 4.

Kumano, K., Chiba, S., Kunisato, A., Sata, M., Saito, T., Nakagami-Yamaguchi, E., Yamaguchi, T., Masuda, S., Shimizu, K., Takahashi, T.*, et al.* (2003). Notch1 but not Notch2 is essential for generating hematopoietic stem cells from endothelial cells. Immunity *18*, 699-711.

Notta, F., Doulatov, S., Laurenti, E., Poeppl, A., Jurisica, I., and Dick, J.E. Isolation of single human hematopoietic stem cells capable of long-term multilineage engraftment. Science *333*, 218-221.

Reya, T., Duncan, A.W., Ailles, L., Domen, J., Scherer, D.C., Willert, K., Hintz, L., Nusse, R., and Weissman, I.L. (2003). A role for Wnt signalling in self-renewal of haematopoietic stem cells. Nature *423*, 409-414.

Ryan, M.A., Nattamai, K.J., Xing, E., Schleimer, D., Daria, D., Sengupta, A., Kohler, A., Liu, W., Gunzer, M., Jansen, M.*, et al.* Pharmacological inhibition of EGFR signaling enhances G-CSF-induced hematopoietic stem cell mobilization. Nat Med *16*, 1141-1146.

Shibata, F., Goto-Koshino, Y., Morikawa, Y., Komori, T., Ito, M., Fukuchi, Y., Houchins, J.P., Tsang, M., Li, D.Y., Kitamura, T.*, et al.* (2009). Roundabout 4 is expressed on hematopoietic stem cells and potentially involved in the niche-mediated regulation of the side population phenotype. Stem Cells *27*, 183-190.

Smith-Berdan, S., Nguyen, A., Hassanein, D., Zimmer, M., Ugarte, F., Ciriza, J., Li, D., Garcia-Ojeda, M.E., Hinck, L., and Forsberg, E.C. Robo4 cooperates with CXCR4 to specify hematopoietic stem cell localization to bone marrow niches. Cell Stem Cell *8*, 72-83.

Smyth, G.K., Michaud, J., and Scott, H.S. (2005). Use of within-array replicate spots for assessing differential expression in microarray experiments. Bioinformatics *21*, 2067-2075.

Stark, C., Breitkreutz, B.J., Reguly, T., Boucher, L., Breitkreutz, A., and Tyers, M. (2006). BioGRID: a general repository for interaction datasets. Nucleic acids research *34*, D535-539.

Su, R.J., Zhang, X.B., Li, K., Yang, M., Li, C.K., Fok, T.F., James, A.E., Pong, H., and Yuen, P.M. (2002). Platelet-derived growth factor promotes ex vivo expansion of CD34+ cells from human cord blood and enhances long-term culture-initiating cells, non-obese diabetic/severe combined immunodeficient repopulating cells and formation of adherent cells. Br J Haematol *117*, 735-746.

Subramanian, A., Tamayo, P., Mootha, V.K., Mukherjee, S., Ebert, B.L., Gillette, M.A., Paulovich, A., Pomeroy, S.L., Golub, T.R., Lander, E.S., *et al.* (2005). Gene set enrichment analysis: a knowledge-based approach for interpreting genome-wide expression profiles. Proc Natl Acad Sci U S A *102*, 15545-15550.

Willert, K., Brown, J.D., Danenberg, E., Duncan, A.W., Weissman, I.L., Reya, T., Yates, J.R., 3rd, and Nusse, R. (2003). Wnt proteins are lipid-modified and can act as stem cell growth factors. Nature *423*, 448-452.

Yellaboina, S., Dudekula, D.B., and Ko, M. (2008). Prediction of evolutionarily conserved interologs in Mus musculus. BMC genomics *9*, 465.
