# Supplementary material for: Distinct Stromal Cell Factor Combinations Can Separately Control Hematopoietic Stem Cell Survival, Proliferation, and Self-Renewal
Source: Cell Rep. 2014 Jun 6;7(6):1956–67. doi: 10.1016/j.celrep.2014.05.014 (PMC4074342; doi:10.1016/j.celrep.2014.05.014)
Supplement: Document S2. Article plus Supplemental Information [file mmc2.pdf]

OPEN ACCESS
CellPress

Cell Reports
Article

Distinct Stromal Cell Factor Combinations Can Separately Control Hematopoietic Stem Cell Survival, Proliferation, and Self-Renewal

Stefan Wohrer,[1,2] David J.H.F. Knapp,[1] Michael R. Copley,[1] Claudia Benz,[1] David G. Kent,[1] Keegan Rowe,[1] Sonja Babovic,[1] Heidi Mader,[1] Robert A.J. Oostendorp,[3] and Connie J. Eaves[1,*]
[1]Terry Fox Laboratory, British Columbia Cancer Agency, Vancouver, BC V5Z 1L3, Canada
[2]Landesklinikum Wr. Neustadt, Internal Medicine 1, Wr. Neustadt 2700, Austria
[3]3rd Department of Internal Medicine, Klinikum Rechts der Isar, Technische Universität München, Munich 81675, Germany
*Correspondence: ceaves@bccrc.ca
http://dx.doi.org/10.1016/j.celrep.2014.05.014
This is an open access article under the CC BY license (http://creativecommons.org/licenses/by/3.0/).

SUMMARY

Hematopoietic stem cells (HSCs) are identified by their ability to sustain prolonged blood cell production in vivo, although recent evidence suggests that durable self-renewal (DSR) is shared by HSC subtypes with distinct self-perpetuating differentiation programs. Net expansions of DSR-HSCs occur in vivo, but molecularly defined conditions that support similar responses in vitro are lacking. We hypothesized that this might require a combination of factors that differentially promote HSC viability, proliferation, and self-renewal. We now demonstrate that HSC survival and maintenance of DSR potential are variably supported by different Steel factor (SF)-containing cocktails with similar HSC-mitogenic activities. In addition, stromal cells produce other factors, including nerve growth factor and collagen 1, that can antagonize the apoptosis of initially quiescent adult HSCs and, in combination with SF and interleukin-11, produce >15-fold net expansions of DSR-HSCs ex vivo within 7 days. These findings point to the molecular basis of HSC control and expansion.

INTRODUCTION

Hematopoietic stem cells (HSCs) represent a rare subset of undifferentiated precursors of blood cells, historically recognized by their ability to regenerate large, self-sustaining clones of mature progeny in transplanted irradiated hosts. This property has been successfully exploited to interrogate molecular mechanisms that regulate the acquisition and maintenance of the HSC state. It is also the basis of widely used hematopoietic cell transplants in patients. Not surprising, therefore, is the intense interest in defining conditions that would stimulate significant HSC expansion in vitro. Although many genes important to HSC proliferation and self-renewal have now been characterized (Xie et al., 2014), a molecular signature that specifically defines the functional state of HSCs has not been identified. Likewise, culture conditions that support significant net expansions of normal HSCs with lifelong cell output activity remain lacking.

One limitation lies in the recently appreciated heterogeneity that characterizes populations historically classified as HSCs based on their ability to produce mature blood cells for at least 4 months in transplanted hosts (Benveniste et al., 2010; Benz et al., 2012; Dykstra et al., 2007; Kent et al., 2009; Morita et al., 2010; Sanjuan-Pla et al., 2013; Yamamoto et al., 2013). Serial transplants of clonally tracked HSCs have shown that only about half of HSCs thus defined will produce sufficient daughter HSCs in transplanted primary hosts to regenerate long-term hematopoiesis in secondary mice. HSCs possessing this durability of self-renewal activity (hereafter referred to as DSR-HSCs) are selectively enriched in the lineage marker-negative (Lin^-) $CD45^+EPCR^+Sca1^+CD34^-CD49b^{low}CD48^-CD150^{2+}$ fraction of adult mouse bone marrow (BM) cells. Biologically, DSR-HSCs are distinguished by a continuing robust ability to produce mature myeloid cells independent of their lymphopoietic activity. They include most HSCs we have previously subclassified as α- or β-HSCs, and a few as γ-HSCs (Benveniste et al., 2010; Benz et al., 2012; Dykstra et al., 2007; Kent et al., 2009; Morita et al., 2010). Conversely, more limited self-renewal (LSR) activity (identified by its failure to produce sufficient HSCs to repopulate secondary mice) is a property of all HSCs subclassified as δ-HSCs and many as γ-HSCs. LSR-HSCs are selectively enriched in the $CD45^+EPCR^+Sca1^+CD34^-CD49b^{hi}CD48^-CD150^{+/-}$ fraction of adult mouse BM cells.

Survival, proliferation, and maintenance of stem cell properties are all actively regulated states of HSCs and hence likely to be important determinants of their expansion. These states are subject to regulation by external cues, some of which are provided in vivo by BM stromal cells (Mercier et al., 2012). HSC survival and, to a limited extent, self-renewal can be supported by BM stromal cells (Dexter et al., 1977; Fraser et al., 1992) or factors they secrete, including Steel factor (SF), interleukin-11 (IL-11), Flt3 ligand, Wnt3a, angiopoietin-like proteins (Angptls), thrombopoietin (TPO), fibroblast growth factor 1 (FGF1), and insulin growth factor-binding protein 2 (IGFBP2) (Audet et al., 2002; Huynh et al., 2008; Kent et al., 2008; Miller and Eaves, 1997; Reya et al., 2003; Zhang et al., 2006). However, to date, large

CrossMark

net expansions of DSR-HSCs ex vivo have not been achieved using defined factors, and the relative roles of different factors in promoting DSR-HSC viability, proliferation, and self-renewal are not understood.

To elucidate mechanisms by which stromal cells regulate key functions of HSCs, we chose the urogenital ridge-derived UG26-1B6 (UG26) cell line as a source of additional external cues because it had been found to be exceptionally potent in supporting HSCs in a contact-independent fashion (Oostendorp et al., 2002, 2005). As targets, we used $CD45^{+}EPCR^{+}CD48^{-}CD150^{+}$ (ESLAM) adult mouse cells (~40% pure HSCs; Kent et al., 2009). Our results identify nerve growth factor (NGF) and collagen 1 (Col 1) as additives that can optimize DSR-HSC survival in a defined serum-free medium (SFM) and also synergize with the mitogenic and self-renewal-promoting activity of SF and IL-11 to achieve an unprecedented expansion of total HSCs while maintaining input DSR-HSC numbers.

RESULTS

Stromal Cell-Derived Factors Enhance the SF Plus IL-11-Stimulated Expansion of DSR-HSCs

To first compare the DSR-HSC-stimulating activity of various additives reported to support adult mouse BM HSC expansion in vitro, we set up test cultures with 30 ESLAM cells each and then 7 days later, performed limiting dilution transplant assays to determine the numbers of DSR-HSCs, as well as the total HSCs present (Figure 1A). HSCs were defined as cells whose progeny constituted >1% of all the nucleated peripheral blood (PB) cells present 16–24 weeks posttransplant. DSR-HSCs were defined as the subset of HSCs that generated >1% of all the circulating granulocyte and monocyte (GM) cells present 16–24 weeks posttransplant (and LSR-HSCs as all the other HSCs). Single-cell transplants indicated that the 30 input ESLAM cells contained, on average, 12 total HSCs of which 8 were DSR-HSCs. The results for the cultured cells showed that DSR-HSC numbers increased significantly above input (29-fold; p < 0,001) when UG26 cells were present together with SF plus IL-11 and to a slightly, but not significantly (p = 0.19), lesser extent (15-fold) when the UG26 cell conditioned medium (CM) was added instead of the UG26 cells. Total HSC numbers were similarly and also significantly increased in both of these cultures: 20- and 11-fold, respectively (p < 0.001; Figure 1B, left and middle panels). Representative fluorescence-activated cell sorting (FACS) profiles of cells regenerated in recipients of cells cultured in SF plus IL-11 plus UG26 cells are shown in Figure 1C, and lineage-specific reconstitution kinetics of donor cells cultured in SF plus IL-11 plus UG26 cells, or SF plus IL-11 plus CM, or SF plus IL-11 only, are shown in Figure 2. Interestingly, ESLAM cells cultured in UG26 cells alone showed maintenance but not expansion of DSR-HSC numbers (and of total HSCs, Figure 1B). In contrast, DSR-HSC numbers showed a net decrease when cultured in any of the four other factor combinations tested, although in some, total HSC numbers were maintained at, or close to, input levels (Figure 1B).

To evaluate more stringently the DSR property of the in-vitro-expanded HSCs, we determined the number of additional daughter HSCs produced in the primary recipients of the cultured cells. This involved performing another set of limiting dilution HSC transplant assays on BM cells harvested from each group of primary recipients 24 weeks after they had been initially transplanted with cells harvested from the 7-day cultures. The results showed that cultures to which either UG26 cells or UG26 CM had been added to the SF plus IL-11 cocktail produced HSCs in vitro that were capable of extensive further expansion in the primary hosts (Figure 1B, right panel) and, hence, gave a high level of repopulation of secondary recipients (Figure 1C, lower panels). Thus, the overall DSR-HSC expansion achieved (first in vitro, and then in the primary recipients) when UG26 cells were present together with SF plus IL-11 was 130-fold, and 360-fold when UG26 CM was added (assuming one femur represents 5% of the total BM of a mouse; Colvin et al., 2004). These sustained HSC expansions obtained with UG26 cells or CM were again not significantly different from each other (p = 0.13). In contrast, no secondary repopulating activity was detected in comparable assays of BM cells from primary recipients of cells cultured with any of the defined growth factor cocktails.

These findings document the ability of factors produced by UG26 cells in combination with SF plus IL-11 to produce a rapid and significant net expansion in vitro of serially transplantable HSCs. They also demonstrate that this effect can be mediated by a mechanism that is cell contact independent.

UG26 Cells Produce Factors that, in Combination with SF Plus IL-11, Enable All HSC Differentiation Programs to Be Sustained

To determine the frequency of ESLAM cells that can generate transplantable HSC progeny in the presence of SF plus IL-11 with or without UG26 cells, we set up a second series of cultures with a single ESLAM cell each and then transplanted the entire contents of each (regardless of how many cells it contained) into separate irradiated recipients (Figure 3A). Analysis of the PB of these mice 16 weeks later showed that both α and β patterns of differentiation were obtained from the cultures to which UG26 cells had been added and at a frequency not significantly different from the frequency of α- and β-HSCs in the input ESLAM cells (18% versus 28%; p = 0.12; Figure 3B). Interestingly, the proportion of cultures that contained any type of HSC was significantly higher than the frequency of total HSCs in the original ESLAM cells (72% versus 40%; p < 0.001; Figure 3B). Thus, some ESLAM cells that are not directly detectable as HSCs can, nevertheless, generate progeny that have the functional properties of HSCs in vivo. In contrast to the cultures that contained SF plus IL-11 plus UG26 cells, only 13% of the cultures that contained only SF plus IL-11 contained any HSCs (a value significantly <40%, which was the input HSC frequency; p < 0.001), and all of these latter HSCs produced a δ pattern of reconstitution.

As a more direct test of the frequency of ESLAM cells that are responsive to the factors produced by UG26 cells in concert with SF plus IL-11, we set up another series of single-cell cultures, in this case, with SF plus IL-11 with or without UG26 CM (36 with and 12 without UG26 CM; Figure 4A). These were visually monitored every few hours for the next 4½ days until a first division occurred. Pairs of daughter cells were thus identified and then

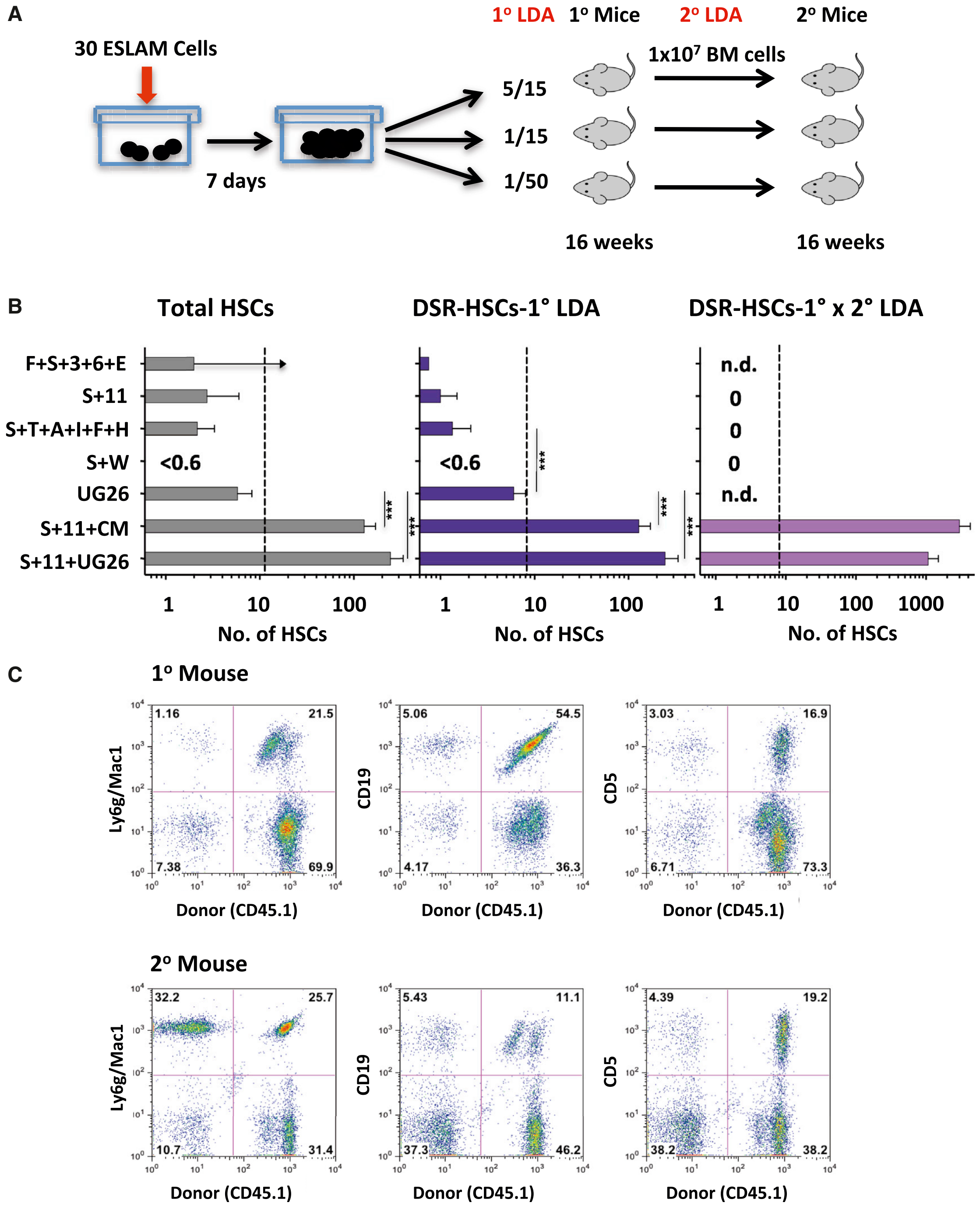

Figure 1. HSC Numbers Produced in 7-Day Cultures of ESLAM Cells Containing Different Supplements

(A) Experimental design.

(B) Results of 16-week limiting dilution transplant assays used to determine the outputs of HSCs and DSR-HSCs (left 2 panels) and the cumulative DSR-HSC expansion obtained first in vitro and then in primary (1°) recipients (right panel) (12 mice/condition/experiment, 3–5 experiments/condition, mean and SEM for each condition). Dotted lines show the total and DSR-HSCs estimated to be present in the 30 input ESLAM cells. Holm-corrected pairwise significance values are shown (*p = 0.05; **p = 0.01; ***p < 0.001). Where a limiting dilution was not reached, the bar indicates the minimal HSC value detectable with an upward arrow. Supplements were as follows: F+S+3+6+E (15% FBS plus 50 ng/ml SF plus 10 ng/ml IL-3 plus 10 ng/ml IL-6 plus 3 U/ml Epo); S+11 (100 ng/ml SF plus 20 ng/ml plus IL-11); S+T+A+I+F+H (10 ng/ml SF plus 20 ng/ml TPO plus 100 ng/ml Angptl3 plus 500 ng/ml IGFBP2 plus 10 ng/ml FGF1 plus 10 μg/ml heparin [H]); S+W (30 ng/ml SF plus 100 ng/ml Wnt3a); UG26 cells; CM (50% UG26 CM); and S+11+CM (SF plus IL-11 plus CM).

(C) Representative FACS profiles of PB cells obtained 16 weeks after transplanting primary and secondary mice with cells harvested from cultures containing UG26 cells plus SF plus IL-11, described in (B). The following markers were used to investigate donor chimerism: Ly6g/Mac1 (GM cells), CD19 (B cells), and CD5 (T cells).

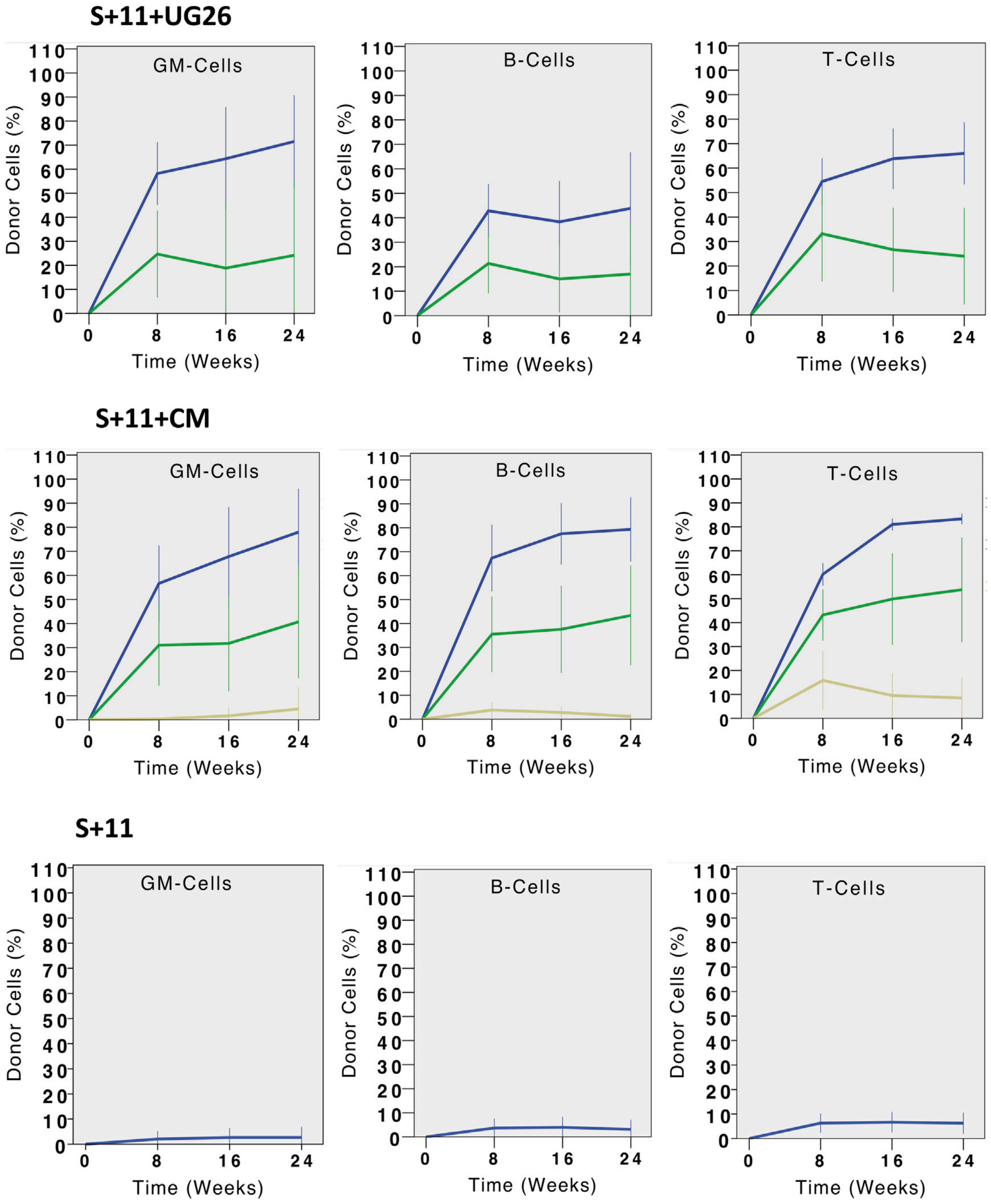

Figure 2. Different Reconstitution Kinetics in Mice Transplanted with Matched Progeny Outputs of ESLAM Cells Cultured for 7 Days with Different Stimuli

Geometric mean percentage donor contributions to the total circulating GM, B, and T cells measured at different times after transplanting groups of mice with the progeny of 30 ESLAM cells cultured as described in Figure 1. Error bars indicate the range defined by ±2 SEM. Blue, green, and brown lines indicate mice that received $1/15^{th}$, $1/50^{th}$, and $1/90^{th}$ of a culture each, respectively.

separately transplanted pairwise into each of two irradiated recipients. In the 36 pairs of cells produced in the cultures containing SF plus IL-11 plus UG26 CM, 17 (47%) were found to contain at least 1 HSC, and 9 of these pairs (25%) produced at least 1 α- or β-HSC (Figure 4B). Overall, the frequencies and distributions of HSC subtypes in the first division progeny were similar to the HSCs both in the starting ESLAM cells and in the 7-day clones generated under similar conditions (Figure 4C). Secondary limiting dilution transplantation assays of the cells regenerated from the first division progeny pairs confirmed their DSR

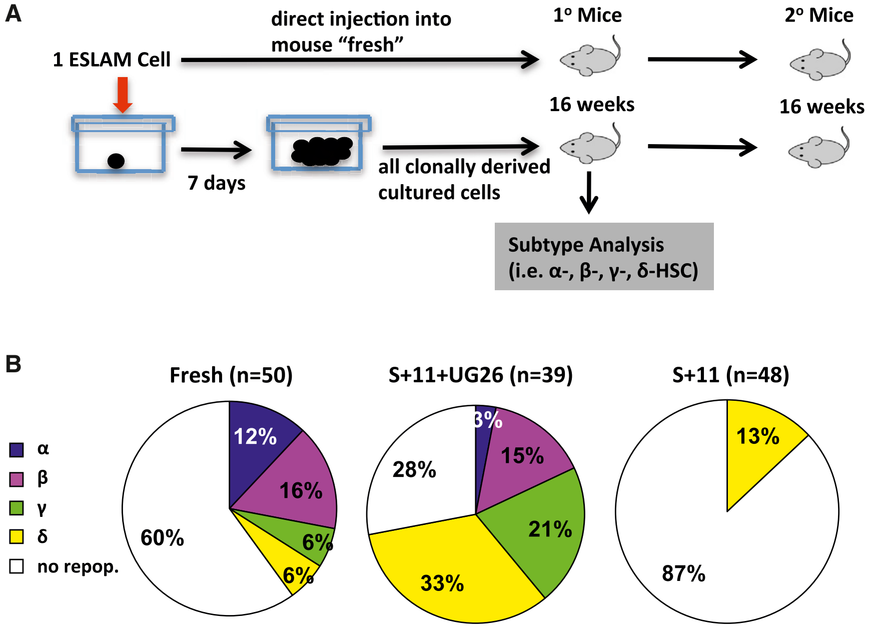

Figure 3. Effect of Different Supplements on the Frequency of Single ESLAM Cells that Generate HSCs in 7-Day Cultures

(A) Experimental design.

(B) Frequency of HSCs in each sample and the differentiation patterns obtained from them. Left pie chart shows input ESLAM population (50 cells tested). Middle pie chart shows 7-day cultures initiated with single ESLAM cells stimulated with UG26 cells plus SF plus IL-11 (39 clones tested). Right pie chart shows 7-day cultures initiated with single ESLAM cells stimulated with SF plus IL-11 only (48 clones). The definitions used to distinguish α, β, γ, and δ patterns of differentiation are given in the Supplemental Experimental Procedures. Results are pooled from three to six experiments. See also Figure S1 for secondary transplant results. repop., repopulation.

and LSR attributes assigned on the basis of their clonal GM contributions in the primary mice (Table 1; Figure S1). In contrast, none of the 24 mice injected with the 12 pairs of first division progeny of cells cultured in SF plus IL-11 was repopulated. From the paired daughter cell tracking, we also found more HSCs present in the progeny of cells that completed a first division after >48 hr in culture (67% versus 16% for those that completed a first division in <48 hr; $p = 0.007$) and reached 100% for cells that did not divide until after 96 hr.

We also determined the effect of adding UG26 CM to SF plus IL-11 on the proportion of first division progeny of ESLAM cells that would display long-term culture-initiating cell (LTC-IC) activity in a 6-week assay (Figure 5A). The frequency of LTC-ICs in the starting ESLAM cells (89 out of 213 [42%], Figure 5B) was similar to the frequency of total HSCs (40%; $p = 0.87$; Figure 3B), and the frequency of ESLAM cells that produced at least 1 daughter LTC-IC was again higher than the input LTC-IC frequency (70 out of 96 [73%]; $p < 0.001$) and also higher than the frequency of ESLAM cells that generated at least 1 daughter HSC (47%; $p < 0.001$; Figure 3D). The frequency of pairs in which both daughter cells were LTC-ICs (28%) was also higher than the frequency of pairs containing two HSCs (14%; $p = 0.023$). These disparities in the apparent effects of UG26 CM plus SF plus IL-11 on LTC-ICs and HSCs could be due to the selective inability of HSCs to engraft when they are in S/G_2/M phases of the cell cycle (Bowie et al., 2006) but may also reflect a broader range of cells detected as LTC-ICs.

Different Factors Separately Regulate HSC Survival and Mitogenesis

To interrogate the biological mechanism(s) by which the factors produced by UG26 cells might enable an in vitro expansion of DSR-HSC numbers, we initiated another series of cultures with single ESLAM cells and then monitored them visually at intervals over the next 4½ days (108 hr) to track the persistence of viable (refractile) cells and their rate of entry into a first division during that period (Figure 6A). At the end of 4½ days, we added medium containing fetal bovine serum (FBS) plus SF plus IL-3 plus IL-6 plus erythropoietin (Epo) as a further stimulus to promote the formation of readily detectable differentiating clones from persisting viable cells.

In the presence of SF plus IL-11 plus UG26 CM, 97% of the input cells (279 out of 288 cells in 3 experiments) survived and executed a first division between 24 and 108 hr after being placed in vitro (Figure 6B). Results were indistinguishable for 168 single ESLAM cells cultured in FBS plus SF plus IL-3 plus IL-6 plus Epo (96% survival with clonogenic activity), despite the inability of these conditions to support the retention of DSR-HSC activity (Figure 1A). In contrast, 64% (184 out of 288) of the single ESLAM cells cultured in SF plus IL-11 alone could no longer be visualized at the end of the first 12 hr in vitro. However, the remaining 36% of these cells remained refractile and appeared viable for the first 18 hr and then began to divide with the same kinetics as the cells maintained in SF plus IL-11 plus UG26 CM. These (and only these) went on to produce large colonies when the FBS plus SF plus IL-3 plus IL-6 plus Epo cocktail was added at the end of the first 4½ days. Interestingly, 90% (259 out of 288) of the cells initially cultured in UG26 CM alone (without SF plus IL-11) did not divide, and most also became smaller over time. Nevertheless, at the end of the first 4½ days, all of these cells plus another 12 thought to be dead (i.e., a total of 271 of the original 288) could still be stimulated to produce readily detectable colonies upon the addition of the FBS plus SF plus IL-3 plus IL-6 plus Epo cocktail.

To determine whether the survival advantage afforded by factors present in UG26 CM might be restricted to cells that are quiescent, we set up new single ESLAM cell cultures in SF plus IL-11 plus UG26 CM for 4½ days (by which time every cell had completed at least one division) and then switched the medium to SFM with SF plus IL-11 alone for a second 4½ days. Continued monitoring of each of these wells over the second

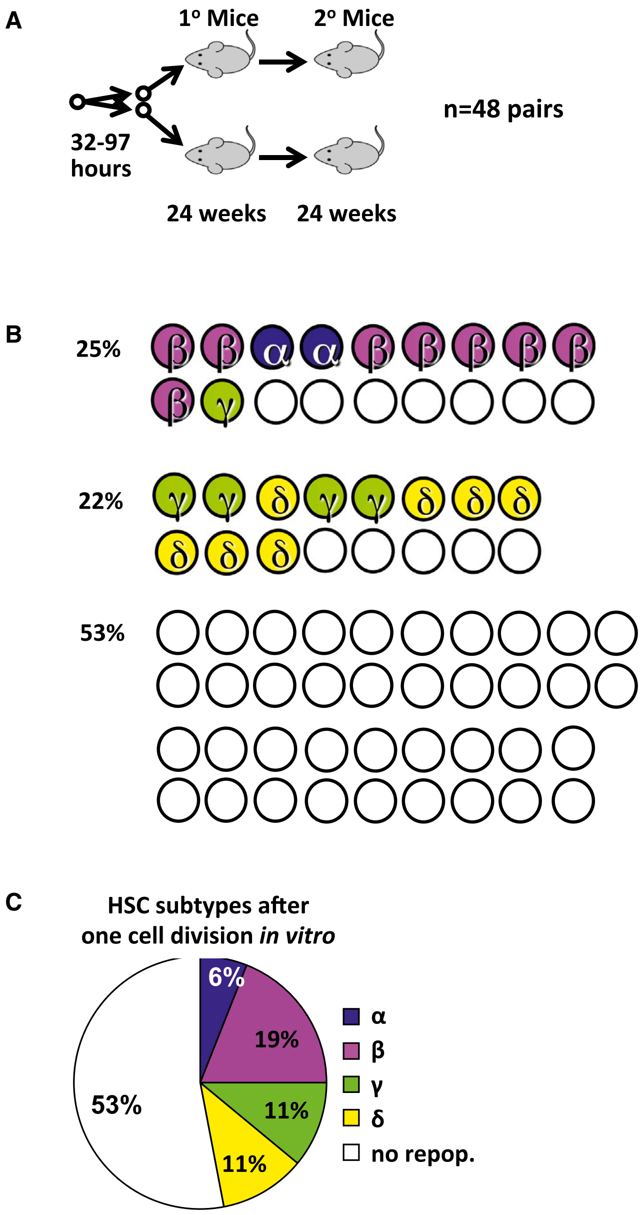

Figure 4. UG26 CM Enhances the Retention of DSR-HSC Functionality in the First Division Progeny of Single ESLAM Cells

(A) Experimental design. A total of 36 ESLAM cells were cultured as single cells in UG26 CM plus SF plus IL-11 and 12 in SF plus IL-11 only. Both cells in doublets produced 32–97 hr later were then assayed individually for HSC activity in separate mice. Twenty-four weeks later, BM cells from DSR-HSC-repopulated primary mice were harvested and secondary transplantations performed.

(B) Distribution of α, β, γ, or δ HSC subtypes in each first-generation pair of ESLAM daughter cells in which at least one daughter cell was an HSC of any type. Because none of the 24 mice that received a cell cultured in SF plus IL-11 showed engraftment, only results for cells cultured in UG26CM plus SF plus IL-11 are shown.

(C) Distribution of the types of inferred input ESLAM cells classified according to the α, β, γ, or δ HSC subtypes that they produced in their first-generation progeny, as shown in (B). When both progeny were HSCs, the initial ESLAM cell was classified as the more primitive subtype (α, β, γ, δ—in that order).

Table 1. Serial Tracking of the HSC Subtypes Produced by ESLAM Cells Stimulated to Execute a First HSC Self-Renewal Division In Vitro

First Division HSC Subtypes Detected in Primary Mice	Progeny HSC Subtypes Detected in Secondary Mice
DSR-HSCs (n = 15)	73% (11 out of 15) DSR-HSCs
	27% (4 out of 15) LSR-HSCs
LSR-HSCs (n = 7)	0% (0 out of 7) no repopulation

The left column refers to the DSR- and LSR-HSC subtypes identified among the first-division progeny of ESLAM cells generated in vitro as determined from their reconstituting properties in primary recipients (as shown in Figure 4B). The right column refers to the DSR activity seen in secondary recipients of cells transplanted with BM harvested from the primary mice.

4½ days showed that 97% (279 out of 288) of the wells contained cells that continued to divide. Moreover, the range of times taken to complete the next division was the same as for the first division initiated in SF plus IL-11 plus UG26 CM, discounting the initial lag, and further addition of FBS plus SF plus IL-3 plus IL-6 plus Epo medium at the end of the second period of monitoring showed that 97% of the cultures again produced a very large clone over the following 7 days (Figure 6C).

These results indicate that SFM containing SF plus IL-11 only is unable to support the survival of a large proportion of quiescent adult HSCs, although once activated, both their viability and their proliferation can be efficiently sustained by continued exposure to SF plus IL-11. However, this initial loss of quiescent adult BM ESLAM cells can be circumvented by exposure to UG26 CM or FBS plus IL-3 plus IL-6 plus Epo, although these two sources of prosurvival factors clearly differ in their mitogenic activities and in their abilities to sustain HSC self-renewal activity. Interestingly, the overall rate at which initially quiescent ESLAM BM cells enter the cell cycle appeared independent of any of these conditions of stimulation.

NGF and Col 1 Can Partially Replace UG26 CM in Maintaining DSR Activity during HSC Expansion In Vitro

As a first step toward identifying the prosurvival factors produced by UG26 cells, we looked for pathways that differentially activate as well as related candidate effectors secreted by UG26 cells and their possible receptors in purified adult BM ESLAM cells. To this end, we generated gene expression profiles for adult BM ESLAM cells before and 6 hr after being placed in culture with or without UG26 CM with or without SF plus IL-11. The 6 hr time point was chosen to obtain cells before evidence of their death is obvious when they are cultured in SF plus IL-11 alone (Lecault et al., 2011). We then compared these profiles with each other, as well as with a published gene expression profile for UG26 cells (Ledran et al., 2008) (Figure 7A).

Analysis of the profiles obtained for ESLAM cells stimulated with UG26 CM (with or without SF plus IL-11) compared to fresh ESLAM cells and ESLAM cells stimulated with SF plus IL-11 alone identified a total of 250 (of 430 tested) REACTOME pathways for which some members showed significantly altered transcript expression ($p < 0.05$) in cells maintained in UG26 CM with or without SF plus IL-11 (Table S1). These pathways

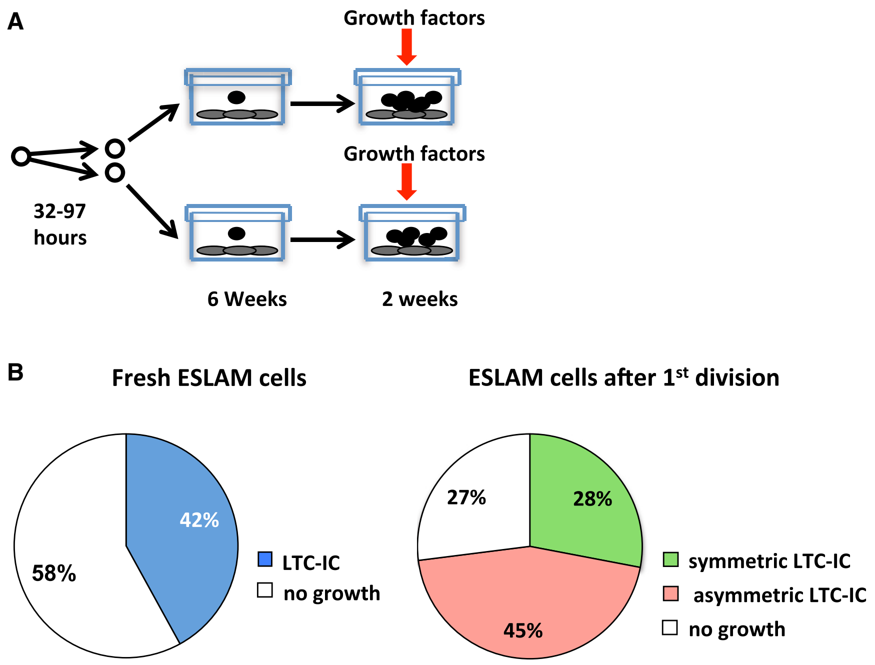

Figure 5. UG26 CM and SF plus IL-11 Support the Frequent Production of LTC-ICs in Both Progeny of Single ESLAM Cells Stimulated to Divide In Vitro

(A) Experimental design. A total of 96 ESLAM cells were cultured as single cells in UG26 CM and SF plus IL-11 until they divided a first time as in Figure 4. Doublets were then transferred into 192 separate wells containing UG26 cells and assayed for LTC-IC activity.

(B) Pie charts showing the frequencies of LTC-ICs (light-blue fraction) as determined from assays of single freshly isolated ESLAM cells (left), and the frequencies of ESLAM cells that produced two, one, or no LTC-ICs in their first division progeny (right).

included cell-cycle progression and metabolic pathways, signaling pathway activation, apoptosis-related pathways, and RNA processing/splicing pathways (Table S2). To identify specific candidate factors, we used Gene Ontology annotations to select transcripts present in UG26 cells that were categorized as encoding proteins in the "extracellular region" and that had anticipated interactions with expressed genes annotated as having "receptor activity" in the ESLAM cells. From this analysis, we identified 172 candidate factors (Table S3). From a survey of the current stem cell literature, a comparison of candidate factors to the differential expression of downstream pathways, and factor availability, we then selected a subset of 12 of the 172 candidate proteins for further testing of their ability to block the death of HSCs as previously shown for UG26 CM (Figure 6A). Among the 12 candidates tested, we found NGF and Col 1 to be the most effective substitute for UG26 CM (Figure 7B), and when present together, 97% of the input ESLAM cells remained viable. Both NGF and Col 1 also mimicked the complete lack of mitogenic activity of the UG26 CM (data not shown).

Assessment of ESLAM cells in 36 hr cultures containing SF plus IL-11 with or without UG26 CM, NGF, and Col 1, or FBS plus SF plus IL-3 plus IL-6 plus Epo showed that many cells (20%) were Annexin V$^+$ when only SF plus IL-11 was present, i.e., only slightly fewer than the proportion of dead cells determined both by immediate visual inspection and by their subsequent failure to form colonies in the presence of added growth factors (Figure 6B). In contrast, the proportion of Annexin V$^+$ cells was significantly decreased ($p \leq 0.03$) in all cultures to which the prosurvival factors were added, including NGF plus Col 1 (Figure 7C).

To determine whether NGF and Col 1 can replace the ability of UG26 CM to promote HSC self-renewal divisions in vitro in the presence of SF plus IL-11, we set up a final series of 7-day cultures with 1 or 30 ESLAM cells each and assayed the HSC output using the same protocol as in Figure 1A. Primary recipients of these cultured cells showed that the addition of NGF plus Col 1 to SF plus IL-11 maintained input numbers of DSR-HSCs in the 7-day cultures and produced a 4-fold net expansion of total HSCs ($p = 0.48$ and < 0.001, respectively; Figure 7D). Although these values are not as high as those achieved with UG26 CM ($p < 0.001$ and $= 0.01$, respectively; Figure 1B), secondary transplant assays confirmed the continuing DSR activity of the HSCs maintained using NGF plus Col 1 (data not shown). Analyses of another 23 mice transplanted with clones generated from single ESLAM cells cultured for 7 days in SF plus IL-11 plus NGF plus Col 1 showed that 17 of the clones (74%) contained HSCs, and 3 clones (13%) contained DSR-HSCs that included the DSR-HSC-associated β pattern of differentiation (Figure 7E).

DISCUSSION

Stromal Cells Produce Factors that Synergize with SF and IL-11 to Promote DSR-HSC Expansion In Vitro

Evidence that HSCs are regulated by nonhematopoietic stromal cells in vivo dates back many decades to transplantation experiments performed with *Sl/Sl^d* mice (McCulloch et al., 1965; Sutherland et al., 1970). Many products of stromal cells and related cell types have now been implicated in the regulation of the HSC compartment in vivo (Mercier et al., 2012). Likewise, the most successful strategies for maintaining HSCs long term in vitro have involved their coculture with primary stromal cells or stromal cell lines from various sources (Dexter et al., 1977; Fraser et al., 1990, 1992; Moore et al., 1997; Ploemacher et al., 1989; Wineman et al., 1996). The recent identification of distinct subsets of stromal cells with variable roles in regulating different HSC functions in adult BM now raises the interesting possibility that they regulate HSCs via non (or incompletely)-overlapping mechanisms (Ding and Morrison, 2013; Guezguez et al., 2013; Kunisaki et al., 2013).

Here, we identify different combinations of factors secreted from stromal cells that differentially support biologically distinct

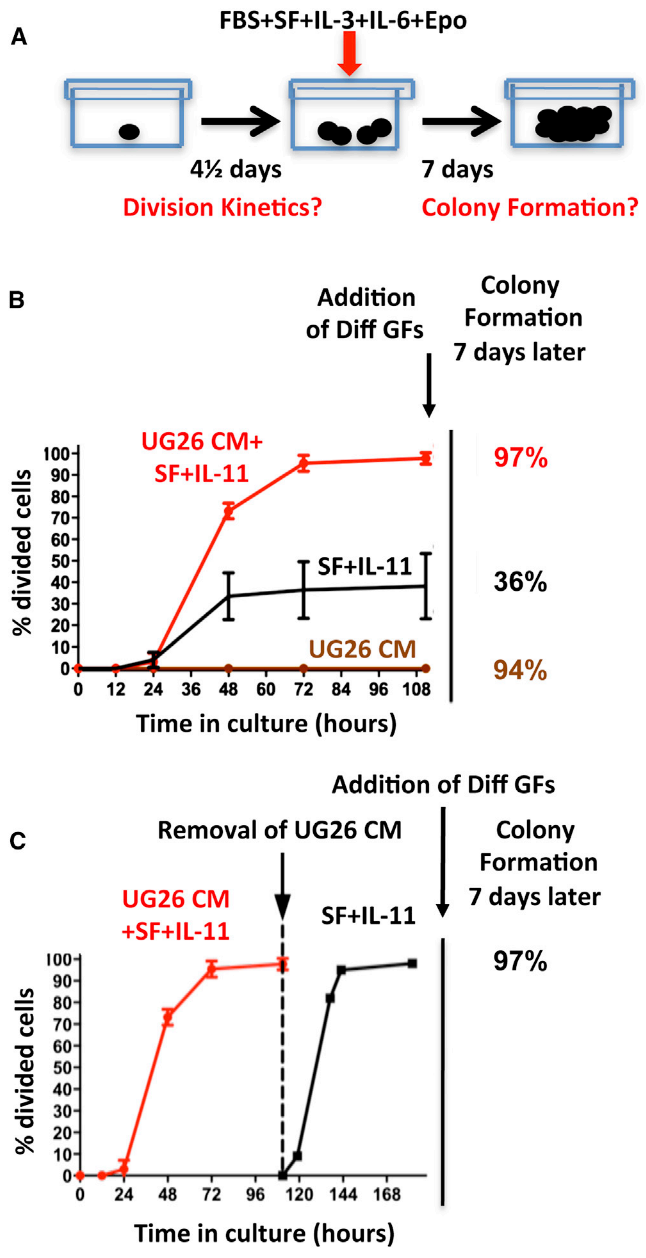

Figure 6. Comparison of the Different Mitogenic and Prosurvival Activities of UG26 CM, SF plus IL-11, and the Combination on ESLAM Cells in Single-Cell Cultures

(A). Experimental design.

(B) Cumulative plots of the kinetics with which single ESLAM cells completed a first division when cultured either in UG26 CM+SF+IL-11, or SF+IL-11 only, or UG26 CM (CM) only (5 experiments, 96 cells in each). Error bars show 95% confidence intervals (CI). Addition of Diff GFs, addition of different growth factors.

(C) Plot showing the kinetics with which single ESLAM cells completed a next division after they had been cultured for an initial 108 hr in UG26 CM+SF+IL-11 (i.e., until each cell had already divided at least once), when SFM and SF+IL-11 were added to replace the initially added UG26 CM+SF+IL-11 (after washing the cells twice with SFM). Results are from 3 experiments (96 cells in each).

HSC functions. These functions are controlled intrinsically by separate, albeit likely interconnecting, pathways, although all are important to the speed and extent to which HSCs can expand their numbers. Specifically, they are responsible for the maintenance of HSC viability, the response of HSCs to factors that control their cycling state, and the maintenance in HSCs of a continuing poised, but undifferentiated, state. Clonal analysis and secondary transplant assays of the cells produced in vitro under conditions that support all three of these functions (i.e., SF plus IL-11 plus either UG26 cells or UG26 CM or NGF plus Col 1) demonstrated a significant net expansion of adult mouse HSCs with either maintenance or expansion of HSCs with DSR properties. In contrast, in the absence of UG26 cells or UG26 CM or NGF plus Col 1, even full maintenance of ESLAM (and hence HSC) survival and mitogenesis (as could be achieved by exposure to FBS plus SF plus IL-3 plus IL-6 plus Epo) was not sufficient to prevent a rapid and significant loss of DSR activity. In addition, we found that the kinetics of mitogenesis was not altered even when conditions failed to support the survival of >50% of the cells in the first 24 hr in vitro (i.e., in SF plus IL-11). We also used analysis of split doublets to formally document the execution of first divisions that produce two DSR-HSCs under conditions where survival, proliferation, and self-renewal are all well supported. These findings thus represent a major advance over previously reported results with "optimal" cytokine cocktails (SF plus IL-11, SF plus TPO plus Angptl3 plus IGFBP2 plus FGF plus H, SF plus Wnt3A) that we have now shown do not sustain DSR-HSC activity.

Until recently, the speed with which many adult mouse BM HSCs die (in the first 12 hr) when they are incubated under conditions generally used to stimulate their rapid entry into the cell cycle had not been widely appreciated. In retrospect, this finding may account for historic difficulties in obtaining pure mouse HSC populations and the low yields accompanying more promising approaches. Surprisingly little is known about the specific regulation of HSC viability beyond the level of expression of particular genes with identified roles in general cell survival control and evidence of their activation in leukemia (Jordan and Guzman, 2004). A notable exception was an early study suggesting an ability of Bcl-2 to delay HSC apoptosis and synergize with SF to maintain HSC survival (Domen et al., 2000). We did not find evidence of upregulated Bcl-2 in the HSCs treated with UG26 CM, but this is not surprising because Bcl-2 has not been implicated in the physiological control of HSCs. It is thus inviting to speculate that NGF plus Col 1 and UG26 CM may modulate similar downstream pathways to block apoptosis, as suggested by our finding of a differential expression of genes in the "Apoptosis" REACTOME pathway following UG26 CM exposure, and the similar relative decrease in Annexin V staining of ESLAM cells incubated with either of these additives.

The Effects of Stromal Factors on HSC Self-Renewal In Vitro Are Manifest within the First Cell Cycle and Act to Preserve the HSC Lineage Program as well as Their DSR State

The early death of HSCs appeared complete, even before any of these cells began to divide—consistent with a significant dissociation in the signaling pathways that promote survival and

OPEN ACCESS
CellPress

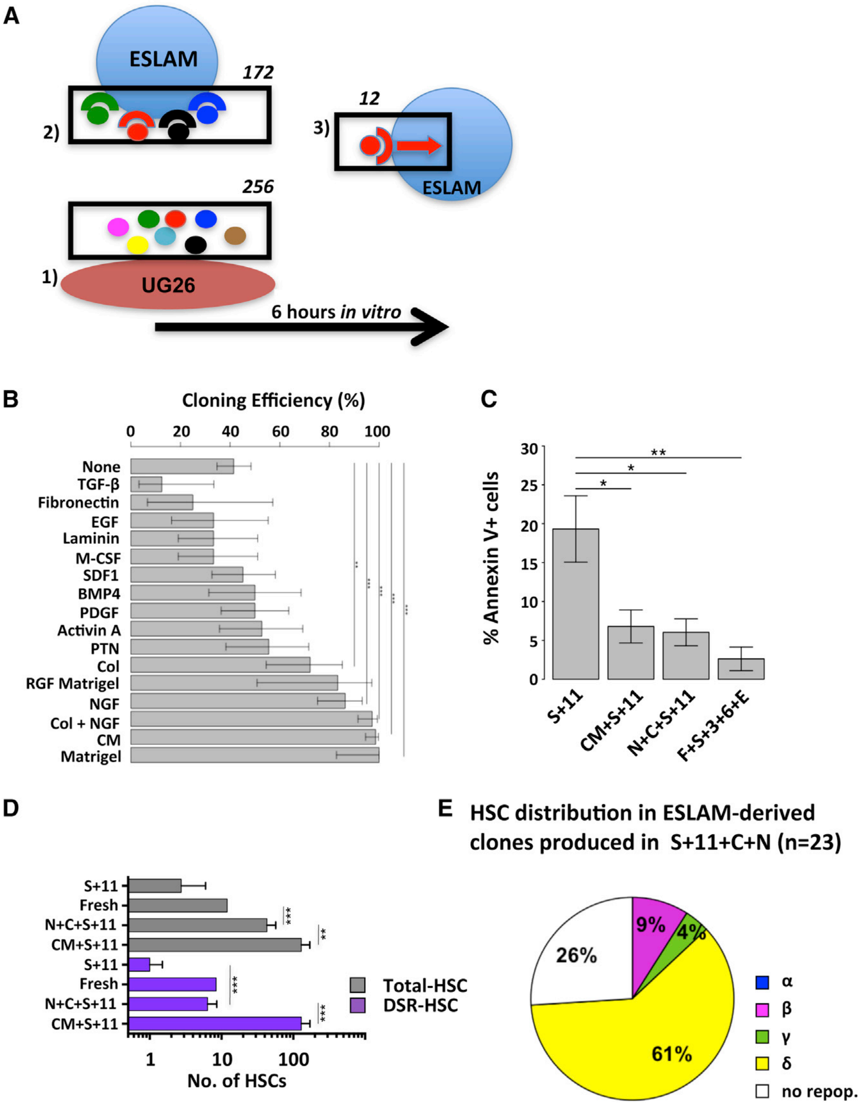

Figure 7. Col 1 and NGF Can Substitute for UG26 CM to Support DSR-HSC Self-Renewal In Vitro

(A) Graphical display of the algorithm used to identify significantly changed pathways from Affymetrix gene chip data obtained on stromal cells and input and responding ESLAM cells. Numbers above the boxes indicate the number of sequentially identified genes. Additional details are provided in the text and Tables S1–S3.

(B) Comparison of the ability of different factors to enhance the survival and mitogenesis of isolated ESLAM cells cultured in SFM with SF plus IL-11 for 7 days; 12–144 cells analyzed per test condition. A 95% CI was generated using the R function "prop.test."

(C) Proportion of Annexin V^+ cells in 36 hr cultures of ESLAM cells incubated in SFM plus the additives shown. Mean ± SEM values from four experiments are shown.

(D) Outputs of HSCs from 7-day cultures initiated with 30 freshly isolated ESLAM cells compared to input values using the same experimental design as in Figure 1A (12 mice/condition/experiment and 3–5 experiments/condition). White bars indicate total HSCs, and purple bars show DSR-HSCs. Values shown are the mean ± SEM. Additions were SF+IL-11 (S+11), NGF+Col 1+SF+IL-11 (N+C+S+11), or UG26 CM+SF+IL-11 (CM+S+11) at the same concentrations as in Figure 1. A 95% CI was generated using ELDA.

(E) Distribution of HSC activity in clones derived from 23 single ESLAM cells cultured in SF plus IL-11 plus Col 1 plus NGF for 7 days determined from 16-week transplantation assays of single clones transplanted into individual mice.

In (B) and (D), Holm-corrected pairwise significance values are shown (*p = 0.05; **p = 0.01; ***p < 0.001).

mitogenesis. This inference is further supported by the finding that both UG26 cells and NGF plus Col 1 have potent prosurvival HSC activity in the absence of any mitogenic effect on the HSCs thus "protected." The maintenance of DSR potential also appears to be regulated independent of the control of this early prosurvival effect on initially isolated quiescent adult HSCs because the FBS plus SF plus IL-3 plus IL-6 plus Epo cocktail was similarly able to prevent early death of HSCs but induced a rapid loss of their self-renewal property. Analysis of paired daughter cells of individual input ESLAM cells further showed that they required early exposure to UG26 CM (in their first cell cycle) to retain a DSR-HSC state. This is an important extension of our previous observation that abrogation of all HSC activity can be obtained even before the cells complete a first cell cycle if they are exposed to suboptimal concentrations of SF (Kent et al., 2008). Together, these results suggest that survival and maintenance of DSR competence in quiescent HSCs depend on their continuous exposure to different external factors that act via pathways (or pathway elements) that may not even require, nor involve, entry into the cell cycle.

Of additional interest is the observation that the time taken for mitogenically stimulated ESLAM cells to complete a first mitosis is positively associated with the likelihood that at least one of their two daughter cells will retain HSC functionality. This is consistent with previous evidence that longer cell-cycle transit times correlate with the most primitive HSCs (Dykstra et al., 2006; Lutolf et al., 2009; Yamazaki et al., 2009). Such associations suggest the possibility that cell-cycle control, like retention of GM differentiation potential, may be mechanistically linked to DSR competence in adult mouse BM HSCs.

Our findings are also potentially relevant to understanding the role of transplantation assays in detecting cells with the molecular machinery required for HSC activity. Single-cell transplantation experiments, confirmed here, have consistently shown that approximately half of these cells are detectable as HSCs, whereas the other half is not. However, as now revealed, nearly all FACS-purified ESLAM cells can display extensive proliferative potential in vitro, even though only half is detectable as LTC-ICs

in a 6- to 7-week assay (Kent et al., 2009). Moreover, the frequency of ESLAM cells that can respond to SF plus IL-11 in the presence of UG26 CM in vitro by generating progeny HSCs that are functional in vivo is significantly higher than the 40% of freshly isolated ESLAM cells that are directly detectable in vivo as HSCs. Taken together, this raises the possibility that most adult BM ESLAM cells have not irreversibly lost the molecular status of HSCs.

Overall, our results suggest that several core HSC behavioral programs can be functionally uncoupled, allowing their differential and combinatorial activation by an array of external factors. This differential program activation could result from activation of multiple independent signaling pathways (a combinatorial switch mechanism), by different levels of activation in a few common pathways (a cellular rheostat-like mechanism), or by some combination of these two. In either case, additional downstream molecular interactions are likely. The various genes and pathways previously implicated in HSC maintenance/expansion are consistent with a combinatorial mechanism operating to control HSCs (Reya et al., 2003). Similarly, the fact that low concentrations of SF provide HSC survival benefits, whereas maintenance of repopulation potential requires high levels of SF, supports the existence of mechanisms that depend on different signaling thresholds in these cells (Kent et al., 2008).

Implications for Future Improvement of HSC Expansion Protocols

Our findings reemphasize the deficiency of 4- to 6-month PB repopulation endpoints that do not specifically measure the output of donor-derived GM cells in order to distinguish between HSCs that have retained or lost DSR activity, as recently highlighted by others (Yamamoto et al., 2013). They also demonstrate that mouse DSR-HSC self-renewal divisions can be achieved under defined conditions in vitro in the absence of any other cells, but to achieve this response, multiple extrinsic factors are required. Interestingly, at least some of these factors, exemplified by Col 1 (Hu et al., 2011) and NGF (García et al., 2004), may be ubiquitously prevalent extracellular matrix components of the interstitial space within hematopoietic tissue. Recent studies have highlighted a differential expression on the surface of HSCs and their closely related downstream derivatives of several integrins, which are receptors for such proteins (Benveniste et al., 2010; Notta et al., 2011; Wagers and Weissman, 2006). Thus, factors that activate these receptors may constitute an additional strategy for enhancing HSC expansion, as suggested by others (Celebi et al., 2011; Kurth et al., 2011; Umemoto et al., 2012). The ability of a combination of defined soluble proteins to promote HSC expansion in vitro refutes the hypothesis that cell contact is required to mediate such responses and should facilitate future interrogation of the mechanisms involved in the maintenance of the DSR state when HSCs are stimulated to proliferate.

EXPERIMENTAL PROCEDURES

Mice

C57Bl/6J (B6)-*Ly5.1* or C57Bl/6J (B6)-*Ly5.2* mice and congenic B6-*W41/W41-Ly5.1* and B6-*W41/W41-Ly5.2* (*W41-5.1* and *W41-5.2*, respectively) mice were bred and maintained in our animal resource center in microisolator cages and provided with continuous sterile food, water, and bedding. Procedures for isolating ESLAM cells from adult mouse BM and performing and assessing transplants were as previously described (Benz et al., 2012; Kent et al., 2009) and carried out with approval from the University of British Columbia Animal Care Committee. For further details, see Supplemental Experimental Procedures.

UG26 Cells and CM

UG26 stromal cells were cultured as previously described (Oostendorp et al., 2002) and CM obtained from confluent UG26 cells X-irradiated with 30 Gy and then incubated for 3 days with SFM after removal of the UG26 culture medium and rinsing the cultures several times with PBS. The CM was then filtered through a 40 μm cell strainer (Becton Dickinson) and stored frozen at −20°C.

ESLAM Cell Cultures

ESLAM cells were deposited into the round-bottomed wells of 96-well plates using the single-cell deposition unit of the sorter, each well having been preloaded with 100 μl of SFM (Iscove's medium with 10 mg/ml BSA, 10 μg/ml insulin, and 200 μg/ml transferrin, 40 μg/ml low-density lipoproteins, 100 U/ml penicillin, 100 μg/ml streptomycin [STEMCELL Technologies]) and 10^{-4} M β-mercaptoethanol (Sigma-Aldrich). The presence of single cells was then confirmed by visual inspection. A second 100 μl of medium was added for cultures initiated with 30 ESLAM cells. The following additives were used as indicated: mouse SF and IL-3, and human Epo and Col 1 purchased from STEMCELL Technologies; human IL-11 and macrophage colony stimulating factor (M-CSF) obtained as gifts from Genetics Institute; mouse Wnt3a, Angptl3, and IGFBP2, and human NGF, pleiotrophin, bone morphogenetic protein 4 (BMP4), Activin A, transforming growth factor β (TGF-β), and platelet-derived growth factor (PDGF) BB purchased from R&D Systems; mouse TPO obtained as a gift from Genentech; human FGF-1 purchased from Invitrogen; heparin, fibronectin, human epidermal growth factor, and mouse laminin purchased from Sigma-Aldrich; human IL-6 obtained as a gift from Cangene; reduced growth factor (RGF) Matrigel purchased from BD; and SDF-1 obtained as a gift from Dr. I. Clark Lewis (University of British Columbia).

For in vivo and LTC-IC assessment of the first division progeny of single ESLAM cells, cultures were examined microscopically every 4 hr starting 32 hr after initiation of the culture. Thereafter, the entire volume in each well found to have produced two cells in the previous 4 hr was distributed into three or more wells to obtain both daughters in different wells so they could then be transplanted separately into two different mice or used to initiate two separate LTCs. If only one cell was recovered, the remaining cell was discarded. To track the kinetics of cell division and viability, cultures were monitored starting 12 hr after initiation and thereafter as indicated. The first appearance of two refractile cells was used to indicate completion of a first division. Following the addition of 15% FBS, 50 ng/ml SF, 10 ng/ml IL-3, 10 ng/ml IL-6, and 3 U/ml Epo to each well after 4½ days of incubation, evidence of viability was inferred from the detection of a clone of seven or more refractile cells. To measure apoptotic cells, cells were harvested after 36 hr, washed, resuspended in Binding Buffer, stained with Annexin V eFluor 450 and FITC-conjugated anti-CD45 (all from eBioscience), and analyzed on a BD LSR Fortessa.

LTC-IC Assays

Visually confirmed single cells were added onto irradiated UG26 feeder cells in flat-bottomed wells containing 200 μl of MyeloCult (STEMCELL Technologies) supplemented with 10^{-6} M hydrocortisone (Sigma-Aldrich) in 96-well plates and cultures maintained with weekly half-medium changes (Woehrer et al., 2013). After 6 weeks, fresh SFM containing FBS plus SF plus IL-3 plus IL-6 plus Epo was added to the wells, and those containing clusters of >25 nonadherent cells 12 days later were counted as positive.

Transcriptome Analyses

Between 2,000 (cultured cells) and 6,000 (freshly isolated adult BM) ESLAM cells were collected, and mRNA was extracted with RNeasy (QIAGEN). mRNA from three independent experiments was pooled, reverse transcribed, and amplified with Nanokit following the manufacturer's instructions (Agilent

Technologies). cRNA was hybridized onto two gene chips (GeneChip Mouse Gene 1.0 ST Array; Affymetrix) per condition (GSE57220, http://www.ncbi.nlm.nih.gov/geo/query/acc.cgi?acc=GSE57220). Data analysis was performed as detailed in Supplemental Experimental Procedures.

Statistical Analysis

GraphPad Prism version 5 or R (http://www.R-project.org/) was used to perform basic statistical analyses, including calculation of mean ± SEM values and to perform Student's t tests. ELDA: Extreme Limiting Dilution Analysis (http://bioinf.wehi.edu.au/software/elda/) or the "elda" function in the R package, "statmod," was used to perform the limiting dilution analyses and to evaluate the significance of differences obtained using different culture conditions.

ACCESSION NUMBERS

The GEO accession number for the transcriptome data reported in this paper is GSE57220.

SUPPLEMENTAL INFORMATION

Supplemental Information includes Supplemental Experimental Procedures, one figure, and three tables and can be found with this article online at http://dx.doi.org/10.1016/j.celrep.2014.05.014.

AUTHOR CONTRIBUTIONS

S.W., D.J.H.F.K., and C.J.E. conceived and oversaw the design and execution of the experiments. S.W., D.J.H.F.K., M.R.C., C.B., D.G.K., S.B., K.R., and H.M. collected the data. S.W., D.J.H.F.K., M.R.C., C.B., D.G.K., and S.B. contributed to the interpretation of the data. S.W., D.J.H.F.K., and C.J.E. wrote the manuscript, and all authors approved it.

ACKNOWLEDGMENTS

We thank the staff of the Flow Cytometry Facility of the Terry Fox Laboratory and the Animal Resource Centre of the BC Cancer Agency. This work was supported by grants from the National Cancer Institute of Canada (Toronto, ON), with funds from the Terry Fox Run, and the Canadian Institutes of Health Research (CIHR, Toronto, ON). S.W. received an Erwin Schroedinger Fellowship (J2684) as well as an FWF grant (P25134) from the Austrian Science Fund (Vienna). D.J.H.F.K. received studentships from CIHR including a Vanier Studentship. C.B. received a fellowship from the Deutsche Forschungsgemeinschaft (Bonn). M.R.C. and D.G.K. received studentships from the Michael Smith Foundation for Health Research and CIHR. S.B. received studentships from the University of British Columbia and CIHR.

Received: January 12, 2014
Revised: February 2, 2014
Accepted: May 6, 2014
Published: June 5, 2014

REFERENCES

Audet, J., Miller, C.L., Eaves, C.J., and Piret, J.M. (2002). Common and distinct features of cytokine effects on hematopoietic stem and progenitor cells revealed by dose-response surface analysis. Biotechnol. Bioeng. *80*, 393–404.

Benveniste, P., Frelin, C., Janmohamed, S., Barbara, M., Herrington, R., Hyam, D., and Iscove, N.N. (2010). Intermediate-term hematopoietic stem cells with extended but time-limited reconstitution potential. Cell Stem Cell *6*, 48–58.

Benz, C., Copley, M.R., Kent, D.G., Wohrer, S., Cortes, A., Aghaeepour, N., Ma, E., Mader, H., Rowe, K., Day, C., et al. (2012). Hematopoietic stem cell subtypes expand differentially during development and display distinct lymphopoietic programs. Cell Stem Cell *10*, 273–283.

Bowie, M.B., McKnight, K.D., Kent, D.G., McCaffrey, L., Hoodless, P.A., and Eaves, C.J. (2006). Hematopoietic stem cells proliferate until after birth and show a reversible phase-specific engraftment defect. J. Clin. Invest. *116*, 2808–2816.

Celebi, B., Mantovani, D., and Pineault, N. (2011). Effects of extracellular matrix proteins on the growth of haematopoietic progenitor cells. Biomed. Mater. *6*, 055011.

Colvin, G.A., Lambert, J.F., Abedi, M., Hsieh, C.C., Carlson, J.E., Stewart, F.M., and Quesenberry, P.J. (2004). Murine marrow cellularity and the concept of stem cell competition: geographic and quantitative determinants in stem cell biology. Leukemia *18*, 575–583.

Dexter, T.M., Allen, T.D., and Lajtha, L.G. (1977). Conditions controlling the proliferation of haemopoietic stem cells in vitro. J. Cell. Physiol. *91*, 335–344.

Ding, L., and Morrison, S.J. (2013). Haematopoietic stem cells and early lymphoid progenitors occupy distinct bone marrow niches. Nature *495*, 231–235.

Domen, J., Cheshier, S.H., and Weissman, I.L. (2000). The role of apoptosis in the regulation of hematopoietic stem cells: overexpression of Bcl-2 increases both their number and repopulation potential. J. Exp. Med. *191*, 253–264.

Dykstra, B., Ramunas, J., Kent, D., McCaffrey, L., Szumsky, E., Kelly, L., Farn, K., Blaylock, A., Eaves, C., and Jervis, E. (2006). High-resolution video monitoring of hematopoietic stem cells cultured in single-cell arrays identifies new features of self-renewal. Proc. Natl. Acad. Sci. USA *103*, 8185–8190.

Dykstra, B., Kent, D., Bowie, M., McCaffrey, L., Hamilton, M., Lyons, K., Lee, S.J., Brinkman, R., and Eaves, C. (2007). Long-term propagation of distinct hematopoietic differentiation programs in vivo. Cell Stem Cell *1*, 218–229.

Fraser, C.C., Eaves, C.J., Szilvassy, S.J., and Humphries, R.K. (1990). Expansion in vitro of retrovirally marked totipotent hematopoietic stem cells. Blood *76*, 1071–1076.

Fraser, C.C., Szilvassy, S.J., Eaves, C.J., and Humphries, R.K. (1992). Proliferation of totipotent hematopoietic stem cells in vitro with retention of long-term competitive in vivo reconstituting ability. Proc. Natl. Acad. Sci. USA *89*, 1968–1972.

García, R., Aguiar, J., Alberti, E., de la Cuétara, K., and Pavón, N. (2004). Bone marrow stromal cells produce nerve growth factor and glial cell line-derived neurotrophic factors. Biochem. Biophys. Res. Commun. *316*, 753–754.

Guezguez, B., Campbell, C.J., Boyd, A.L., Karanu, F., Casado, F.L., Di Cresce, C., Collins, T.J., Shapovalova, Z., Xenocostas, A., and Bhatia, M. (2013). Regional localization within the bone marrow influences the functional capacity of human HSCs. Cell Stem Cell *13*, 175–189.

Hu, G., Xu, J.J., Deng, Z.H., Feng, J., and Jin, Y. (2011). Supernatant of bone marrow mesenchymal stromal cells induces peripheral blood mononuclear cells possessing mesenchymal features. Int. J. Biol. Sci. *7*, 364–375.

Huynh, H., Iizuka, S., Kaba, M., Kirak, O., Zheng, J., Lodish, H.F., and Zhang, C.C. (2008). Insulin-like growth factor-binding protein 2 secreted by a tumorigenic cell line supports ex vivo expansion of mouse hematopoietic stem cells. Stem Cells *26*, 1628–1635.

Jordan, C.T., and Guzman, M.L. (2004). Mechanisms controlling pathogenesis and survival of leukemic stem cells. Oncogene *23*, 7178–7187.

Kent, D.G., Dykstra, B.J., Cheyne, J., Ma, E., and Eaves, C.J. (2008). Steel factor coordinately regulates the molecular signature and biologic function of hematopoietic stem cells. Blood *112*, 560–567.

Kent, D.G., Copley, M.R., Benz, C., Wöhrer, S., Dykstra, B.J., Ma, E., Cheyne, J., Zhao, Y., Bowie, M.B., Zhao, Y., et al. (2009). Prospective isolation and molecular characterization of hematopoietic stem cells with durable self-renewal potential. Blood *113*, 6342–6350.

Kunisaki, Y., Bruns, I., Scheiermann, C., Ahmed, J., Pinho, S., Zhang, D., Mizoguchi, T., Wei, Q., Lucas, D., Ito, K., et al. (2013). Arteriolar niches maintain haematopoietic stem cell quiescence. Nature *502*, 637–643.

Kurth, I., Franke, K., Pompe, T., Bornhäuser, M., and Werner, C. (2011). Extracellular matrix functionalized microcavities to control hematopoietic stem and progenitor cell fate. Macromol. Biosci. *11*, 739–747.

Lecault, V., Vaninsberghe, M., Sekulovic, S., Knapp, D.J., Wohrer, S., Bowden, W., Viel, F., McLaughlin, T., Jarandehei, A., Miller, M., et al. (2011).

High-throughput analysis of single hematopoietic stem cell proliferation in microfluidic cell culture arrays. Nat. Methods *8*, 581–586.

Ledran, M.H., Krassowska, A., Armstrong, L., Dimmick, I., Renström, J., Lang, R., Yung, S., Santibanez-Coref, M., Dzierzak, E., Stojkovic, M., et al. (2008). Efficient hematopoietic differentiation of human embryonic stem cells on stromal cells derived from hematopoietic niches. Cell Stem Cell *3*, 85–98.

Lutolf, M.P., Doyonnas, R., Havenstrite, K., Koleckar, K., and Blau, H.M. (2009). Perturbation of single hematopoietic stem cell fates in artificial niches. Integr. Biol. (Camb) *1*, 59–69.

McCulloch, E.A., Siminovitch, L., Till, J.E., Russell, E.S., and Bernstein, S.E. (1965). The cellular basis of the genetically determined hemopoietic defect in anemic mice of genotype Sl-Sld. Blood *26*, 399–410.

Mercier, F.E., Ragu, C., and Scadden, D.T. (2012). The bone marrow at the crossroads of blood and immunity. Nat. Rev. Immunol. *12*, 49–60.

Miller, C.L., and Eaves, C.J. (1997). Expansion in vitro of adult murine hematopoietic stem cells with transplantable lympho-myeloid reconstituting ability. Proc. Natl. Acad. Sci. USA *94*, 13648–13653.

Moore, K.A., Ema, H., and Lemischka, I.R. (1997). In vitro maintenance of highly purified, transplantable hematopoietic stem cells. Blood *89*, 4337–4347.

Morita, Y., Ema, H., and Nakauchi, H. (2010). Heterogeneity and hierarchy within the most primitive hematopoietic stem cell compartment. J. Exp. Med. *207*, 1173–1182.

Notta, F., Doulatov, S., Laurenti, E., Poeppl, A., Jurisica, I., and Dick, J.E. (2011). Isolation of single human hematopoietic stem cells capable of long-term multilineage engraftment. Science *333*, 218–221.

Oostendorp, R.A., Harvey, K.N., Kusadasi, N., de Bruijn, M.F., Saris, C., Ploemacher, R.E., Medvinsky, A.L., and Dzierzak, E.A. (2002). Stromal cell lines from mouse aorta-gonads-mesonephros subregions are potent supporters of hematopoietic stem cell activity. Blood *99*, 1183–1189.

Oostendorp, R.A., Robin, C., Steinhoff, C., Marz, S., Bräuer, R., Nuber, U.A., Dzierzak, E.A., and Peschel, C. (2005). Long-term maintenance of hematopoietic stem cells does not require contact with embryo-derived stromal cells in cocultures. Stem Cells *23*, 842–851.

Ploemacher, R.E., van der Sluijs, J.P., Voerman, J.S., and Brons, N.H. (1989). An in vitro limiting-dilution assay of long-term repopulating hematopoietic stem cells in the mouse. Blood *74*, 2755–2763.

Reya, T., Duncan, A.W., Ailles, L., Domen, J., Scherer, D.C., Willert, K., Hintz, L., Nusse, R., and Weissman, I.L. (2003). A role for Wnt signalling in self-renewal of haematopoietic stem cells. Nature *423*, 409–414.

Sanjuan-Pla, A., Macaulay, I.C., Jensen, C.T., Woll, P.S., Luis, T.C., Mead, A., Moore, S., Carella, C., Matsuoka, S., Bouriez Jones, T., et al. (2013). Platelet-biased stem cells reside at the apex of the haematopoietic stem-cell hierarchy. Nature *502*, 232–236.

Sutherland, D.J., Till, J.E., and McCulloch, E.A. (1970). A kinetic study of the genetic control of hemopoietic progenitor cells assayed in culture and in vivo. J. Cell. Physiol. *75*, 267–274.

Umemoto, T., Yamato, M., Ishihara, J., Shiratsuchi, Y., Utsumi, M., Morita, Y., Tsukui, H., Terasawa, M., Shibata, T., Nishida, K., et al. (2012). Integrin-$\alpha v\beta 3$ regulates thrombopoietin-mediated maintenance of hematopoietic stem cells. Blood *119*, 83–94.

Wagers, A.J., and Weissman, I.L. (2006). Differential expression of alpha2 integrin separates long-term and short-term reconstituting Lin-/loThy1.1(lo) c-kit+ Sca-1+ hematopoietic stem cells. Stem Cells *24*, 1087–1094.

Wineman, J., Moore, K., Lemischka, I., and Müller-Sieburg, C. (1996). Functional heterogeneity of the hematopoietic microenvironment: rare stromal elements maintain long-term repopulating stem cells. Blood *87*, 4082–4090.

Woehrer, S., Miller, C.L., and Eaves, C.J. (2013). Long-term culture-initiating cell assay for mouse cells. Methods Mol. Biol. *946*, 257–266.

Xie, H., Xu, J., Hsu, J.H., Nguyen, M., Fujiwara, Y., Peng, C., and Orkin, S.H. (2014). Polycomb repressive complex 2 regulates normal hematopoietic stem cell function in a developmental-stage-specific manner. Cell Stem Cell *14*, 68–80.

Yamamoto, R., Morita, Y., Ooehara, J., Hamanaka, S., Onodera, M., Rudolph, K.L., Ema, H., and Nakauchi, H. (2013). Clonal analysis unveils self-renewing lineage-restricted progenitors generated directly from hematopoietic stem cells. Cell *154*, 1112–1126.

Yamazaki, S., Iwama, A., Takayanagi, S., Eto, K., Ema, H., and Nakauchi, H. (2009). TGF-beta as a candidate bone marrow niche signal to induce hematopoietic stem cell hibernation. Blood *113*, 1250–1256.

Zhang, C.C., Kaba, M., Ge, G., Xie, K., Tong, W., Hug, C., and Lodish, H.F. (2006). Angiopoietin-like proteins stimulate ex vivo expansion of hematopoietic stem cells. Nat. Med. *12*, 240–245.

SUPPLEMENTAL MATERIAL

Supplemental Methods - HSC transplantation assays

Cells were transplanted by tail vein injection into sublethally irradiated Ly5-congenic *W41* mice and PB follow-up analyses of donor contributions to the B, T and GM lineages were then performed 8, 16, and 24 weeks later as previously described (Kent et al., 2007) using both anti–CD45.1-APC (eBiosciences) and anti–CD45.2-FITC (BD) to enable donor and recipient cells to be distinguished and to ensure that any double positive CD45.1 and CD45.2 events were excluded. Staining with anti–Ly6g-PE and anti–Mac1-PE was used to detect GM cells, anti–CD19-PE for B-cells, and anti–CD5-PE for T-cells (all antibodies from BD). Secondary transplantations were performed 24 weeks after the primary transplantations. The subtype of the original HSC(s) transplanted was first determined 16 weeks post transplantation and reconfirmed either within one week before or on the day cells were harvested to perform secondary transplantations. For the latter, BM cells were harvested from both femurs, tibiae and the pelvis of primary mice, red blood cells lysed, and 10^7 cells then injected into each secondary recipient. HSC frequency values were calculated from limiting dilution transplant results using an on-line program (http://bioinf.wehi.edu.au/software/elda/) and a positive read-out of >1% contribution to the total PB white blood cell pool at 16 weeks. HSCs were subclassified as DSR-HSCs when they produced >1% of the PB GM cells and as LSR-HSCs when they contributed <1% of the GM cells 16 weeks after transplantation. Differentiation patterns of HSCs were subclassified as α, β, γ, and δ based on the following ratios of their contributions to the total PB GM vs (T+B)-cell pools in recipient mice assessed 16-24 weeks post-transplant (i.e., >2.0; 0.25-2.0, <0.25 with >1% contribution to the myeloid lineage, and <0.25 with <1% contribution to the myeloid lineage, respectively), as described previously (Dykstra et al., 2007).

Microarray Analysis

All arrays were robust multi-array averaged (RMA) normalized using the Bioconductor package 'xps' in R (http://www.R-project.org/) including the metacore probesets grouped by exon (Gentleman et al., 2004). Gene annotation data was added using a combination of the NetAffx (Release 31) annotation files as well as the Bioconductor packages 'mogene10sttranscriptcluster.db' and 'Org.Mm.eg.db'. All probe sets that were not mapped to an Entrez Gene identifier were discarded. Data Above Background (DAB) scores were calculated for each probe set and those probe sets with a DAB p-value ≤0.05 in both technical replicates of at least one condition were retained. In the case of multiple probe sets mapping to the same Entrez Gene identifier, the probe set with the highest maximum absolute deviation across all chips was retained. This was done in order to maximize power in later statistical tests as it reduces multiple testing while retaining the probes with the greatest signal. Expression values were then normalized between the arrays by quantile normalization (Smyth et al., 2005) using the R package 'limma'.

Publicly available microarray data for the UG26-1B6 cell line (Gene Expression Omnibus, GSE11589) was used together with the data for the freshly isolated ESLAM cells (from above) in order to generate a list of potential secreted factors. This data (from the mouse 4302 chip) was RMA-normalized and transcripts called as present if at least one probe set had a MAS5 p-value of 0.05 or less using the R package ‘affy’. Transcripts that were defined as present and that also had the gene ontology (GO) term “extracellular region” (GO:0005576) were compared to transcripts with the gene ontology term “receptor activity” (GO:0004872) present in transcripts of fresh ESLAM cells (as defined above). “Extracellular region” transcripts from UG26 which had transcripts with “receptor activity” in fresh ESLAM cells predicted or

shown to interact in either the National Institute of Aging (NIA) mouse protein-protein interaction database (Yellaboina et al., 2008), the BioGRID database (Stark et al., 2006) or the Cytokine-cytokine receptor interaction (mmu04060) or ECM-receptor interaction (mmu04512) entries from the Kyoto Encyclopedia of Genes and Genomes (KEGG) Pathway database (www.genome.jp/kegg/pathway.html) were retained as potential secreted factors, and their median expression and putative receptors (interacting "receptor activity" transcripts) recorded.

For pathway analysis, arrays from extracts of cells exposed to CM were grouped and compared against extracts from fresh ESLAM cells and from cells exposed to SF+IL-11 only in order to provide biological replication and minimize differences due only to differential cell cycle status. The 'romer' algorithm (a rotation based modification of the GSEA algorithm (Subramanian et al., 2005)) was used to test for differential expression of genes in pathways from the REACTOME database (Croft et al.) (taken from the R version of the molecular signatures database, http://bioinf.wehi.edu.au/software/MSigDB/) using a mixed alternative hypothesis (i.e., the differential expression can be in both directions) with the "floor mean" test statistic and 100,000 rotations, correcting for correlation between technical replicates (Smyth et al., 2005).

Supplemental Figure

Figure S1. Comparison of the serial GM-, B-, and T-cell reconstituting activities of the 2 β-HSCs and their progeny produced from the first *in vitro* division of a single ESLAM cell. Donor-derived contributions to the PB GM cells are shown in red, to the PB B-cells in blue, and to the PB T-cells in black.

Supplementary Figure 1

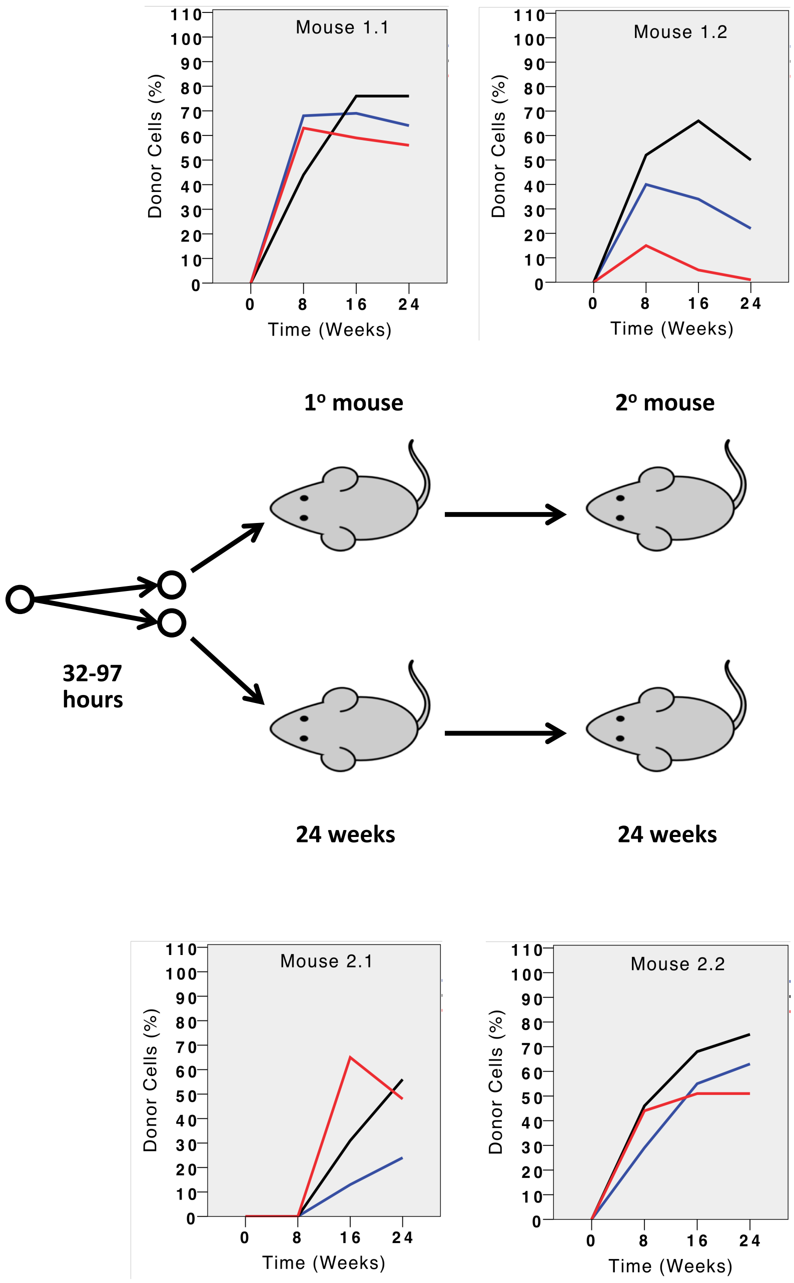

Supplemental Tables

Table S1: REACTOME pathways (n=250) with significantly altered transcript expression in ESLAM cells maintained in S+11+UG26 CM.

**Pathway**	**Gene No.**	**P value**
ADP SIGNALLING THROUGH P2Y PURINOCEPTOR 1	25	9.9999E-06
APOPTOSIS	122	9.9999E-06
AUTODEGRADATION OF CDH1 BY CDH1 APC	53	9.9999E-06
AXON GUIDANCE	156	9.9999E-06
BRANCHED CHAIN AMINO ACID CATABOLISM	17	9.9999E-06
CD28 DEPENDENT VAV1 PATHWAY	11	9.9999E-06
CDC20 PHOSPHO APC MEDIATED DEGRADATION OF CYCLIN A	59	9.9999E-06
CDT1 ASSOCIATION WITH THE CDC6 ORC ORIGIN COMPLEX	48	9.9999E-06
CELL CYCLE CHECKPOINTS	104	9.9999E-06
CELL CYCLE MITOTIC	292	9.9999E-06
CELL SURFACE INTERACTIONS AT THE VASCULAR WALL	89	9.9999E-06
CENTROSOME MATURATION	67	9.9999E-06
CITRIC ACID CYCLE	18	9.9999E-06
CLATHRIN DERIVED VESICLE BUDDING	56	9.9999E-06
COSTIMULATION BY THE CD28 FAMILY	60	9.9999E-06
CYCLIN E ASSOCIATED EVENTS DURING G1 S TRANSITION	54	9.9999E-06
DARPP32 EVENTS	25	9.9999E-06
DIABETES PATHWAYS	292	9.9999E-06
DNA REPAIR	98	9.9999E-06
DNA REPLICATION PRE INITIATION	71	9.9999E-06
DOUBLE STRAND BREAK REPAIR	21	9.9999E-06
DOWN STREAM SIGNAL TRANSDUCTION	35	9.9999E-06
DOWNSTREAM TCR SIGNALING	36	9.9999E-06
ELECTRON TRANSPORT CHAIN	56	9.9999E-06
ELONGATION AND PROCESSING OF CAPPED TRANSCRIPTS	109	9.9999E-06
FORMATION AND MATURATION OF MRNA TRANSCRIPT	127	9.9999E-06
FORMATION OF A POOL OF FREE 40S SUBUNITS	40	9.9999E-06
FORMATION OF ATP BY CHEMIOSMOTIC COUPLING	10	9.9999E-06
FORMATION OF PLATELET PLUG	180	9.9999E-06
FORMATION OF THE TERNARY COMPLEX AND SUBSEQUENTLY THE 43S COMPLEX	27	9.9999E-06
FURTHER PLATELET RELEASATE	22	9.9999E-06
G1 S TRANSITION	95	9.9999E-06
G2 M TRANSITION	79	9.9999E-06
GENE EXPRESSION	341	9.9999E-06
GENERIC TRANSCRIPTION PATHWAY	35	9.9999E-06
GLOBAL GENOMIC NER	32	9.9999E-06
GLUCONEOGENESIS	27	9.9999E-06
GLUCOSE METABOLISM	51	9.9999E-06
GLUCOSE REGULATION OF INSULIN SECRETION	130	9.9999E-06

GLYCOGEN BREAKDOWN GLYCOGENOLYSIS	15	9.9999E-06
GLYCOLYSIS	19	9.9999E-06
GOLGI ASSOCIATED VESICLE BIOGENESIS	50	9.9999E-06
GRB2 SOS PROVIDES LINKAGE TO MAPK SIGNALING FOR INTERGRINS	15	9.9999E-06
GTP HYDROLYSIS AND JOINING OF THE 60S RIBOSOMAL SUBUNIT	50	9.9999E-06
HEMOSTASIS	264	9.9999E-06
HIV INFECTION	169	9.9999E-06
HIV LIFE CYCLE	98	9.9999E-06
HIV1 TRANSCRIPTION INITIATION	38	9.9999E-06
HOST INTERACTIONS OF HIV FACTORS	109	9.9999E-06
INFLUENZA LIFE CYCLE	80	9.9999E-06
INFLUENZA VIRAL RNA TRANSCRIPTION AND REPLICATION	44	9.9999E-06
INSULIN SYNTHESIS AND SECRETION	72	9.9999E-06
INTEGRATION OF ENERGY METABOLISM	198	9.9999E-06
INTEGRIN CELL SURFACE INTERACTIONS	80	9.9999E-06
LATE PHASE OF HIV LIFE CYCLE	87	9.9999E-06
LOSS OF NLP FROM MITOTIC CENTROSOMES	60	9.9999E-06
M G1 TRANSITION	57	9.9999E-06
MEMBRANE TRAFFICKING	73	9.9999E-06
METABLISM OF NUCLEOTIDES	70	9.9999E-06
METABOLISM OF CARBOHYDRATES	109	9.9999E-06
METABOLISM OF PROTEINS	153	9.9999E-06
MITOTIC PROMETAPHASE	89	9.9999E-06
MRNA 3 END PROCESSING	22	9.9999E-06
MRNA PROCESSING	31	9.9999E-06
MRNA SPLICING	83	9.9999E-06
MRNA SPLICING MINOR PATHWAY	32	9.9999E-06
NCAM SIGNALING FOR NEURITE OUT GROWTH	67	9.9999E-06
NCAM1 INTERACTIONS	43	9.9999E-06
NUCLEAR IMPORT OF REV PROTEIN	29	9.9999E-06
NUCLEOTIDE EXCISION REPAIR	47	9.9999E-06
OPIOID SIGNALLING	82	9.9999E-06
ORC1 REMOVAL FROM CHROMATIN	59	9.9999E-06
OTHER SEMAPHORIN INTERACTIONS	15	9.9999E-06
P38MAPK EVENTS	13	9.9999E-06
P53 INDEPENDENT DNA DAMAGE RESPONSE	39	9.9999E-06
PEPTIDE CHAIN ELONGATION	31	9.9999E-06
PLATELET ACTIVATION	161	9.9999E-06
PLATELET ACTIVATION TRIGGERS	58	9.9999E-06
PLATELET DEGRANULATION	82	9.9999E-06
PREFOLDIN MEDIATED TRANSFER OF SUBSTRATE TO CCT TRIC	24	9.9999E-06
PROCESSING OF CAPPED INTRON CONTAINING PRE MRNA	113	9.9999E-06
PYRUVATE METABOLISM AND TCA CYCLE	35	9.9999E-06
REGULATION OF APC ACTIVATORS BETWEEN G1 S AND EARLY ANAPHASE	66	9.9999E-06
REGULATION OF BETA CELL DEVELOPMENT	62	9.9999E-06
REGULATION OF GENE EXPRESSION IN BETA CELLS	48	9.9999E-06
REGULATION OF INSULIN SECRETION	180	9.9999E-06
REGULATION OF INSULIN SECRETION BY GLUCAGON LIKE PEPTIDE 1	59	9.9999E-06
REGULATION OF ORNITHINE DECARBOXYLASE	43	9.9999E-06
REV MEDIATED NUCLEAR EXPORT OF HIV1 RNA	30	9.9999E-06

RHO GTPASE CYCLE	120	9.9999E-06
RNA POL II CTD PHOSPHORYLATION AND INTERACTION WITH CE	25	9.9999E-06
RNA POLYMERASE I CHAIN ELONGATION	20	9.9999E-06
RNA POLYMERASE I PROMOTER ESCAPE	20	9.9999E-06
RNA POLYMERASE I TRANSCRIPTION INITIATION	24	9.9999E-06
RNA POLYMERASE I TRANSCRIPTION TERMINATION	21	9.9999E-06
RNA POLYMERASE II TRANSCRIPTION	78	9.9999E-06
S PHASE	98	9.9999E-06
SCF BETA TRCP MEDIATED DEGRADATION OF EMI1	44	9.9999E-06
SCF SKP2 MEDIATED DEGRADATION OF P27 P21	48	9.9999E-06
SEMA3A PAK DEPENDENT AXON REPULSION	13	9.9999E-06
SEMA4D IN SEMAPHORIN SIGNALING	28	9.9999E-06
SEMA4D INDUCED CELL MIGRATION AND GROWTH CONE COLLAPSE	23	9.9999E-06
SEMAPHORIN INTERACTIONS	63	9.9999E-06
SHC MEDIATED SIGNALLING	12	9.9999E-06
SIGNALING BY PDGF	63	9.9999E-06
SIGNALING BY TGF BETA	15	9.9999E-06
SIGNALING BY WNT	54	9.9999E-06
SIGNALING IN IMMUNE SYSTEM	294	9.9999E-06
SIGNALLING BY NGF	208	9.9999E-06
SIGNALLING TO ERKS	34	9.9999E-06
SIGNALLING TO RAS	26	9.9999E-06
SNRNP ASSEMBLY	45	9.9999E-06
SOS MEDIATED SIGNALLING	13	9.9999E-06
SYNTHESIS OF DNA	84	9.9999E-06
TCR SIGNALING	53	9.9999E-06
TIE2 SIGNALING	18	9.9999E-06
TRAF6 MEDIATED INDUCTION OF THE ANTIVIRAL CYTOKINE IFN ALPHA BETA CASCADE	52	9.9999E-06
TRANSCRIPTION	146	9.9999E-06
TRANSCRIPTION OF THE HIV GENOME	57	9.9999E-06
TRANSLATION	64	9.9999E-06
TRANSLATION INITIATION COMPLEX FORMATION	33	9.9999E-06
TRANSMISSION ACROSS CHEMICAL SYNAPSES	127	9.9999E-06
TRANSPORT OF MATURE MRNA DERIVED FROM AN INTRON CONTAINING TRANSCRIPT	39	9.9999E-06
TRKA SIGNALLING FROM THE PLASMA MEMBRANE	102	9.9999E-06
VIF MEDIATED DEGRADATION OF APOBEC3G	42	9.9999E-06
VIRAL MRNA TRANSLATION	30	9.9999E-06
VPR MEDIATED NUCLEAR IMPORT OF PICS	30	9.9999E-06
PP2A MEDIATED DEPHOSPHORYLATION OF KEY METABOLIC FACTORS	10	9.9999E-06
SYNTHESIS AND INTERCONVERSION OF NUCLEOTIDE DI AND TRIPHOSPHATES	18	9.9999E-06
TRANSPORT OF RIBONUCLEOPROTEINS INTO THE HOST NUCLEUS	28	9.9999E-06
GLUCOSE TRANSPORT	37	9.9999E-06
MRNA DECAY BY 5 TO 3 EXORIBONUCLEASE	12	9.9999E-06
REGULATION OF GLUCOKINASE BY GLUCOKINASE REGULATORY PROTEIN	28	9.9999E-06
STABILIZATION OF P53	41	9.9999E-06
DEPOLARIZATION OF THE PRESYNAPTIC TERMINAL TRIGGERS THE	12	9.9999E-06

OPENING OF CALCIUM CHANNELS		
INHIBITION OF INSULIN SECRETION BY ADRENALINE NORADRENALINE	30	9.9999E-06
MRNA DECAY BY 3 TO 5 EXORIBONUCLEASE	10	9.9999E-06
NEP NS2 INTERACTS WITH THE CELLULAR EXPORT MACHINERY	28	9.9999E-06
RNA POLYMERASE I III AND MITOCHONDRIAL TRANSCRIPTION	82	9.9999E-06
TRANSPORT OF THE SLBP INDEPENDENT MATURE MRNA	31	9.9999E-06
SMOOTH MUSCLE CONTRACTION	23	9.9999E-06
METABOLISM OF MRNA	42	9.9999E-06
METABOLISM OF RNA	87	9.9999E-06
MITOTIC M M G1 PHASES	150	9.9999E-06
APCDC20 MEDIATED DEGRADATION OF CYCLIN B	16	1.99998E-05
CHAPERONIN MEDIATED PROTEIN FOLDING	45	1.99998E-05
FORMATION OF TUBULIN FOLDING INTERMEDIATES BY CCT TRIC	17	1.99998E-05
INACTIVATION OF APC VIA DIRECT INHIBITION OF THE APCOMPLEX	18	1.99998E-05
GRB2 EVENTS IN EGFR SIGNALING	13	2.99997E-05
HIV1 TRANSCRIPTION ELONGATION	39	2.99997E-05
P75 NTR RECEPTOR MEDIATED SIGNALLING	78	2.99997E-05
RNA POLYMERASE I PROMOTER CLEARANCE	49	2.99997E-05
SEMA3A PLEXIN REPULSION SIGNALING BY INHIBITING INTEGRIN ADHESION	13	2.99997E-05
DUAL INCISION REACTION IN GG NER	20	3.99996E-05
SIGNAL AMPLIFICATION	31	5.99994E-05
LYSOSOME VESICLE BIOGENESIS	22	6.99993E-05
MTOR SIGNALLING	26	6.99993E-05
MUSCLE CONTRACTION	50	8.99991E-05
MAP KINASES ACTIVATION IN TLR CASCADE	44	0.000109999
METABOLISM OF AMINO ACIDS	152	0.000119999
TRANSCRIPTION COUPLED NER	42	0.000129999
PHOSPHORYLATION OF THE APC	16	0.000149999
DUAL INCISION REACTION IN TC NER	27	0.000159998
RNA POLYMERASE III TRANSCRIPTION TERMINATION	16	0.000159998
TOLL RECEPTOR CASCADES	83	0.000209998
G ALPHA 12 13 SIGNALLING EVENTS	54	0.000259997
HORMONE BIOSYNTHESIS	48	0.000289997
TOLL LIKE RECEPTOR 3 CASCADE	58	0.000339997
EXTENSION OF TELOMERES	26	0.000369996
CONVERSION FROM APC CDC20 TO APC CDH1 IN LATE ANAPHASE	17	0.000419996
CYCLIN A1 ASSOCIATED EVENTS DURING G2 M TRANSITION	14	0.000419996
MYOGENESSIS	28	0.000419996
CRMPS IN SEMA3A SIGNALING	15	0.000689993
ENERGY DEPENDENT REGULATION OF MTOR BY LKB1 AMPK	17	0.000689993
PURINE RIBONUCLEOSIDE MONOPHOSPHATE BIOSYNTHESIS	11	0.000729993
REGULATION OF AMPK ACTIVITY VIA LKB1	14	0.000949991
SIGNALLING TO P38 VIA RIT AND RIN	14	0.00099999
SHC RELATED EVENTS	14	0.00104999
ACTIVATION OF ATR IN RESPONSE TO REPLICATION STRESS	37	0.001199988
NEURORANSMITTER RECEPTOR BINDING AND DOWNSTREAM TRANSMISSION IN THE POSTSYNAPTIC CELL	81	0.001329987
P130CAS LINKAGE TO MAPK SIGNALING FOR INTEGRINS	15	0.001429986
IRS RELATED EVENTS	78	0.001439986

COLLAGEN MEDIATED ACTIVATION CASCADE	22	0.001879981
PD1 SIGNALING	19	0.002419976
DNA STRAND ELONGATION	30	0.002529975
ACTIVATION OF NMDA RECEPTOR UPON GLUTAMATE BINDING AND POSTSYNAPTIC EVENTS	35	0.002779972
POST NMDA RECEPTOR ACTIVATION EVENTS	31	0.00297997
ACTIVATION OF KAINATE RECEPTORS UPON GLUTAMATE BINDING	32	0.003079969
UNFOLDED PROTEIN RESPONSE	18	0.003359966
COPI MEDIATED TRANSPORT	10	0.003579964
IONOTROPIC ACTIVITY OF KAINATE RECEPTORS	12	0.003679963
G BETA GAMMA SIGNALLING THROUGH PI3KGAMMA	25	0.004239958
ASSOCIATION OF TRIC CCT WITH TARGET PROTEINS DURING BIOSYNTHESIS	29	0.004539955
FRS2 MEDIATED ACTIVATION	16	0.004679953
SIGNALING BY EGFR	46	0.004809952
MAPK TARGETS NUCLEAR EVENTS MEDIATED BY MAP KINASES	30	0.005239948
FANCONI ANEMIA PATHWAY	14	0.005269947
NOREPINEPHRINE NEUROTRANSMITTER RELEASE CYCLE	12	0.005859941
UNWINDING OF DNA	11	0.006569934
G PROTEIN ACTIVATION	28	0.007599924
HOMOLOGOUS RECOMBINATION REPAIR	15	0.007729923
POST CHAPERONIN TUBULIN FOLDING PATHWAY	14	0.007929921
INNATE IMMUNITY SIGNALING	102	0.008489915
FORMATION OF THE EARLY ELONGATION COMPLEX	30	0.008569914
ERKS ARE INACTIVATED	12	0.008609914
G PROTEIN BETA GAMMA SIGNALLING	28	0.00895991
THROMBOXANE SIGNALLING THROUGH TP RECEPTOR	23	0.009259907
CYTOSOLIC TRNA AMINOACYLATION	23	0.009319907
THROMBIN SIGNALLING THROUGH PROTEINASE ACTIVATED RECEPTORS	27	0.009469905
ACTIVATION OF THE PRE REPLICATIVE COMPLEX	29	0.009619904
SIGNALING BY VEGF	11	0.009619904
INTEGRIN ALPHAIIBBETA3 SIGNALING	23	0.009699903
CAM PATHWAY	25	0.009899901
POST TRANSLATIONAL PROTEIN MODIFICATION	39	0.009939901
CELL DEATH SIGNALLING VIA NRAGE NRIF AND NADE	58	0.0100299
NOTCH HLH TRANSCRIPTION PATHWAY	13	0.010729893
PLC BETA MEDIATED EVENTS	37	0.011629884
G1 PHASE	16	0.012059879
ACTIVATION OF THE AP1 FAMILY OF TRANSCRIPTION FACTORS	10	0.012179878
NUCLEAR EVENTS KINASE AND TRANSCRIPTION FACTOR ACTIVATION	24	0.014819852
CD28 CO STIMULATION	29	0.01495985
RECRUITMENT OF NUMA TO MITOTIC CENTROSOMES	9	0.015909841
INTRINSIC PATHWAY FOR APOPTOSIS	28	0.016219838
EARLY PHASE OF HIV LIFE CYCLE	11	0.016759832
REGULATION OF LIPID METABOLISM BY PEROXISOME PROLIFERATOR ACTIVATED RECEPTOR ALPHA	59	0.017239828
METABOLISM OF NITRIC OXIDE	12	0.017549825
ACTIVATED AMPK STIMULATES FATTY ACID OXIDATION IN MUSCLE	17	0.018879811
STEROID HORMONE BIOSYNTHESIS	12	0.020779792

GLUCAGON TYPE LIGAND RECEPTORS	33	0.020829792
G2 M CHECKPOINTS	42	0.021099789
PURINE METABOLISM	30	0.023589764
RNA POLYMERASE III TRANSCRIPTION	32	0.024209758
PLC GAMMA1 SIGNALLING	34	0.024369756
RNA POLYMERASE III TRANSCRIPTION INITIATION FROM TYPE 3 PROMOTER	21	0.025379746
EGFR DOWNREGULATION	22	0.026479735
NRAGE SIGNALS DEATH THROUGH JNK	47	0.026749733
ERK MAPK TARGETS	21	0.02903971
TAT MEDIATED HIV1 ELONGATION ARREST AND RECOVERY	28	0.030679693
ADP SIGNALLING THROUGH P2Y PURINOCEPTOR 12	21	0.030729693
STEROID HORMONES	19	0.030919691
PI3K AKT SIGNALLING	37	0.031249688
G BETA GAMMA SIGNALLING THROUGH PLC BETA	20	0.031289687
CTLA4 INHIBITORY SIGNALING	21	0.035129649
ACTIVATION OF RAC	14	0.035659643
RAS ACTIVATION UOPN CA2+ INFUX THROUGH NMDA RECEPTOR	17	0.036129639
ACTIVATION OF CHAPERONES BY IRE1 ALPHA	9	0.042199578
POLYMERASE SWITCHING	13	0.042259577
MTORC1 MEDIATED SIGNALLING	10	0.044689553
CD28 DEPENDENT PI3K AKT SIGNALING	19	0.046469535
RNA POLYMERASE III CHAIN ELONGATION	11	0.047679523

Table S2. Significantly affected pathways of ESLAM cells upon stimulation with UG26 CM and SF+IL-11.

Signaling pathways	Number of involved genes	Regulation of HSC	References
NGF	208	AGM HSC activity	(Durand et al., 2007)
PDGF	63	HSC expansion	(Su et al., 2002)
WNT	54	HSC proliferation	(Reya et al., 2003; Willert et al., 2003)
EGFR	46	HSC migration	(Ryan et al.)
ROBO Receptor	32	HSC-niche interaction	(Shibata et al., 2009; Smith-Berdan et al.)
BMP	23	AGM HSC activity	(Durand et al., 2007)
Integrin Signaling	23	HSC adhesion	(Benveniste et al., 2010; Notta et al.)
TGF-ß	15	HSC subtype regulation	(Challen et al.)
Notch	14	HSC *de novo* generation	(Kumano et al., 2003)
VEGF	11	HSC survival	(Gerber et al., 2002)

NGF: nerve growth factor, AGM: aorta-gonad-metanephron, PDGF: platelet derived growth factor, EGFR: epidermal growth factor receptor, ROBO: roundabout family, BMP: bone morphogenic protein, TGF-ß: transforming growth factor-beta, VEGF: vascular endothelial growth factor.

Table S3: Secreted factor mRNAs (n=172) produced by UG26 cells with predicted interactions with the products of genes expressed by activated ESLAM cells.

Entrez Gene ID	Gene Symbol	Mean Intensity (Log_2)	Receptor(s)
16785	Rpsa	13.23	Atp5b, Csf2ra, Itga6, Kdelr1
16852	Lgals1	13.20	Cd7, Lgals3bp, Susd2
16952	Anxa1	12.77	Fpr1, Trpm7
15481	Hspa8	12.59	Grb2, Irs1, Cxcr4, Ripk2,Ncor1,Tnfrsf1a, Tnfrsf1b, Cd40, Traf1, Traf2, Atp9b, Ripk3, Kdelr1, Myd88
16854	Lgals3	12.42	Lgals3bp, Cubn, Ncoa3
14683	Gnas	12.32	Grin2b
23980	Pebp1	12.29	Adrbk1, Ppard, Nr2c2
17319	Mif	12.22	Cd74, Tnfrsf14
14456	Gas6	12.18	Mertk, Tyro3
12261	C1qbp	12.14	Gab1, Gabrb1, Tnfrsf1a, Tnfrsf1b, Traf1, Ripk3
22166	Txn1	12.12	Nr3c1
12631	Cfl1	12.11	Grb2
15519	Hsp90aa1	12.03	Alk, Ahr, Aip, Ar, Asgr1, Arntl, Esr1, Nr3c1, Nr3c2, Kdr, Ppara, Tnfrsf1a, Nr2c2, Traf1, Traf2, Ripk3, Ripk2
227753	Gsn	11.86	Ar, Grb2, Grin2b
12306	Anxa2	11.85	Grb2
14219	Ctgf	11.85	Erbb4, Lrp1, Itga5
14115	Fbln2	11.77	Itgb3, Nsd1
18787	Serpine1	11.74	Lrp2, Lrp1, Thbd, Vtn, Lrp1b
21858	Timp2	11.70	Itga3, Pgrmc1
18073	Nid1	11.55	Itgav, Itgb3, Lgals3bp, Ptprf, Notch1
12010	B2m	11.45	Fcgrt, Tfrc
19156	Psap	11.37	Celsr1
21825	Thbs1	11.01	Cd36, Scarb2, Lrp1, Lrp5, Itgb3, Tnfrsf11b
12317	Calr	10.96	Ar, Nr3c1, Itga2b, Itga3, Itgav, Lrp1
12847	Copa	10.88	Pdgfrb, Mtnr1b
20315	Cxcl12	10.78	Cxcr4, Cxcr7, Dpp4
12827	Col4a2	10.78	Cd44, Cd93, Antxr2
21859	Timp3	10.69	Kdr
12826	Col4a1	10.68	Cd44, Cd93
14828	Hspa5	10.65	Atp5b, Scarb2, Htr3a, Ldlr, Grb2, Gria1, Tnfrsf1a, Tnfrsf1b, Traf2, Mtnr1b, Ripk3, Myd88
12833	Col6a1	10.61	Cd44, Lgals3bp
20296	Ccl2	10.52	Ccr2
12842	Col1a1	10.47	Cd44, Cd36, Itga2, Itga5, Cd93, Ddr2
231887	Pdap1	10.39	Pdgfrb
13024	Ctla2a	10.29	Tinagl1
16423	Cd47	10.29	Itgav, P2ry2
14751	Gpi1	10.25	Amfr
12331	Cap1	10.18	Traf3
13722	Aimp1	10.17	Slc20a1

17387	Mmp14	10.13	Lrp1, Itgav
12832	Col5a2	10.06	Cd44
14205	Figf	9.97	Kdr, Flt4, Itga9
13003	Vcan	9.97	Itga4
21814	Tgfbr3	9.96	Acvr2a, Tgfbr2, Tgfbr1
56348	Hsd17b12	9.93	Slc7a1
14268	Fn1	9.91	Cd44
14313	Fst	9.85	Spsb1
16007	Cyr61	9.74	Itgav
12977	Csf1	9.67	Csf1r, Celsr3, Slc7a1, Myd88
20306	Ccl7	9.63	Ccr2, Ccr1, Ccr1l1
226519	Lamc1	9.47	Cd44, Sv2a, Sv2b, Sv2c
14825	Cxcl1	9.31	Cxcr2
21923	Tnc	9.28	Itga9, Itga5, Itgb6, Ptprb, Itga8
12843	Col1a2	9.26	Cd44, Cd36, Itga2, Itga2b, Itgb3, Cd93
57914	Crlf2	9.23	Il7r
19242	Ptn	9.23	Alk, Gnb2l1, Ptprb, Ptprz1, Ryr1
22341	Vegfc	9.20	Kdr, Flt4
14423	Galnt1	9.18	Ptprf
21826	Thbs2	9.13	Cd36
15530	Hspg2	9.08	Itga2
16779	Lamb2	9.00	Cd44, Sv2a, Sv2b, Sv2c
20377	Sfrp1	8.92	Fzd6
12834	Col6a2	8.87	Cd44
16323	Inhba	8.76	Acvr2a, Acvr2b, Acvr1, Tgfbr3
12830	Col4a5	8.75	Cd44, Cd93
53381	Prdx4	8.71	Atp5b
19039	Lgals3bp	8.65	Phb2
54635	Pdgfc	8.63	Pdgfra
12159	Bmp4	8.59	Bmpr1a, Bmpr1b, Bmpr2
16412	Itgb1	8.56	Ptch2
76737	Creld2	8.54	Chrna4, Chrnb2
22340	Vegfb	8.50	Flt1, Nrp1
110611	Hdlbp	8.49	Gnb2l1, Ptch2
12831	Col5a1	8.42	Cd44, Lgals3bp
20348	Sema3c	8.41	Nrp1
19128	Pros1	8.41	Tyro3
18590	Pdgfa	8.40	Pdgfra
16777	Lamb1	8.39	Cd44, Sv2a, Sv2b, Sv2c
14178	Fgf7	8.38	Fgfr3, Fgfr4, Nrp1
13138	Dag1	8.23	Grb2, Rapsn, Musk
11883	Arsa	8.23	Bmpr2
12931	Crlf1	8.22	Cntfr
13874	Ereg	8.19	Erbb4
14600	Ghr	8.14	Grb2, Irs1, Ncoa6
18208	Ntn1	8.09	Adora2b, Dcc, Neo1, Unc5c
56213	Htra1	7.98	Grb2
17311	Kitl	7.89	Kit
21803	Tgfb1	7.85	Acvrl1, Itgav, Itgb6, Tgfbr1, Tgfbr2, Tgfbr3, Vtn
16956	Lpl	7.85	Lrp2, Lrp1
13614	Edn1	7.85	Ednra, Ednrb

17295	Met	7.81	Grb2, Gab1, Ptprb, Ptprj, Spsb1, Itgb4, Plxnb1
11535	Adm	7.76	Calcrl, Gpr182
12837	Col8a1	7.71	Itga1, Itga2, Efemp2
22418	Wnt5a	7.70	Fzd1, Fzd5, Ror2, Lrp6, Ryk
13038	Ctsk	7.70	Fgfr3
15200	Hbegf	7.63	Erbb4
17388	Mmp15	7.53	Lrp1
12064	Bdnf	7.52	Esr1, Sort1
114249	Npnt	7.49	Itga8
16835	Ldlr	7.40	Ldlrap1, Lrpap1, Flt1
12825	Col3a1	7.36	Cd44, Ddr2
433375	Creg1	7.33	Igf2r
11486	Ada	7.27	Adora1, Adora2a, Adora2b, Dpp4, Drd1a, Grb2, Nr3c1
20563	Slit2	7.26	Robo2
16880	Lifr	7.23	Cntfr, Il31ra
12822	Col18a1	7.23	Kdr, Itga5
20564	Slit3	7.17	Robo2
30878	Apln	7.14	Aplnr
15925	Ide	7.13	Ar, Nr3c1
100952	Emilin1	7.11	Tgfbr2
16194	Il6ra	7.00	Erap1
16403	Itga6	6.99	Cd36, Grb2, Itgb4
19206	Ptch1	6.97	Smo
11826	Aqp1	6.94	Trip6, Efemp2
67573	Loxl4	6.89	Trip13
21827	Thbs3	6.88	Cd36
12475	Cd14	6.87	Tlr3, Itgb2, Lgals3bp, Tlr4, Tlr2, Itgam
20210	Saa3	6.71	Fpr1
20350	Sema3f	6.68	Nrp1
11491	Adam17	6.66	Erbb4, Notch1, Ptpn3
94216	Col4a6	6.65	Cd44, Cd93
16190	Il4ra	6.64	Gnb2l1, Il13ra1, Irs1, Irs2, Cd40
18049	Ngf	6.62	Sort1, Ngfr, Ntrk1
20300	Ccl25	6.59	Ccr9, Ccr10, Ccbp2
22417	Wnt4	6.57	Fzd6
56708	Clcf1	6.54	Cntfr, Crlf1
21808	Tgfb2	6.48	Tgfbr1, Tgfbr2, Tgfbr3, Vtn
21802	Tgfa	6.37	Erbb4, Rhbdf1
12835	Col6a3	6.30	Cd44
20312	Cx3cl1	6.28	Cx3cr1
14172	Fgf18	6.23	Fgfr3, Fgfr4
20349	Sema3e	6.21	Plxnd1
13848	Ephb6	6.19	Grb2, Ephb1
22339	Vegfa	6.14	Flt1, Kdr, Grin2b
16173	Il18	6.14	Il1rl2, Il18rap, Il18r1
16819	Lcn2	6.11	Lrp2
53623	Gria3	6.10	Gria2
22403	Wisp2	6.07	Igf1r, Igf2r
22413	Wnt2	6.04	Fzd1, Fzd9
17087	Ly96	6.02	Tlr4, Tlr2
20310	Cxcl2	5.95	Cxcr2

20440	St6gal1	5.94	Cd22
21809	Tgfb3	5.89	Acvrl1, Tgfbr1, Tgfbr2, Tgfbr3
18133	Nov	5.84	Notch1
22042	Tfrc	5.79	Gabarap
16975	Lrp8	5.75	Grin1
20660	Sorl1	5.67	Lrpap1
11815	Apod	5.63	Lepr, Atp5b, Scarb2
14566	Gdf9	5.40	Acvr2a, Bmpr1a, Bmpr1b, Bmpr2
21950	Tnfsf9	5.24	Tnfrsf9, Traf1, Traf2
13214	Defb1	5.23	Ccr6
19143	St14	5.19	F2rl1
16193	Il6	5.14	Il6ra, Hrh1
17082	Il1rl1	5.13	Myd88
13636	Efna1	5.10	Epha1, Epha2, Epha3, Epha4, Epha6, Epha7, Epha8, Ephb1, Tgfbr1
53867	Col5a3	4.91	Cd44
16180	Il1rap	4.88	Irak1, Il1r1
16878	Lif	4.85	Lifr
20750	Spp1	4.68	Itga9, Itga5, Itgav
16196	Il7	4.60	Il7r
18591	Pdgfb	4.31	Pdgfra, Pdgfrb
16169	Il15ra	4.30	Traf2, Il2rb
16000	Igf1	4.26	Igf1r, Igsf1
53603	Tslp	4.05	Il7r, Crlf2
11600	Angpt1	3.90	Itga5, Tek
12223	Btc	3.90	Erbb4, Egfr
11602	Angpt4	3.76	Tek
71785	Pdgfd	3.38	Pdgfrb
53604	Zpbp	3.34	Zp2
110312	Pmch	3.24	Mchr1
12156	Bmp2	3.22	Acvr1, Bmpr1b, Bmpr2, Bmpr1a
13645	Egf	3.19	Egfr, Erbb3, Grb2, Vtn

References:

Benveniste, P., Frelin, C., Janmohamed, S., Barbara, M., Herrington, R., Hyam, D., and Iscove, N.N. (2010). Intermediate-term hematopoietic stem cells with extended but time-limited reconstitution potential. Cell Stem Cell *6*, 48-58.

Challen, G.A., Boles, N.C., Chambers, S.M., and Goodell, M.A. Distinct hematopoietic stem cell subtypes are differentially regulated by TGF-beta1. Cell Stem Cell *6*, 265-278.

Croft, D., O'Kelly, G., Wu, G., Haw, R., Gillespie, M., Matthews, L., Caudy, M., Garapati, P., Gopinath, G., Jassal, B.*, et al.* Reactome: a database of reactions, pathways and biological processes. Nucleic Acids Res *39*, D691-697.

Durand, C., Robin, C., Bollerot, K., Baron, M.H., Ottersbach, K., and Dzierzak, E. (2007). Embryonic stromal clones reveal developmental regulators of definitive hematopoietic stem cells. Proc Natl Acad Sci U S A *104*, 20838-20843.

Dykstra, B., Kent, D., Bowie, M., McCaffrey, L., Hamilton, M., Lyons, K., Lee, S.J., Brinkman, R., and Eaves, C. (2007). Long-term propagation of distinct hematopoietic differentiation programs in vivo. Cell Stem Cell *1*, 218-229.

Gentleman, R.C., Carey, V.J., Bates, D.M., Bolstad, B., Dettling, M., Dudoit, S., Ellis, B., Gautier, L., Ge, Y., Gentry, J.*, et al.* (2004). Bioconductor: open software development for computational biology and bioinformatics. Genome Biol *5*, R80.

Gerber, H.P., Malik, A.K., Solar, G.P., Sherman, D., Liang, X.H., Meng, G., Hong, K., Marsters, J.C., and Ferrara, N. (2002). VEGF regulates haematopoietic stem cell survival by an internal autocrine loop mechanism. Nature *417*, 954-958.

Kent, D., Dykstra, B., and Eaves, C. (2007). Isolation and assessment of long-term reconstituting hematopoietic stem cells from adult mouse bone marrow. Curr Protoc Stem Cell Biol *Chapter 2*, Unit 2A 4.

Kumano, K., Chiba, S., Kunisato, A., Sata, M., Saito, T., Nakagami-Yamaguchi, E., Yamaguchi, T., Masuda, S., Shimizu, K., Takahashi, T.*, et al.* (2003). Notch1 but not Notch2 is essential for generating hematopoietic stem cells from endothelial cells. Immunity *18*, 699-711.

Notta, F., Doulatov, S., Laurenti, E., Poeppl, A., Jurisica, I., and Dick, J.E. Isolation of single human hematopoietic stem cells capable of long-term multilineage engraftment. Science *333*, 218-221.

Reya, T., Duncan, A.W., Ailles, L., Domen, J., Scherer, D.C., Willert, K., Hintz, L., Nusse, R., and Weissman, I.L. (2003). A role for Wnt signalling in self-renewal of haematopoietic stem cells. Nature *423*, 409-414.

Ryan, M.A., Nattamai, K.J., Xing, E., Schleimer, D., Daria, D., Sengupta, A., Kohler, A., Liu, W., Gunzer, M., Jansen, M.*, et al.* Pharmacological inhibition of EGFR signaling enhances G-CSF-induced hematopoietic stem cell mobilization. Nat Med *16*, 1141-1146.

Shibata, F., Goto-Koshino, Y., Morikawa, Y., Komori, T., Ito, M., Fukuchi, Y., Houchins, J.P., Tsang, M., Li, D.Y., Kitamura, T.*, et al.* (2009). Roundabout 4 is expressed on hematopoietic stem cells and potentially involved in the niche-mediated regulation of the side population phenotype. Stem Cells *27*, 183-190.

Smith-Berdan, S., Nguyen, A., Hassanein, D., Zimmer, M., Ugarte, F., Ciriza, J., Li, D., Garcia-Ojeda, M.E., Hinck, L., and Forsberg, E.C. Robo4 cooperates with CXCR4 to specify hematopoietic stem cell localization to bone marrow niches. Cell Stem Cell *8*, 72-83.

Smyth, G.K., Michaud, J., and Scott, H.S. (2005). Use of within-array replicate spots for assessing differential expression in microarray experiments. Bioinformatics *21*, 2067-2075.

Stark, C., Breitkreutz, B.J., Reguly, T., Boucher, L., Breitkreutz, A., and Tyers, M. (2006). BioGRID: a general repository for interaction datasets. Nucleic acids research *34*, D535-539.

Su, R.J., Zhang, X.B., Li, K., Yang, M., Li, C.K., Fok, T.F., James, A.E., Pong, H., and Yuen, P.M. (2002). Platelet-derived growth factor promotes ex vivo expansion of CD34+ cells from human cord blood and enhances long-term culture-initiating cells, non-obese diabetic/severe combined immunodeficient repopulating cells and formation of adherent cells. Br J Haematol *117*, 735-746.

Subramanian, A., Tamayo, P., Mootha, V.K., Mukherjee, S., Ebert, B.L., Gillette, M.A., Paulovich, A., Pomeroy, S.L., Golub, T.R., Lander, E.S., *et al.* (2005). Gene set enrichment analysis: a knowledge-based approach for interpreting genome-wide expression profiles. Proc Natl Acad Sci U S A *102*, 15545-15550.

Willert, K., Brown, J.D., Danenberg, E., Duncan, A.W., Weissman, I.L., Reya, T., Yates, J.R., 3rd, and Nusse, R. (2003). Wnt proteins are lipid-modified and can act as stem cell growth factors. Nature *423*, 448-452.

Yellaboina, S., Dudekula, D.B., and Ko, M. (2008). Prediction of evolutionarily conserved interologs in Mus musculus. BMC genomics *9*, 465.
